# Supplementary material for: Analysis of the economic burden of diagnosis and treatment on patients with tuberculosis in Bao’an district of Shenzhen City, China
Source: PLoS One. 2020 Aug 31;15(8):e0237865. doi: 10.1371/journal.pone.0237865 (PMC7458315; doi:10.1371/journal.pone.0237865)
Supplement: S1 Table — (DOCX) [file pone.0237865.s001.docx]

**Table S1.** **Confounder adjusted association between costs due to TB diagnosis and various predictor variables using logistic regression models in the study of Bao'an district, Shenzhen City, China, 2013 (N=514)**

| Predictor in the model | Beta coefficient | 95% CI | P value |
| --- | --- | --- | --- |
| Number of times visiting health-care facilities | | | |
| <=2 | Ref | Ref | Ref |
| 2~6 | 2.26 | 5.39,17.24 | <0.001 |
| >=7 | 4.17 | 8.33,506.06 | <0.001 |
| Sex | | | |
| Male | Ref | Ref | Ref |
| Fale | 0.78 | 1.12,4.26 | 0.021 |
| Education | | | |
| Junior high school | -1.62 | 0.07,0.59 | 0.003 |
| Senior high school | -0.95 | 0.16,0.93 | 0.034 |
| College or above | -0.76 | 0.19,1.15 | 0.098 |
| Constant | 43.273 |  | 0.988 |

*Logistc regression was done after the costs were divided as binomial variables based on median costs due to TB dignosis .*

*Only significant variables were presented.*
