# Supplementary material for: Analysis of the economic burden of diagnosis and treatment on patients with tuberculosis in Bao’an district of Shenzhen City, China
Source: PLoS One. 2020 Aug 31;15(8):e0237865. doi: 10.1371/journal.pone.0237865 (PMC7458315; doi:10.1371/journal.pone.0237865)
Supplement: S2 Table — (DOCX) [file pone.0237865.s002.docx]

**Table S2. Confounder adjusted association between *costs due to TB diagnosis* and various predictor variables using logistic regression models in the study of Bao'an district, Shenzhen City, China, 2013 (N=514)**

| Predictor in the model | Beta coefficient | (95% CI) | P value |
| --- | --- | --- | --- |
| Sputum smear status | |  |  |
| Negative | Ref | Ref | Ref |
| Positive | 0.653 | （1.350,2.735） | <0.001 |
| Constant | -0.288 |  | 0.016 |

*Logistic regression was done after the costs were divided as binomial variables based on median costs due to TB treatment.*

*Only significant variables were presented.*
