# Supplementary material for: Analysis of the economic burden of diagnosis and treatment on patients with tuberculosis in Bao’an district of Shenzhen City, China
Source: PLoS One. 2020 Aug 31;15(8):e0237865. doi: 10.1371/journal.pone.0237865 (PMC7458315; doi:10.1371/journal.pone.0237865)
Supplement: S3 Table — (DOCX) [file pone.0237865.s003.docx]

**Table S3. Confounder adjusted association between *costs due to TB care* and various predictor variables using logistic regression models in the study of Bao'an district, Shenzhen City, China, 2013 (N=514)**

| Predictor in the model | Beta coefficient | (95% CI) | P value |
| --- | --- | --- | --- |
| Sputum smear status | |  |  |
| Negative | Ref | Ref | Ref |
| Positive | 85 | （1.33,4.09） | 0.003 |
| Number of times visiting health-care facilities | | | |
| <=2 | Ref | Ref | Ref |
| 2~6 | 0.63 | 1.04,3.93 | 0.038 |
| >=7 | 1.81 | 1.55,24.00 | 0.010 |
| Constant | 41.94 |  | 0.998 |

*Logistic regression was done after the costs were divided as binomial variables based on median costs due to TB care.*

*Only significant variables were presented.*
