# Supplementary material for: Analysis of the economic burden of diagnosis and treatment on patients with tuberculosis in Bao’an district of Shenzhen City, China
Source: PLoS One. 2020 Aug 31;15(8):e0237865. doi: 10.1371/journal.pone.0237865 (PMC7458315; doi:10.1371/journal.pone.0237865)
Supplement: S4 Table — (DOCX) [file pone.0237865.s004.docx]

**Table S4 . Summary of independent predictors associated with costs due to TB diagnosis and treatment and care(diagnosis +treatment) in the study of Bao'an district, Shenzhen City, China, 2013 (n=514)**

| Variable | | Costs due to TB diagnosis | Costs due to TB treatment | Costs due to TB care(diagnosis+treatment) |
| --- | --- | --- | --- | --- |
| Number of times visiting health-care facilities | |  |  |  |
|  | <=2 | Y(-) |  | Y(-) |
|  | 2~6 | Y(-) |  |  |
|  | >=7 | Ref | Ref | Ref |
| Occupation | |  |  |  |
|  | Workers | Ref | Ref | Ref |
|  | Individual business |  |  |  |
|  | Others national civil servant/services /retired staff etc.) | Y(+) |  |  |
|  | Unemployed |  |  |  |
| Education | |  |  |  |
|  | Primary/illiterate | Ref | Ref | Ref |
|  | Junior high school |  |  |  |
|  | Senior high school |  |  |  |
|  | College or above | Y(+) |  |  |
| Marital status | |  |  |  |
|  | Unmarried | Ref | Ref | Ref |
|  | Married |  |  |  |
|  | Widowed/divorced |  | Y(+) |  |
|  | Others |  |  |  |
| Household registration | |  |  |  |
|  | Native patients | Ref | Ref | Ref |
|  | Migrant patients |  | Y(+) | Y(+) |
| Whether in hospital due to TB diagnosis | |  |  |  |
|  | Yes | Y(+) |  | Y(+) |
|  | No | Ref | Ref | Ref |
| Sputum smear status | |  |  |  |
|  | Negative | Ref | Ref | Ref |
|  | Positive |  | Y(+) |  |
| Reported-household economic burden | | |  |  |
|  | Heavy | Ref | Ref | Ref |
|  | Moderate |  | Y(-) |  |
|  | No burden |  |  |  |

*Y – Yes; TB – tuberculosi; Ref-reference ; Y(+) positive sign means the factor is an independent predictor for costs and Y(-) negative sign means the factor is an independent predictor for less costs when compared to reference.*
