# Supplementary material for: Analysis of the economic burden of diagnosis and treatment on patients with tuberculosis in Bao’an district of Shenzhen City, China
Source: PLoS One. 2020 Aug 31;15(8):e0237865. doi: 10.1371/journal.pone.0237865 (PMC7458315; doi:10.1371/journal.pone.0237865)
Supplement: Attachment — Submitted filename: PONE-D-20-06789_R1_hds.pdf [file pone.0237865.s007.pdf]

PLOS ONE

Analysis of the Economic Burden of Diagnosis and Treatment on Patients with non-drug resistance Tuberculosis (TB) in the TB Control Demonstration Area of China

--Manuscript Draft--

Manuscript Number:	PONE-D-20-06789R1
Article Type:	Research Article
Full Title:	Analysis of the Economic Burden of Diagnosis and Treatment on Patients with non-drug resistance Tuberculosis (TB) in the TB Control Demonstration Area of China
Short Title:	Economic Burden of Diagnosis and Treatment on Patients with Tuberculosis
Corresponding Author:	Wei-Qing Chen<br>Sun Yat-sen University<br>GUangzhou, CHINA
Keywords:	Tuberculosis; Cost of illness; Economic burden; China
Abstract:	Background: Illness-related costs experienced by patients produce a severe economic impact on households, especially those of poor families. Few studies have investigated the full costs, including direct and opportunity costs, at the patient and household levels in the south-east of China. Methods: A case follow-up study was conducted in the Bao'an district of Shenzhen City, China. Eligible new and re-treated individuals with pulmonary TB during January 1 st 2013 to June 30 th 2013 were enrolled. Medical and non-medical costs as well as income loss were calculated in pre-diagnosis and post-diagnosis periods, respectively. Factors associated pre-diagnosis costs and post-diagnosis costs were explored respectively with a linear regression model. Results: Of the total 514 TB patients enrolled, most were from the migrant population, and 65% were males (336), with a mean age of 32.25 (±10.11) years. The median pre-diagnosis direct costs, direct post-diagnosis costs, and post-diagnosis medical and non-medical costs were 79USD, 748USD, 542USD, and 150USD (6.2897CNY = 1USD, 2013) per patient, respectively. The median total cost of TB treatment per patient was 1218USD, corresponding to 26% of patients' annual income pre-illness. Variables associated with pre-diagnosis costs included number of times visiting health facilities, whether in hospital due to TB, education, History of TB. Variables associated with post-diagnosis costs were household registration, sputum smear status, reported-household economic burden, marital status. Conclusions: Although a free TB control policy is in force, patients with TB are still facing a heavy economic burden. More available interventions to reduce the financial burden on tuberculosis patients are urgently needed.
Order of Authors:	Yixiang Huang
	Jianying Huang
	Xiaoting Su
	Liang Chen
	Jianwei Guo
	Weiqing Chen
	Lingling Zhang
Opposed Reviewers:	
Response to Reviewers:	We have upload the file "Response to editor and reviewers" as attached file, please check for clearer and more detailed responses . The following is the copy of the file:<br><br>Additional Editor Comments (if provided):<br><br>Dear Authors,<br>In addition to the valuable points raised by the two reviewers, i have some comments from my end (some of these may be made by the reviewers as well)<br>1. Abstract - most were from the migrant population - why use ' most' here while elsewhere the number % have been given?

Powered by Editorial Manager® and ProduXion Manager® from Aries Systems Corporation

	Thanks for the editor's suggestion. We have replaced 'most' with the exact number '95%' in the abstract, which was mentioned in the line 255.<br>2. out of pockets expenditure? Please be careful with use of phrases (point made by the reviewer as well). Are you looking at total costs due to TB care or out of pocket expenditure. Overall in your paper, no reference was made to the WHO TB patient cost survey handbook 2016. I see that the study was among patients registered in 2013 and the WHO ptient cost survey booklet was not out then. Hence, it will be nice ot have a table in methods with clear operational definitions used.<br>We are grateful for your suggestion. In our study, we did not include the cost covered by the medical insurance, hence it is inappropriate to use the term 'out of pockets expenditure'. So we replaced it with 'cost' to make it more accurate. There are many kinds of medical insurance in China, and the patients may not be aware of whether they have certain kind of medical insurance or not, so they did not use the medical insurance to reduce their expenditure. However, in our study, the cost is measurable and comparable, so we revised it throughout this article accordingly.<br>According to your advice, we have added the operational definitions in table 1.<br>3. 2.2 Study design title should be renamed as 2.2 Study design and population<br>Revised, thank you.<br>4. Anonymized data HAS to be shared and this is ESSENTIAL<br>Thanks for this suggestion. We have provided the anonymous data as attachment in an .xls document.<br>5. In the ethics statement, please mention the ethics number and the date<br>We are grateful for the Reviewer's suggestion. The ethical approval number is 2012-SPH021, date 9/28/ 2012.<br>6. Line 175-176 (regarding primary outcome) - this line is not clear<br>Thanks for this suggestion. We revised the definition to make it clear in line 192-193 as follows: The primary outcome variables were the mean (median) expense incurred during illness.<br><br>7. Line 191 - shouldn't it be the household's pre-TB annual income?<br>We appreciate your suggestion. Our study does have the limitation that we collected the range of the household's pre-TB annual income of every patient instead the exact number. However, we collected the exact number of the total annual income of every patient and as a result, we used the indicator to measure the patients' economic burden.<br><br>8. Line 204-06. Similar issues were faced by us in another study. Another analysis may be to classify the costs as catastrophic or not and then do a log binomial regression. We can present both the results. See the following paper for example, as to how the analysis was done: https://www.ncbi.nlm.nih.gov/pubmed/30865730<br>We appreciate this suggestion. As mentioned above, we did not consider the catastrophic cost as an indicator in the design phase of our study, so we are not able to conduct the analysis regarding catastrophic cost. In our revision, based on the paper given above, we improved our method of statistics by adopting 2 methods in our revised manuscript.<br><br>9. Line 206 - Bivariate means two outcomes. All the analyses presented in this paper are univariate which can be further classified into single variable (curde or unadjusted) and multivariable (adjsuted) analysis.<br>Thanks for this suggestion. We have adopted a new method in the data analysis which is described carefully in line 221-244.<br><br>10. What about catastrophic costs, what about inequity in distribution of catastrophic costs. See this paper for example. https://www.ncbi.nlm.nih.gov/pubmed/30173603<br>We appreciate your suggestion. However, we did not adopt this professional term at the start of the study, and the annual patient income is expressed as a range instead of an exact number. As a result, it would be impossible to calculate the catastrophic costs to patients.<br><br>11. All the table and figure title have to be standalone with details about time place and person. Check for footnotes for abbreviations. All tables should be formatted again to make them presentable. For ex n and % in separate columns. n column shuold be right indented, (%) column should be left indented and vertical line between n and % should then be removed. CHeck other tables as well.

Powered by Editorial Manager® and ProduXion Manager® from Aries Systems Corporation

We appreciate your suggestion. We have reformatted the tables throughout the article according to your suggestions.
12. Why don't the authors look separately at factors associated with pre-dx costs and post-diagnosis costs.
Thank you for this suggestion. We have analyzed the factors associated with different period of the treatment according to your suggestion and revised the statement in the data analysis, results and discussion section on the basis of the new analysis results.
13. If the authors have outcome data, why not compare unfav out and catastrophic costs, after adjusting for potential confounders.
We appreciate your suggestion. However, we did not adopt this professional term at the start of the study, and the annual patient income is expressed as a range instead of an exact number. As a result, it would be impossible to calculate the catastrophic costs to patients.

Reviewer #1: This work is on an important area namely the economic burden due to Tuberculosis related to the EndTB strategy goal of Zero Catastrophic cost for households due to Tuberculosis. The author have drafted the manuscript well and included several important points needed for a cost analysis study.

The study utilised a follow-up methodology to capture costs during the treatment phase. The authors have also studied some factors related to costs.

While the study justification rightly draws support from the EndTB Goal of Zero Catastrophic Cost, the authors have not included Catastrophic Cost estimation in the paper, despite having all the necessary data for the calculation of the same. It is highly recommended that the authors may include catastrophic cost and accordingly the manuscript may be redrafted. If there are data available on the coping costs or mechanism, they may also be included to understand the complete picture of the economic burden faced by the patients with Tuberculosis.
We appreciate your suggestion. However, we did not adopt this professional term at the start of the study, and the annual patient income is expressed as a range instead of an exact number. As a result, it would be impossible to calculate the catastrophic costs to patients.
In our study, most of the study population were factory workers. Only 186 workers knew that they had medical insurance and 305 workers knew that they did not have any medical insurance. Other 23 workers were unaware of whether they had medical insurance or not, and at the end of the study, they did not bring about any medical care reimbursement cost. Only 34 patients provided information about medical care reimbursement cost. As a result, the proportion of cost paid by medical insurance was not included in the analysis.

Abstract
Though the study includes both direct and indirect costs, the abstract starts with the terminology ‘expenditures’ which may apparently mislead a casual reader
Follow-up method utilised may be included in the methods under the abstract
Thank you for this suggestion. We have replaced the word ‘expenditures’ with ‘costs’ to avoid misunderstanding and added the follow-up method in the abstract.

Methodology
In the study settings,
the following may be included, which have implications while interpreting of the results:
1.Proportion of Treatment and Retreatment Cases in the District;
Thanks for this suggestion. In the discussion section, we mentioned the proportion of treatment and retreatment cases in line 508-509 as follows: The re-treatment rate in our study was 5.84%, which is similar to that reported in other studies, with a re-treatment rate of 6.7% in Shenzhen and 6.4% in other provinces in China.
2.Actual figures of Incidence and Prevalence of Tuberculosis in Shenzhen Region/Bao’an District (lines 119-120), which needs to be specified seperately further between natives and migrants;
We appreciate your suggestion. We have added the data in line 139-141 as follows: In 2010, the prevalence rate of smear positive of native population was 19.44 per 100 000 population, and that of the migrant population was 59.96 per 100 000 population.
3.No, of TB Cases in the District during the study year among natives and migrants;
Thanks for this suggestion. We have listed the number in line 400-402 as follows: The

total number of registered active tuberculosis cases were 1317, included 76(5.8) native case and 1241(94.2%) migrant cases in 2013.
4.No. of (mandatory)visits as per the existing TB program during treatment;
Thank you for this suggestion. We have added the data in line 157.
5.Proportion of patients with TB having co-morbidities namely diabetes, HIV, lung cancer etc
We appreciate your suggestion. Because in our study, the patients with co-morbidities were excluded from the study, it is impossible to calculate the proportion of patients with TB having co-morbidities.
6.Whether Active Case finding strategy is implemented or not;
We appreciate your suggestion. In the study setting section, we have added the statements in line 132-139 to clarify the way of TB patients being found in China as follows: The hospitals were the main place finding tuberculosis case and the Center for Prevention and Cure of chronic diseases (CPC) were the local designated TB facilities in the national TB programme networks, providing treatment and managing the tuberculosis cases. The healthcare providers in general hospitals were obligate to refer the suspected cases to CPC and the basic information of suspected cases were sent to CPC. If the cases did not consulted to CPC in time, the workers of CPC would contact the suspected case. Actively cooperating with medical institutions and TB prevention and control institutions, the discovery rate and report quality was increasing by years.
7.Mean/Median household (and/or Individual) income in the region/district;
Thank you for this suggestion. We have added the median household income in Bao'an district in line 155-157 as follows: The mean household income in the region was 6,656.79 USD of 2012 according to the Statistical yearbook 2013.
8.Proportion of people in different economic categories;
We appreciate your suggestion. However, we did not have access to the detailed information about the income of different population.
9.a brief description of occupational health care provisions/model in the district relevant to TB care;
Thanks for this suggestion. In China, there is no occupational health care provisions/model focus on TB prevention only, the workers in our study were provided with the general occupational health service. For example, the workers may participant in the regular medical examination paid by the factory owner.
10.brief description of the existing intervention in the district to address economic burden faced by patients/households due to TB
We appreciate your suggestion. In line 148-151, we described the existing free policies to relief the economic burden on TB patients: The Center for Prevention and Cure of chronic diseases (CPC) is the institution authorized to provide TB diagnosis, treatment, and monitoring. The CPC provides patients with a radiologic imaging studies (X-ray) while diagnosed, and sputum smear tests during treatment, and anti-TB drugs for 6-8 months for free.
11.whether hospitalisation costs due to TB for persons with TB are covered under the existing program?
Thank you for this suggestion. The hospitalization costs due to TB was not covered under the existing program and born by the patients themselves. We have added the statements in line 148-151 to state the range of free TB policy. Since the cost regarding hospitalization is not included in the policy, it have to be borne by patients themselves.

Study Design:
Why were those aged above 59 years not included for the study? Reasons may be stated and also included/addressed in the discussion.
We appreciate your suggestion. People ages above 59 are more likely to have comorbidities which may influence the analysis of economic burden, so we excluded them. We have added the reason in line 166-167 as follows: The patients aged above 59 were excluded considering the elderly may have more commodities and those with MDR-TB were excluded too.
In the discussion, need to consider, how non-inclusion of those with morbidity namely Diabetes, HIV affected the representativeness of the study. HIV and Diabetes have programmatic implications for Tuberculosis control as well as have been shown by studies in the literature to drive the costs due to tuberculosis higher.
Thanks for this suggestion. We have discussed the influence of non-inclusion on the representativeness of our study in the limitation section in line 392-398 as follows: We

Powered by Editorial Manager® and ProduXion Manager® from Aries Systems Corporation

included 533 eligible patients in the survey, 19 of which were lost to follow-up. The average age of them was 29.6±10.2years. The male was 14 and female was 5. One patient was with a severe symptoms appeared hemoptysis. The others were with mild or moderate symptoms like cough, chest distress and fever etc. the lost to follow-up was about 4 percent, and the general Socio-demographic and clinical characteristics of those 19 participants lost to follow-up were identical with the 514 patients. So，we considered there no much influence on our conclusion.
However, the exclusion can inevitably injure the representativeness, so we added this point as a limitation of our study in line 519-523 as follows: Our study only investigated the economic burden of patients brought by TB and those with severe co-morbidities such as AIDS, diabetes, and lung cancer were excluded. Therefore our study may reflect the economic burden faced by TB patients instead of all population.
Where those with MDR-TB excluded? Or is it that there were no drug resistant cases during the study period, which is quite unlikely. The authors have not stated whether the study is about costs due to drug sensitive tuberculosis. These needs to be clarified in title, abstract, justification (objectives), methods, and discussion.
Thank you for this suggestion. We have added the statement in the Study Design and population part in line165-167. To illustrate the study population of our survey more accurate, we have added the particular explanation in every section accordingly.
In our study, there were only 4 patients with MDR-TB among total 514 patients. One patient failed to achieve TB suppression in the third month of treatment, and the other three patients developed drug resistance during the treatment. Doctors adjusted the medicine according to their condition and part of second-line medicine is not free. Because there were few patients with MDR-TB, we did not eliminated them from the analysis. However, they may not have much impact on the stability of the analysis.
Sample Size has not been estimated by the authors. Studies in the literature on Cost of illness have adopted different methods to calculate sample size. They may be referred to.
We appreciate your suggestion. Ahead of our study, we did not calculate the sample size we needed. We included every patient who met the inclusion criteria and agreed to take part in our survey during the study period. Analysis results showed that the sample size of our study is enough to achieve the power required.
The cost data was based on self-reporting. The authors have mentioned both in the methodology and strengths in discussion, that that a logic-check was done. Brief description of this may be provided to help readers understand how this enabled better data quality.
We appreciate your suggestion. In the Data collection section, we have explained the process of a logic-check in line 187-189 as follows: Those contradictions or missing information would be asked of patients again in a subsequent interview. We also evaluated the quality of collected data by verifying the routine health information system.
Timing of the first interview in the intensive phase could have affected the recall leading to differential recall among different participants. The data on delay between start of treatment and interview may be provided as mean and range. Similarly, it would be important to state whether the timing of multiple interviews were standardised for all patients.
Thank you for this suggestion. First interview is in the first month of intensive phase treatment. We have added the median delay time of diagnosis in line 270-271. In the limitation section, we have mentioned that recall bias could not be avoided in our study as it relied on the information self-reported by patients. Patients went to see the doctor in chronic disease station nearby on time and were followed-up during their visit.
Methodology can include explicitly the perspective from which cost was calculated, though it is understandable from the objective.
Thanks for this suggestion. We have added the illustration in the line 192 as follows: We analysis the economic burden from tuberculosis patients' perspective.
The reason for non-inclusion of guardian (companion) cost is not acceptable as the need for guardian are often higher during pre-diagnosis phase as the patients have more illness and may often make multiple visits to one or more provider before the diagnosis and start of treatment. Authors have also reported hospitalisations during pre-diagnosis period. Guardian (companion) costs needs to be included in the analysis.
We appreciate your suggestion. We regret mentioning that the companion costs was not surveyed in the study. In China, TB is a common disease and most patients do not need companions during the treatment. Another reason is that patients in our study

Powered by Editorial Manager® and ProduXion Manager® from Aries Systems Corporation

were surveyed in the community or chronic disease station near their home within 15 minutes' walk. So the companion costs during pre-diagnosis period may not have much impact on the outcome. We admit that it is a limitation of our study and have added the explanation in line 523-525 as follows: Second, in our study, we did not survey the information regarding companion costs although it may not have much impact on the results since most TB patients in China did not need to be companion during the treatment.

The source of questionnaire may be stated and provided.

Thanks for your suggestion. In this survey, we combined the aim of our study with standardized questionnaire used to investigate the demographic characteristic and designed the questionnaire. We have provided the questionnaire we used as attachment.

Though the study reported insurance coverage for participants, authors have not stated how reimbursements were adjusted against the costs in the analysis.

Thanks for your suggestion. In our study, most of the study population were factory workers. Only 186 workers knew that they had medical insurance and 305 workers knew that they did not have any medical insurance. Other 23 workers were unaware of whether they had medical insurance or not, and at the end of the study, they did not bring about any medical care reimbursement cost. Only 34 patients provided information about medical care reimbursement cost. As a result, the proportion of cost paid by medical insurance was not included in the analysis.

Results

Clinical characteristics including co-morbidities, pulmonary/extrapulmonary, etc to be included.

Care-seeking characteristics should include number of consultations/visits prior to diagnosis, number of providers visited and type of providers / facility visited (especially private providers /facility as it can vary from single doctor provider facility to corporate hospital)

We appreciate your suggestion. In the Socio-demographic Characteristics and Care-seeking Behaviors section, we have added the number of consultations prior diagnosis and type of facility visited in line 267-270. However, we did not collect information about the number of providers visited.

Data on whether the participant was a beneficiary of any of the existing interventions in the district to reduce the economic burden to patients due to TB, needs to be reported, including the quantum of benefit

Thanks for your suggestion. We have mentioned the coverage of the existing free TB policy in the Study setting section in line 149-151. In the second paragraph, we have also added the description of the extent of free treatment and the cost covered by the policy in line 404-406 as follows: Under the DOTS program, the CPC provide tuberculosis patients with a radiologic imaging studies (X-ray) , sputum smear tests and anti-TB drug during treatment for free amounting to 102.07 USD.

The cost during different phases of treatment could also be made available especially since authors have conducted multiple interviews through follow-up to obtain costs through the treatment period. They are important while planning interventions

Where there no hospitalisation episodes among participants during the treatment phase?

We appreciate your suggestion. Our study only investigated the cost reported by the patients occurred in the chronic disease station. There was no inpatient ward in the chronic disease station, so we did not surveyed the information about hospitalization.

In Table 2, what are these Non-Tb drugs?

Thank you for this suggestion. We have added the subsidiary drugs in line 152-153.

In table 3, what timeline does sputum smear test pertain to? What is the definition of household economic burden? What is the definition for degree of symptoms at diagnosis? Is it self-reported or based on clinical assessment? The Operational definition of these variables need to be included in the methodology.

Thanks for this suggestion. We have illustrated these definitions clearly in table 1.

Why insurance coverage was not included as a factor for economic burden?

Thanks for your suggestion. In our study, most of the study population were factory workers. Only 186 workers knew that they had medical insurance and 305 workers knew that they did not have any medical insurance. Other 23 workers were unaware of whether they had medical insurance or not, and at the end of the study, they did not

bring about any medical care reimbursement cost. Only 34 patients provided information about medical care reimbursement cost. As a result, the proportion of cost paid by medical insurance was not included in the analysis.
Among the factors considered for economic burden, some of them pertain to pre-diagnosis costs and other may pertain to treatment phase costs and few many be for total costs. It is recommended that these be split accordingly instead of considering everything together, as there are implications for suggesting possible interventions to reduce cost.
We appreciate this suggestion. Considering everything together is truly not legible and easy to guide practice. So we decided to analyze the factors associated with pre-diagnosis and post-diagnosis separately according to your suggestion.
Discussion

While the population of the district has 65% migrants, in the study, 95% of the study participants are migrants. This can have implications for representativeness of the study. This needs to be discussed in detail; this again importance further since it is one of the factors found to be significantly associated with economic burden as in table 3.
Thanks for this suggestion. We have discussed it carefully in line 402-405 as follows:
As one of the most population of Shenzhen City and owing numbers of factories , the migrant population in Bao'an district always exceeds the average level of Shenzhen City. The total number of registered active tuberculosis cases were 1317, included 76(5.8%) native case and 1241(94.2%) migrant cases in 2013. In our study, 95 percent were migrant cases, so our study can reflect the TB condition of Bao'an district truly.
Socio-demographic and clinical characteristics of those 19 participants lost to follow-up need to be reported and the implications of the study results need to be discussed
We appreciate this suggestion. In line 395-401, we have added the statement of the influence of lost follow-ups on our outcome.
Of the 533 participants, 176 patients were reported to have been shifted to CPC with no costs for patients during pre-diagnosis period. Can description of these transfers provided and included in study setting. How this lead to no delays and no costs are important for understanding the drivers of costs/economic burden.
Thank you for this suggestion. We have added the detailed illustration in the study setting section in line 132-139.
Line 413, what are the author establishing by stating 'recall and reporting bias could not be avoided, even in this longitudinal design' The statement is self-contradictory. May need to be revised
Revised, thank you.
Figure may not be needed as this information is represented in the table 2 and is clearly evident too.
We appreciate this suggestion. We deleted figure 1 in the article.
Some Typographical Errors
Alignment of 70% in Age category 40-50 in Table 1
Subtitle Insurance to be made bold in Table 1
Weblink in line 112 may be shifted to reference and reference number provided
Title of Table 3, CNY needs to be removed
Revised, thank you.
Reviewer #2: 1. The author needs to add some more details on opportunity cost i.e, income loss by patients due to illness. Some patients were hospitalized but they have earnings from job without wage loss then how to quantify the opportunity cost? Also, variation in salary is very heterogenetic. The author needs to describe about opportunity cost.
We appreciate this suggestion. We have describe the opportunity cost in table 1 carefully. If TB patients still worked during the hospitalization, then there was no wage loss and the wage loss was 0.
2. Under background characteristics, author mentioned about insurance company, but for Out of Pocket Expenditure (OOPE), we should not mention reimbursements given by insurance company. If the patients are getting reimbursements from any type of insurance, it should be deducted from the total out of pocket expenditure.
Net out of pocket expenditure = Total expenditure - Reimbursement.
Reimbursement amount should be highlighted if possible.
Thank you for this suggestion. We realized that it was inappropriate to use the term 'out of pocket expenditure' to describe the economic burden for in our study, the cost covered by medical insurance is not included in the analysis. In our study, most of the study population were factory workers. Only 186 workers knew that they had medical

Powered by Editorial Manager® and ProduXion Manager® from Aries Systems Corporation

	insurance and 305 workers knew that they did not have any medical insurance. Other 23 workers were unaware of whether they had medical insurance or not, and at the end of the study, they did not bring about any medical care reimbursement cost. Only 34 patients provided information about medical care reimbursement cost. As a result, the proportion of cost paid by medical insurance was not included in the analysis.<br>3. In methodology section, the author has mentioned the Chi-square test and Mann-Whitney analysis to examine the effect of each predictor variable on economic burden. But Chi-square test is used to know the association between predictor variables and regression analysis is used to understand the effects of predictor variables.<br>We appreciate your suggestion. We have revised the analysis method and Chi-square test is not used this time. So we deleted it.<br>4. Table 3 analysis indicated that factors related to direct and opportunity costs (CNY) and in relation with the background variables. The heading should be association or differentials by background characteristics of patients and table 4 is showing the factor with regression analysis.<br>We have revised the table titles according to your advice and thanks for your advice.<br>5. In table 4 multivariate analysis, only few predictor variables have been added. However, the author described in methodology section A forward stepwise approach was used to find the appropriate model. If P-value less than 0.05 is significant. It is good and recognised methods for adding the variables in regression analysis. Try also Variance inflation factor (VIF) to know the multicollinearity in predictor variables. If VIF less than 10, than the author can add the predictor variables and explore more variable as predictor variables.<br>Thank you for this suggestion. We have adjusted the data analysis method according to your suggestion.<br>Based on the above observations and looking into overall merit of the paper, few modifications in line of above points may be considered before sending it for publication.
**Additional Information:**	
**Question**	**Response**
**Financial Disclosure**<br><br>Enter a financial disclosure statement that describes the sources of funding for the work included in this submission. Review the submission guidelines for detailed requirements. View published research articles from *PLOS ONE* for specific examples.<br><br>This statement is required for submission and **will appear in the published article** if the submission is accepted. Please make sure it is accurate.	This project was funded by the National Major Science and Technology Programs in the "Twelfth Five-Year" Plan period (No. 2012ZX10004903). The funders had no role in study design, data collection and analysis, decision to publish, or preparation of the manuscript. The funders had no role in study design, data collection and analysis, decision to publish, or preparation of the manuscript.

**Unfunded studies**<br>Enter: *The author(s) received no specific funding for this work.*<br><br>**Funded studies**<br>Enter a statement with the following details:<br>• Initials of the authors who received each award<br>• Grant numbers awarded to each author<br>• The full name of each funder<br>• URL of each funder website<br>• Did the sponsors or funders play any role in the study design, data collection and analysis, decision to publish, or preparation of the manuscript?<br>• **NO** - Include this sentence at the end of your statement: *The funders had no role in study design, data collection and analysis, decision to publish, or preparation of the manuscript.*<br>• **YES** - Specify the role(s) played.<br><br>* typeset	
**Competing Interests**<br><br>Use the instructions below to enter a competing interest statement for this submission. On behalf of all authors, disclose any competing interests that could be perceived to bias this work—acknowledging all financial support and any other relevant financial or non-financial competing interests.<br><br>This statement **will appear in the published article** if the submission is accepted. Please make sure it is accurate. View published research articles from *PLOS ONE* for specific examples.	The authors have declared that no competing interests exist.

**NO authors have competing interests**<br><br>Enter: *The authors have declared that no competing interests exist.*<br><br>**Authors with competing interests**<br><br>Enter competing interest details beginning with this statement:<br><br>*I have read the journal's policy and the authors of this manuscript have the following competing interests: [insert competing interests here]*<br><br>* typeset	
**Ethics Statement**<br><br>Enter an ethics statement for this submission. This statement is required if the study involved:<br><br>• Human participants<br>• Human specimens or tissue<br>• Vertebrate animals or cephalopods<br>• Vertebrate embryos or tissues<br>• Field research<br><br>Write "N/A" if the submission does not require an ethics statement.<br><br>General guidance is provided below. Consult the submission guidelines for detailed instructions. **Make sure that all information entered here is included in the Methods section of the manuscript.**	This study was approved by the research and ethics committee of the School of Public Health, Sun Yat-Sen University, China. The ethical approval number is 2012-SPH021, date 9/28/ 2012. Also, permission was obtained from the study site. A signed informed consent was obtained from all patients before being interviewed.

Powered by Editorial Manager® and ProduXion Manager® from Aries Systems Corporation

**Format for specific study types**<br><br>**Human Subject Research (involving human participants and/or tissue)**<br>• Give the name of the institutional review board or ethics committee that approved the study<br>• Include the approval number and/or a statement indicating approval of this research<br>• Indicate the form of consent obtained (written/oral) or the reason that consent was not obtained (e.g. the data were analyzed anonymously)<br><br>**Animal Research (involving vertebrate animals, embryos or tissues)**<br>• Provide the name of the Institutional Animal Care and Use Committee (IACUC) or other relevant ethics board that reviewed the study protocol, and indicate whether they approved this research or granted a formal waiver of ethical approval<br>• Include an approval number if one was obtained<br>• If the study involved *non-human primates*, add *additional details* about animal welfare and steps taken to ameliorate suffering<br>• If anesthesia, euthanasia, or any kind of animal sacrifice is part of the study, include briefly which substances and/or methods were applied<br><br>**Field Research**<br><br>Include the following details if this study involves the collection of plant, animal, or other materials from a natural setting:<br>• Field permit number<br>• Name of the institution or relevant body that granted permission	
**Data Availability**<br><br>Authors are required to make all data underlying the findings described fully available, without restriction, and from the time of publication. PLOS allows rare exceptions to address legal and ethical concerns. See the PLOS Data Policy and FAQ for detailed information.	No - some restrictions will apply

A Data Availability Statement describing where the data can be found is required at submission. Your answers to this question constitute the Data Availability Statement and **will be published in the article**, if accepted.<br><br>**Important:** Stating 'data available on request from the author' is not sufficient. If your data are only available upon request, select 'No' for the first question and explain your exceptional situation in the text box.<br><br>Do the authors confirm that all data underlying the findings described in their manuscript are fully available without restriction?	
**Describe where the data may be found in full sentences. If you are copying our sample text, replace any instances of XXX with the appropriate details.**<br><br>• If the data are **held or will be held in a public repository**, include URLs, accession numbers or DOIs. If this information will only be available after acceptance, indicate this by ticking the box below. For example: *All XXX files are available from the XXX database (accession number(s) XXX, XXX.*).<br>• If the data are all contained **within the manuscript and/or Supporting Information files**, enter the following: *All relevant data are within the manuscript and its Supporting Information files.*<br>• If neither of these applies but you are able to provide **details of access elsewhere**, with or without limitations, please do so. For example:<br><br>*Data cannot be shared publicly because of [XXX]. Data are available from the XXX Institutional Data Access / Ethics Committee (contact via XXX) for researchers who meet the criteria for access to confidential data.*<br><br>*The data underlying the results presented in the study are available from (include the name of the third party*	Data cannot be shared publicly because the study samples have signed the Privacy Policy during our study. The Privacy Policy precludes us from copying or spreading the original data.<br><br>Centre for Tuberculosis Control of Guangdong Province imposed the Privacy Policy that the study samples signed.<br>Contact Email: bambdd@126.com<br>Address: Centre for Tuberculosis Control of Guangdong Province, Guangzhou, 510630, China<br><br>Shenzhen City Baoan District Chronic Disease Prevention and Treatment Hospital will be available to field future data requests.<br>Contact Email: mby@baoan.gov.cn<br>Address: Shenzhen City Baoan District Chronic Disease Prevention and Treatment Hospital, Shenzhen 518101, China

and contact information or URL).<br>• This text is appropriate if the data are owned by a third party and authors do not have permission to share the data.<br><br>* typeset	
Additional data availability information:	

中山大學 公共卫生学院
School of Public Health, Sun Yat-Sen University

74 Zhongshan Road 2, Guangzhou 510080 P.R. China Tel:(8620)87330678 Fax:(8620)87335498

Dear editor,

We are researchers from Sun Yat-sen University in China. We want to submit an original research article entitled “Analysis on the economic burden of diagnosis and treatment of tuberculosis(TB) patients in the TB control demonstration area of China”, which we wish to be considered for publication in *PLOS ONE*. We believe that several aspects of this manuscript will make it interesting to general readers of your journal.

First, unlike other previous studies, the study is representative of most tuberculosis patients of Shenzhen City, the south-east of China, because about 50% of all cases under DOTS came from the study site. Moreover, since most of the patients enrolled into the study were under DOTS programme regularly and be investigated with strict processes, the results can mirror the economic burden faced by most TB patients in China faithfully. Second, our study suggests that although free TB control policy is implemented, TB patients are still facing a heavy economic burden. More available interventions to reduce finical burden for tuberculosis patients are urgent. Above all, the results of the study can provide a good reference for other developing countries in health policy making and promote public health, especially when facing the risk of TB resurgence in the fast-growing world. This study will be meaningful and fits the aim and scope of *PLOS ONE* well.

We confirm that neither the manuscript nor any parts of its content are currently under consideration or published in another journal. We would be very happy if the manuscript will be evaluated by your Editorial Board members for publication in *PLOS ONE*.

Best regards,

Yixiang Huang MD, PhD
Associate Professor & Vice Chair
Department of Health Policy & Management
School of Public Health, Sun Yat-sen University
74 Zhongshan 2nd Road, Guangzhou, Guangdong, P.R.China, 510080
Email: huangyx@mail.sysu.edu.cn
Tel: +8620 8733 3239

Suggested academic editors:

1.
Helen Suzanne Cox
University of Cape Town Institute of Infectious Disease and Molecular Medicine
SOUTH AFRICA

2.
Erik Schouten
Health Programs Group
MALAWI

3.
Hemant Deepak Shewade
International Union Against Tuberculosis and Lung Disease
INDIA

4.
Lei Gao
Chinese Academy of Medical Sciences and Peking Union Medical College
CHINA

Analysis of the Economic Burden of Diagnosis and Treatment on Patients with non-drug resistance Tuberculosis (TB) in the TB Control Demonstration Area of China

Yixiang Huang[1,#], Jianying Huang[2,#], Xiaoting Su[1], Liang Chen[3], Jianwei Guo[1], Weiqing Chen[4,*] and Lingling Zhang[5]

[1] Department of Health Policy and Management, School of Public Health, Sun Yat-sen University, Guangzhou, 510080, China
[2] Guangdong Women and Children Hospital, Guangzhou, 511442, China
[3] Centre for Tuberculosis Control of Guangdong Province, Guangzhou, 510630, China
[4] Department of Medical Statistics and Epidemiology, School of Public Health, Sun Yat-sen University, Guangzhou, 510080, China
[5] Department of Nursing, College of Nursing and Health Sciences, University of Massachusetts, Boston 02125-3393, USA
*Correspondence: chenwq @mail.sysu.edu.cn
# Yixiang Huang and Jianying Huang contributed equally to this work.

Abstract: Background: Illness-related costs experienced by patients produce a severe economic impact on households, especially those of poor families. Few studies have investigated the full costs, including direct and opportunity costs, at the patient and household levels in the south-east of China. Methods: A case follow-up study was conducted in the Bao'an district of Shenzhen City, China. Eligible new and re-treated individuals with pulmonary TB during January 1st 2013 to June 30th 2013 were enrolled. Medical and non-medical costs as well as income loss were calculated in pre-diagnosis and post-diagnosis periods, respectively. Factors associated pre-diagnosis costs and post-diagnosis costs were explored respectively with a linear regression model. Results: Of the total 514 TB patients enrolled, most were from the migrant population, and 65% were males (336), with a mean age of 32.25 (±10.11) years. The median pre-diagnosis direct costs, direct post-diagnosis costs, and post-diagnosis medical and non-medical costs were 79USD, 748USD, 542USD, and 150USD (6.2897CNY = 1USD, 2013) per patient, respectively. The median total cost of TB treatment per patient was 1218USD, corresponding to 26% of patients' annual income pre-illness. Variables associated with pre-diagnosis costs included number of times visiting health facilities, whether in hospital due to TB, education, History of TB. Variables associated with post-diagnosis costs were household registration, sputum smear status, reported-household economic burden, marital status. Conclusions: Although a free TB control policy is in force, patients with TB are still facing a heavy economic burden. More available interventions to reduce the financial burden on tuberculosis patients are urgently needed.

Keywords: Tuberculosis; cost of illness; economic burden; China

---

1. Introduction

Tuberculosis (TB) is one of the top 10 causes of death worldwide as well as being the leading cause from a single infectious agent (more than HIV/AIDS). According to 2018 global estimates, there were almost 10 million new cases of TB and 1.3 million TB deaths in 2017 [1]. The high prevalence of TB is always a critical public health problem in China, accounting for the second highest number of cases in the world except in India, with 778,000 identified cases of TB in 2017 [1]. Since 1990, the WHO-recommended directly observed treatment + short course chemotherapy (DOTS) strategy to control this epidemic has been implemented by the Chinese government under the National Tuberculosis Control Program [2], whose guidelines include free anti-TB drugs for 6 months to new patients with TB and for 8 months to re-treatment patients in addition to multi-drug resistant/ extensively-drug resistant (MDR/EXDR) patients. Tests include one free X-ray test for individuals suspected of having TB at their first visit, one free sputum smear test for those whose X-ray tests are abnormal and free sputum smear tests for patients undergoing free anti-TB treatment. With the development of molecular biology, a new testing method called GeneXpert/RIF (abbreviated as Xpert) is recommended. With a shorter time of testing, higher sensitivity, and higher specificity, Xpert has been rapidly adopted for clinical diagnoses in China [3].

However, many investigators have pointed out that both the TB patients and their households bear a heavy economic burden. Even the free DOTS control strategy is far from sufficient [4, 5]. Tuberculosis is a well-known poverty-related disease, an association that has been well-established at both patient and household levels, usually related to the disadvantages of socio-economic status. In particular, among those who come from rural and poor districts, the scarcity of health resources and their poor economic condition place them in

a more serious situation [6]. The financial aspect is a major factor contributing to health-care-seeking behavior among Chinese patients. Delayed and repeated visits to clinics before diagnosis, the over-prescribing of drugs, and prolonged treatment are common [7]. Patients must bear too much economic burden, such as, for example, medical and transportation costs, and income loss, precluding them from further adhering to treatment and resulting in treatment failure [5].

There have been many studies on the cost-effectiveness of different TB control strategies, mainly investigating the costs to the health system in the Eastern, Middle, and Western Regions of China. These studies focused mainly on the effectiveness of medical insurance for coping with the costs of illness as well as patient compliance with drug regimens [5, 8]. However, far fewer studies have measured the costs to patients and households or the full costs, including direct and opportunity costs, from the onset of symptoms to the end of treatment in the south-east of China. To achieve the health-related Sustainable Development Goal (SDG) proposed by the United Nations and the goal of ending the TB epidemic by 2035 proposed by WHO, whose last target reads "No TB patients and their families face Catastrophic costs (>20% of annual household income)" [9], all TB patients should receive accessible and affordable standard medical treatment. Therefore, the present study was performed to evaluate the out-of-pocket direct costs as well as productivity lost related to illness. Potential factors associated with these costs were also explored. We aimed to establish the evidence base for interventions that can contribute to TB-related cost reduction, increase patient adherence to treatment, and treatment success rates. There is a need to document the full economic burden of TB care borne by patients and their households.

2. Materials and Methods

2.1. Study Setting

Shenzhen City is one of the most prosperous regions in China, with a Gross Domestic Product (GDP) of nearly 269 million CNY in 2019. Its estimated population is over 13 million, with the migrant population constituting 65.3% while this rate is 82.4% in Bao'an district of the total regional population according to the Shenzhen statistical yearbook 2019. The mean household income in the region was 6,656.79 USD of 2012 according to the Statistical yearbook 2013. The rapid economic growth in the city, leading to higher income levels, attracts numerous young migrant workers, mostly from remote poor areas exposed to substandard conditions such as lower societal status, poor standard of living, limited health knowledge, and less accessibility to health services, thus creating favorable conditions for infection with Mycobacterium tuberculosis and progression to disease. Such conditions delay individuals from seeking health care, resulting in advanced disease and continued transmission of TB in the community. Although analysis of recent data has shown a decreasing incidence of TB in the Shenzhen region, its prevalence in the Bao'an district, where a heavy concentration of migrant workers contributes to a high TB case load, is still high.

The Bao'an district implemented the 10-year World Bank TB project in 1992, brought the migrant population into the National TB control program (NTP), and adapted the DOTS strategies. After this ended in 2002, the Shenzhen TB control project in Guangdong province was launched. Then, as a representative of Guangdong province, the Bao'an district was selected as one of the seven counties to implement the fifth round of the globally funded TB control project. Up to 2010, the Bao'an district was selected by the China Ministry of Health as the site of a demonstration area for a tuberculosis control model. The hospitals were the main place finding tuberculosis case and the Center for Prevention and Cure of chronic diseases (CPC) were the local designated TB facilities in the national TB program networks, providing treatment and managing the tuberculosis cases. The healthcare providers in general hospitals were obligate to refer the suspected cases to CPC and the basic information of suspected cases were sent to CPC. If the cases did not consult to CPC in time, the workers of CPC would contact the suspected

case. Actively cooperating with medical institutions and TB prevention and control institutions, the discovery rate and report quality was increasing by years. In 2010, the prevalence rate of smear positive of native population was 19.44 per 100 000 population, and that of the migrant population was 59.96 per 100 000 population and the cure rates for both native and migrant patients were 94.79% and 82.91%, respectively [10].

The main health-care sources in China are district public health facilities organized into three tiers of referral: primary (Center for Prevention and Cure of chronic diseases), secondary (sub-district prevention and care dispensaries), and tertiary (Community health-care stations) [11]. The DOTS program means directly observed treatment +short course chemotherapy for non-inpatient tuberculosis patients, and is available only in public health facilities, where patients have free access to diagnostic and treatment services. The Center for Prevention and Cure of chronic diseases (CPC) is the institution authorized to provide TB diagnosis, treatment, and monitoring. The CPC provides patients with a radiologic imaging studies (X-ray) while diagnosed, and sputum smear tests during treatment, and anti-TB drugs for 6-8 months for free. Patients must regularly return to CPC every month for health checks after starting to take anti-TB drugs until the treatment is over. Generally, the numbers of times of subsequent consultations are 5 and 7 to newly-patents and re-treated patients respectively. The costs of every month including auxiliary examinations and subsidiary drugs like hepatinica, gastric medicine, and immunopotentiator etc. are not free. The sub-district prevention and care dispensaries and Community health-care stations coordinating with the CPC are obligated to perform routine monitoring, referral, and supervision. Adherence is directly monitored by the health workers of Community health-care stations for daily drug intake, adverse reactions, and other activities of admitted patients. If patients fail to take a specific drug for 2 days, the workers will contact them by telephone to ensure that the patients complete their treatment.

2.2. Study Design and population

A case follow-up study was conducted in the Bao'an district with an ongoing NTP-DOTS project. Newly diagnosed and re-treated TB patients aged 15-59 years old, who registered in the CPC during 1 January 2013 to June 2013, were included and were followed up until the completion of a 6- to 8-month treatment course. Those with severe co-morbidities such as AIDS, diabetes, and lung cancer and those who refused to accept investigation were excluded. The patients aged above 59 were excluded considering the elderly may have more comorbidities and those with MDR-TB were excluded too. Transferred patients were also excluded from the study, since information on previous costs was not available. The operational definition of various costs and indicators were summarized in table 1.

2.3. Data Collection

All eligible cases were subjected to five (new patients) or seven (re-treatment patients) interviews at the Department of Tuberculosis Prevention and Control in Bao'an CPC. A structured questionnaire was used, covering general demographic and socio-economic characteristics, disease history, care-seeking process, treatment behaviors, and costs of pre- and post-diagnosis. Self-reported economic status information was collected based on different localities with various levels of development. The first interview was at the first month of intensive phase of treatment, when patients' retrospective care-seeking history and related expenditures, including pre-diagnosis, starting from the time point when the patients experienced the first symptoms, and the intensive phase related costs, were collected. The other four or six interviews occurred monthly when patients came for regular re-examinations. The patients' TB care experiences and transportation fee, supplementary food cost, and foregone income of patients and companions were collected in follow-ups.

A graduate student with two locally recruited research assistants, health workers who had participated in a standardized training session, conducted those interviews. A logic check of all

data collected was undertaken to determine if there were any contradictions or missing information. Those contradictions or missing information would be asked of patients again in a subsequent interview. We also evaluated the quality of collected data by verifying the routine health information system.

2.4. Measurements and Definitions

We analyzed the economic burden from tuberculosis patients' perspective. The primary outcome variables were the mean (median) expense incurred during illness. Information on costs was ascertained for different periods, summarized as pre- and post-diagnosis.

Direct costs included all costs of patients and companions attributable to their illness [12], which consisted of the medical costs related to TB diagnosis and treatment (clinical and hospitalization expenses), and non-medical costs incurred by patients or companions, such as transportation to health facilities and supplementary food. Opportunity costs referred to the income lost by patients and their companions associated with time lost off work. Assuming 30 working days per month, we evaluated the value in terms of money for each day, and opportunity costs was calculated as the value per day multiplied by the length of time off work due to illness. The income lost before diagnosis was calculated based on each patient's monthly wage prior to the onset of TB. The lost income after diagnosis was calculated based on the monthly wage if patients were still on working during illness. After diagnosis, patients were asked about the actual lost income that they and their companions had experienced due to absence from their usual income-generating activities at every interview. These monthly income reductions were then summed. The income lost by companions pre-diagnosis was not included in the analysis, since only a few such losses occurred. The time spent on daily drug intake was also not calculated, because most patients took drugs on their way to work or at the community health-care stations close to their homes.

The economic burden was then analyzed as a proportion of patients' total annual income. The free services provided during diagnosis and treatments were not included in the medical costs. Information about costs after diagnosis was extracted from the information system of the study facility. Information on costs covered by insurance could not be obtained, since the insurance had not been settled at the end of the interview.

Table 1. Operational definition of study participants and TB treatment cost in the study of Bao'an district, Shenzhen City, China, 2013

Terminology	Definition
Study participants	All newly diagnosed and re-treated TB patients accepted treatment in CPC during 1 January 2013 to June 2013
Study participants ineligible	Patients with severe co-morbidities such as AIDS, diabetes and lung cancer with multidrug-resistant TB, aged>59 were excluded.
Pre-diagnosis costs	Costs between symptom onset and diagnosed as TB
Post-diagnosis costs	Costs from treatment initiation up to treatment completion
Direct medical costs	Expenses of medical examinations and medicines linked to TB diagnosis and treatment
Direct non-medical costs	Costs for transport, food expenditures ,nutrition supplements due to TB
Direct cost	Direct medical costs + direct non-medical costs
Opportunity costs	Patients and companions' lost income due to TB-related time off work during the TP episode. The pre-diagnosis opportunity cost was estimated using the time off work multiplied by the reported individual income prior to the onset of TB

Total costs	Direct costs + opportunity costs
Sputum smear test	At the time of diagnosing conducted one sputum smear test, the outcome included negative and positive.
Household economic burden	Self-reported economic burden due to TB treatment
The degree of symptoms at diagnosis	The patients self-reported with different symptoms at diagnosis were divided into four categories. 1. None: no symptoms; 2.mild: with symptoms included cough, expectoration and dyspnea, night sweat, and debilitation; 3. Moderate: symptoms with fever and chest distress; 4. severe: symptoms like hemoptysis.
Interruption of treatment	Treatment interruption is defined as any interruption of treatment for at least one day but for < 8 consecutive weeks

2.5. Data Analysis

The data were double-entered with EpiData 3.1 software (EpiData Association, Odense, Denmark) for each patient, and cost summaries and analyses were performed by SPSS 20.0 software. Continuous variables like direct costs, opportunity costs were summarized as means (± standard deviation [SD]) or medians (IQR), while categorical variables were summarized as proportions. Group comparisons were made with the *rank correlation* for median, the Mann-Whitney test or Kruskal-Wallis test for medians in two or multiple groups if their distribution were non-normal. We also did a confounder adjusted analysis for the association between pre-diagnosis costs or post-diagnosis costs and predictor variables respectively using linear regression. The costs were log transformed as they were not normally distributed. To explore more variables as predictor variables, the possible variables were all considered in the linear regression model irrespective the value in univariate group comparisons. A forward stepwise approach was used to find the appropriate model. Association was summarized using Beta coefficient (95% CI), *P*-value less than 0.05 were significant. Considering the multicollinearity in predictor variables, we calculated variance inflation factor (VIF) at the same time [13], If VIF was less than 10, then the predictor variables were included in the linear model.

Considering in applying and interpreting the results of hypothesis testing in a log-transformed data on actual data (non-log transformed) [14]. Hence, we also conducted a confounder-adjusted association between costs and predictor variables using generalized linear model (logistic regression, the costs were divided as binomial variables based on median value. To explore more variables as predictor variables, the possible variables were all considered in the linear regression model irrespective the value in univariate group comparisons. Association was summarized using exp(β) coefficient (95% CI), P-value less than 0.05 were significant.

2.6. Ethics Approval

This study was approved by the research and ethics committee of the School of Public Health, Sun Yat-Sen University, China. Also, permission was obtained from the study site. A signed informed consent was obtained from all patients before being interviewed.

3. Results

3.1. Socio-demographic Characteristics and Care-seeking Behaviors

The present study surveyed 533 eligible pulmonary tuberculosis patients who with no co-morbidity, 19 of whom were lost to follow-up, one of whom died from another disease, and 18 of whom moved to other provinces. The rate of loss to follow-up was nearly 4%. Among the

514 patients receiving anti-TB treatment, 30 were re-treatment patients, and 95% were members of the migrant population from all over the country. The average age was 32.25 ± 10.11 years, 65% (336/514) of the patients were male, and 35% were female. The mean monthly income of patients before illness was 448USD, about 71.4% of patients had monthly household income between 317.98 to 635.96 USD. One hundred seventy-six patients were identified when they accepted health check-ups required by their work units, and were then transferred to the CPC. They were diagnosed at CPC immediately without consulting to other facilities, so their pre-diagnosis costs were not calculated. Three hundred thirty-eight patients were registered as a result of clinical consultation. Among the 338 patients, the median delay time of visiting doctors, defined as the period between onset of symptoms and first contact with a health-care provider, was 11 days (IQR 3-30), for reasons such as having no time, being unaware of the presence of tuberculosis, and lacking money. The median number of consultations prior to diagnosis was 3 (1, 20). The number of first visit in community healthcare center and general hospital was 71(21%) and 136(40.2) respectively. Only 48 (14%) patients initially visited a public facility, while the others reported that they first consulted either a drug shop or private clinic or did nothing. Then median delay time of diagnosis was about 7 (0,200) days.

3.2. *Pre-diagnosis Direct Costs*

Among the 338 patients who incurred costs due to consultation, the median outpatient medical cost incurred pre-diagnosis was 64USD (IQR 24-159) per patient. Twenty-two patients were admitted to the hospital before TB was diagnosed. Hospital patients incurred a substantial higher out-of-pocket expenditure. The median hospital expenditure was 477USD (IQR 318-823). The non-medical costs refer to transportation and accommodation fees. This study surveyed only the transportation fees for patients before diagnosis. The median transportation fee was 2USD (IQR 0-8). Costs due to the prescribing of non-TB drugs and diagnosis costs accounted for a high proportion of direct expenditures in the pre-diagnosis period (97.0%).

3.3. *Post-diagnosis Direct Costs*

A patient's average number of health-care visits after TB was diagnosed was 9.7, with a maximum of 15 visits. The direct costs after TB was diagnosed were 748USD (IQR 567-987); the average cost for one visit was 77USD. Median medical cost was 542USD (IQR 465-618), then we further analyzed what constituted medical costs. Median costs for examinations and non-TB drugs were 307USD (IQR 273-341) and 211USD (IQR 166-265), respectively, which were clearly the most robust components, accounting for 55% and 40% of medical costs, respectively. Meanwhile, the auxiliary drugs, such as liver/kidney protection drugs and immunopotentiators, constituted the main part of non-TB drugs, with a median cost of 174USD (IQR 135-219), accounting for 83% of the non-TB drug costs.

The non-medical costs in the post-diagnosis period included transportation generated by patients and companions and supplementary food for patients. The median transportation cost was 19USD (IQR 10-31); the average cost for a single event was 3USD. Among the surveyed patients, 196 were escorted during treatment by their family or friends, and the median transportation cost was 5USD (IQR 2-13). About 340 patients improved nutrition with supplementary food, and the median cost was 302USD (IQR 127-489). The non- medical costs totaled 150USD (IQR 29-410). Clearly, the non-medical costs were relatively low (Table 2).The direct costs of intensive phase (the first two month) and consolidation phase(the next 4 or 6 month) 210.58(164.87,279.66) USD and 516.48(391.91,703.39) USD respectively. The direct costs of consolidation phase accounted for 70.7% of total pre-diagnosis direct costs.

3.4. *Opportunity Costs*

Only 49 patients reported income lost while away from work due to seeking health care before diagnosis. Income lost by those patients, incurred during the pre-diagnosis period up to standard anti-TB treatment, was 32USD (IQR 16-83). Most of the opportunity costs were from lost income after TB was diagnosed (Table 2), because most patients were fired or could not physically continue working. For the pre-diagnosis period, the income loss was calculated based on each patient's daily income before illness (median, 32USD), multiplied by the total time the patient was out of work due to illness (median, 2 days, $n = 49$), mainly due to travel and consultation time at different health-care facilities. For the post-diagnosis period, the monthly income reduction for a complete treatment cycle was summed. The income lost at two stage of treatment was 31.80(0, 63.60) USD and 127.19(38.16, 683.66) USD. Most income lost came from the consolation phase. The opportunity cost during consolidation period accounted for 79% of total post-diagnosis opportunity cost. The time lost due to daily drug intake was not calculated in the current study.

3.5. Total Patient and Household Costs

The sum of median direct and opportunity patient and household costs was 1218USD (IQR 826-1963), which corresponds to 26% of annual individual incomes before TB illness (4770USD). The direct costs accounted for 61% of the total costs, and the ratio of direct to opportunity costs was 1.58. About 88.2% (1074/1218) of costs were encountered after the patients were diagnosed and accepting free DOTS treatment.

Table 2. Median direct and opportunity costs of TB patients during different episodes of treatment in the study of Bao'an district, Shenzhen City, China, 2013

Timing and Types of Costs	Patient-reported costs during this Period			All Patients***
	Number*	%	Median** (IQR range)	($n = 514$)
**Pre-diagnosis**				
**Direct costs**				
Outpatient medical costs	338	66	64 (24-159)	19 (0-79)
Hospital costs	22	4	477 (318-823)	0 (0-0)
Transportation costs	338	66	2 (0-8)	0 (0-3)
**Pre-diagnosis direct costs^**				**79 (24-191)**
**Opportunity costs**				
Foregone income before diagnosis	49	10	32 (16-83)	0 (0-0)
**Median cost of pre-diagnosis[d]**				**79 (25-204)**
**Post-diagnosis**				
**Direct costs**				
**Medical costs**	**514**	**100**	**542 (465-618)**	**542 (465-618)**
Examination	514	100	307 (273-341)	307 (273-341)
Non-TB drugs	514	100	211 (166-265)	211 (166-265)
Auxiliary drugs	514	100	174 (135-219)	174 (135-219)
Others	514	100	21 (20-24)	21 (20-24)
**Transportation**				
For patients	514	100	19 (10-31)	19 (10-31)
For companions	196	38	5 (2-13)	0 (0-3)

**Supplementary food**	340	66	302 (127-489)	127 (0-376)
**Post-diagnosis direct costs**				**748 (567-987)**
**Opportunity costs**				
Foregone income of patients	402	78	241 (90-1,181)	114 (24-797)
Foregone income of companions	196	38	39 (16-84)	0 (0-24)
**Median cost of post-diagnosis**				**1074 (745-1820)**
**Total direct cost**				**833 (609-1132)**
**Total opportunity cost**				**157 (57-855)**
**Median cost of all periods**				**1218 (826-1963)**

*Total number of patients, 514.

**The average cost among patients who generated this kind of costs in row 2.

**The average cost for all patients (514).

^Pre-diagnosis direct cost = direct costs + transportation costs. Median cost of pre-diagnosis = direct costs + opportunity costs.

3.6. The association of pre-diagnosis costs among tuberculosis patients

The socio-demographic and care-seeking level characteristics of pre-diagnosis costs were summarized in table 3. Those re-treated patients had more costs compared with newly patients (P=0.05) and patients who were in hospital due to tuberculosis generated more costs (P<0.0001). It seemed that the more number of times visiting to health-care facilities (P=0.05)，the longer of delay of diagnosis(P<0.0001) and delay of first visiting health-care facilities when symptom onset.

The direction and significance of association between the pre-diagnosis costs and the number of times visiting to health-care facilities, whether in hospitals due to tuberculosis before diagnosis did not changed after a confounder adjusted analysis in linear regression analysis . However, the increase in delay of diagnosis was not found to be statistically significant in adjusting analysis (Table 4). But we found those who received higher education or occupied in national civil servant/services /retired staff might expense more before diagnosis in the linear regression analysis too. In the corresponding generalized linear regression, number of times visiting health-care facilities was found to be related pre-diagnosis costs statically significant (P<0.0001). (Table 5)

3.7 *The association of post-diagnosis costs among tuberculosis patients*

The socio-demographic characteristics of post-diagnosis costs had been depicted in Table 6. Migrant population might have more costs during the tuberculosis treatment compared with native population (p=0.003). There were difference of costs after diagnosis as TB among different education level patients (P=0.039). Those who perceived that the TB imposed economic burden to household might have more expenses on the treatment (P=0.021).The status of sputum smear test at the time of diagnosis was positive generate more costs compared to the negative (P<0.0001).

After a confounder adjusted analysis, the direction and significance of association of post-diagnosis costs and the household registration (migrant or native residents), reported

economic burden of households, sputum smear status did not changed (Table 7). The divorced or widowed patients had more costs compared to the unmarried in the linear regression analysis (p=0.044). In the corresponding generalized linear regression, only sputum smear status adopted in the model. (P<0.0001). (Table 8)

Table 3. Association of pre-diagnosis costs by background characteristics and care-seeking behaviors among tuberculosis patients enrolled in the study of Bao'an district, Shenzhen City, China, 2013

variables	Number	%	median(IQR range) of pre-diagnosis cost	*P*
**Gender**				
Female	116	34.32	68.37(18.28,166.14)	0.121
Male	222	65.68	81.48(31.8,240.39)	
**age**	338	100.00	79.5(25.2,204.43)	0.723
**Household registration**				
Native patients	21	6.21	34.98(23.85,158.99)	0.15
Migrant patients	317	93.79	79.5(25.44,205.63)	
**Marital status**				
unmarried	105	31.07	79.5(23.85,238.49)	0.158
married	217	64.20	79.5(25.44,190.79)	
widowed/divorced	7	2.07	79.5(58.03,203.67)	
others	9	2.66	50.88(19.71,317.98)	
**Education**				
Primary/illiterate	48	14.20	72.5(17.01,168.53)	0.171
Junior high school	139	41.12	66.78(23.85,164.55)	
Senior high school	110	32.54	79.5(31.8,182.84)	
college or above	41	12.13	174.89(47.7,386.88)	
**Occupation**				
Workers	203	60.06	78.7(23.85,222.59)	0.401
Individual business	37	10.95	66.78(23.85,164.55)	
Others (national civil servant/services /retired staff etc.)	64	18.93	123.22(57.63,328.31)	
Unemployed	34	10.06	174.89(47.7,386.88)	
**Number of times visiting health-care facilities**				
<=2	214	63.31	34.18(15.9,95.39)	<0.0001
3~6	101	29.88	158.99(81.08,289.36)	
>=7	23	6.80	724.52(328.79,724.52)	

**health-care facilities for the first time**				
community healthcare center	71	21.01	100.69(37.36,283)	0.05
public facility	48	14.20	95.39(63.6,249.61)	
general hospital	136	40.24	48.02(15.9,174.89)	
private clinics	38	11.24	57.55(23.85,127.19)	
others	45	13.31	87.44(31.8,174.89)	
**delay between symptom onset and first consultation**				
<=7	152	44.97	80.29(28.14,271.48)	0.375
7~14	38	11.24	73.93(31.8,174.89)	
14~21	42	12.43	64.39(17.49,119.24)	
>=21	106	31.36	79.5(19.71,174.89)	
delay of diagnosis				
<=7	186	55.03	32.91(15.9,96.67)	<0.0001
7~14	35	10.36	79.5(37.92,167.73)	
>14	117	34.62	166.94(81.08,479.83)	
**Whether in the hospital due to TB**				
yes	22	6.51	1203.28(675.71,1564.46)	<0.0001
no	316	93.49	71.55(23.85,1669.55)	
**The degree of symptoms at diagnosis**				
None	50	14.79	76.63(17.49,280.88)	0.412
Mild	112	33.14	69.96(23.85,172.9)	
Moderate	88	26.04	80.61(31.8,173.94)	
Severe	88	26.04	82.2(31.8,302.08)	
**History of TB**				
New patients	315	93.20	78.7(23.85,190.79)	0.05
Re-treated patients	23	6.80	100.69(48.49,297.98)	

Table 4 Confounder adjusted association between *pre-diagnosis costs* and various predicator variables using linear regression models in the study of Bao'an district, Shenzhen City, China, 2013 (n=338)

Predictors in the model	Beta coefficient	(95% CI)	P value	VIF
**Number of times visiting health-care facilities**	0.12	(0.11,0.14)	< 0.001	1.05
**whether in hospital due to TB (ref: no)**				

yes	-1.02	(-1.2,-0.84)	< 0.001	1.03
**education（ref:Primary/illiterate)**				
college or above	0.26	(0.12,0.39)	< 0.001	1.03
**Occupation(ref:workers)**				
Others (national civil servant/services /retired staff etc.)	0.13	(0.02,0.24)	0.02	1.03
**History of TB (ref: new patients)**				
**Re-treated patients**	0.22	(0.04,0.40)	0.02	1.02

Table 5 Confounder adjusted association between *pre-diagnosis* costs* and various predicator variables using logistic regression models in the study of Bao'an district, Shenzhen City, China, 2013 (n=338)

predictor in the model	B	Exp(B)	(95% CI)	P value
**Number of times visiting health-care facilities**	**0.818**	**2.267**	（1.760,2.919）	**<0.001**
**History of TB (ref: new patients)**				
Re-treated patients	1.001	2.721	（1.037,7.140）	0.042
**Education(ref:Primary/illiterate)**				
Junior high school	-1.760	0.172	（0.062,0.475）	0.001
Senior high school	-1.168	0.311	（0.137,0.705）	0.005
college or above	-.901	0.406	（0.176,0.936）	0.034
**delay of diagnosis**	-.014	0.986	（0.971,1.001）	0.062
**constant**	-1.920	0.147		0.003

*Pre-diagnosis costs in generalized linear model (logistic regression) was categorized based on median value among all participants

Table 6 Association of post-diagnosis costs by background characteristics and care-seeking behaviors among tuberculosis patients enrolled in the study of Bao'an district, Shenzhen City, China, 2013

Variables	Number	%	median(IQR range) of pre-diagnosis cost	p

**Gender**				
Female	178	34.63%	1128.91(764.9,1717.51)	0.309
Male	336	65.37%	1128.91(764.9,1717.52)	
Age	514	100%	1074.38(745.35,1818.69)	0.177
**Household registration**				
Native patients	26	5.06%	760.93(489.53,1277.49)	0.003
Migrant patients	488	94.94%	1096.24(757.65,1852.85)	
**Marital status**				
unmarried	180	35.02%	1103.47(743.68,1864.68)	0.193
married	308	59.92%	1018.08(746.43,1719.76)	
widowed/divorced	10	1.95%	2208.69(844.63,3183.62)	
others	16	3.11%	879.98(691.18,1803.74)	
**Education**				
Primary/illiterate	63	12.26%	859.61(662.77,1398.92)	0.039
Junior high school	220	42.80%	1150.77(827.62,1921.22)	
Senior high school	169	32.88%	1021.42(722.61,1632.99)	
college or above	62	12.06%	1064.25(682.54,1924.26)	
**Occupation**				
Workers	203	60.06%	78.7(23.85,222.59)	0.772
Individual business	37	10.95%	66.78(23.85,164.55)	
Others (national civil servant/services /retired staff etc.)	64	18.93%	123.22(57.63,328.31)	
Unemployed	34	10.06%	174.89(47.7,386.88)	
**Sputum smear status**				
negative	287	55.84%	963.15(699.21,1601.36)	<0.0001
positive	227	44.16%	1222.47(803.39,1982.94)	
**Interruption of treatment**				
Yes	67	13.04%	1084.95(801.68,1412.53)	0.572
No	447	86.96%	1067.95(740,1864.68)	
**Household economic burden**				
Heavy	149	28.99%	1153.54(787.48,1976.25)	0.021
Moderate	236	45.91%	1096.28(740.46,1888.75)	
No burden	129	25.10%	961.76(668.92,1344.58)	
**Adverse drug reaction**				

Yes	268	52.14%	1112.96(743.44,1852.95)	0.447
No	246	47.86%	1009.81(745.35,1792.01)	
**The degree of symptoms at diagnosis**				
None	131	25.49%	1009.75(733.98,1810.5)	0.737
Mild	181	35.21%	1014.74(719.11,1654.26)	
Moderate	103	20.04%	1143.66(770.15,1728.62)	
Severe	99	19.26%	1140.12(757.65,1971.92)	
**History of TB**				
New patients	484	94.16%	1043.25(733.95,1817)	0.064
Re-treated patients	30	5.84%	1293.75(956.37,1911.22)	

Table 7 Confounder adjusted association between *post-diagnosis costs* and various predicator variables using linear regression models in the study of Bao'an district, Shenzhen City, China, 2013 (n=514)

Predictors in the model	Beta coefficient	(95% CI)	P value	VIF
**Sputum smear status（ref：negative）**				
positive	0.08	(0.036,0.128)	< 0.001	1.005
**household registration (ref: Native patients)**				
Heavy	0.15	(0.043-0.254)	0.006	1.032
**reported-household economic burden（ref:no burden)**				
Heavy	-0.06	(-0.109,-0.003	0.040	1.038
**marital status (ref:unmarried)**				
widowed/divorced	0.17	(0.04,0.333)	0.044	1.03

Table 8 Confounder adjusted association between *post-diagnosis* costs* and various predicator variables using logistic regression models in the study of Bao'an district, Shenzhen City, China, 2013 (n=514)

predictor in the model	B	Exp(B)	(95% CI)	P value
**Sputum smear status (ref: negative)**				
positive	0.653	1.921	（1.350,2.735）	<0.001
**constant**	-0.288	0.750		0.016

4. Discussion

This study evaluated the economic burden borne by TB patients in Shenzhen City during their illness. We included 533 eligible patients in the survey, 19 of which were lost to follow-up. The average age of them was 29.6±10.2years. The male was 14 and female was 5. One patient was with a severe symptoms appeared hemoptysis. The others were with mild or moderate symptoms like cough, chest distress and fever etc. the lost to follow-up was about 4 percent, and the general Socio-demographic and clinical characteristics of those 19 participants lost to follow-up were identical with the 514 patients. So，we considered there no much influence on our conclusion.

As one of the most population of Shenzhen district and owing numbers of factories, the migrant population in Bao'an always exceeds the average level of Shenzhen city. The total number of registered active tuberculosis cases were 1317, included 76(5.8%) native case and 1241(94.2%) migrant cases in 2013[15]. In our study, 95 percent were migrant cases, and about 80% of patients were factory workers or service staffs who general were in a poor economic status and poor living environment. Under the DOTS program, the CPC provide tuberculosis patients with a radiologic imaging studies (X-ray) , sputum smear tests and anti-TB drug during treatment for free amounting to 102.07USD. We found that the migrant patients faced much costs during TB treatment. This may be owing to the different mode of payment and free strategy. The current study confirmed that TB patients are from vulnerable groups, and that migrant groups working in factories face worse living conditions and lower average income than the general population.

The median household costs found in this study (1218USD) were far higher than those in Africa, India, and rural China [16-18]. These differences could partly be explained by the differences in purchasing power among these countries/districts and/or due to the omission of some cost elements in these studies. The more important reason may be the prospective follow-up design of this study, which reduced recall bias. We found that, one year prior to the study, 78.5% percent of patients had annual household incomes between 795 and 15,899USD, while 13 patients made less than 795USD a year. The direct and opportunity costs corresponded to 26% (1218/4685) of annual personal income. These costs constituted a considerable part of their disposable income after payment of fixed costs such as rent, electricity, and water, along with insurance. Moreover, patients must face these costs for more than 6 months; over a quarter of patients reported that the economic burden of cure was heavy. Twenty-three patients reported incurring debts due to illness. Factory workers and the unemployed had to rely on help from their families. Some authors have concluded that all costs to TB patients account for a higher percentage of their annual income [16, 19-21]. For example, a survey in three countries showed that treatment-related costs were equivalent to approximately one year of individual income [22]. The differences between these studies and ours may be explained by social background, and a higher income level in our study population.

Direct costs are still substantial, with non-TB drugs and auxiliary examinations as the most significant cost items [23-25]. Additional drugs, such as those for protecting the liver/kidney or symptomatic treatment, and examinations must be paid for, and it appears that this led to the high costs during the treatment period, indicating that there may be over-prescription and over-service, as reported in other studies [26, 27]. Although income loss and time loss have not affected every tuberculosis patient interviewed, a considerable number of patients reported being negatively influenced by the disease and concomitant income and time loss. About 37% (190/514) of the patients were unemployed or suspended from work after contracting TB and had no income, while the others remained employed and experienced income loss due to monthly doctor visits. Only 36% of patients had insurance through the workplace. Patients reported that only a small fraction of medical costs could be reimbursed, and if they became unemployed, their insurance could lapse. Fifty-nine percent of patients had no insurance. Thus, departure from daily money-earning activities had a

negative economic impact on patients with TB, resulting in impoverishment and worsening their living situations [23]. However, the opportunity costs in these cases may be underestimated, since the value of time loss due to daily drug intake was not calculated, and some patients reported that they had no clear concept of their reduced income due to absences from the workplace.

Out-of-pocket costs and income loss pre-diagnosis were much lower compared with those post-diagnosis, accounting for less than 10% of total costs. This is consistent with reports from other studies [28, 29]. A review of the financial burden of tuberculosis indicated that half of total costs were incurred before treatment [30]. The possible reason for this may be that individuals found it difficult to comprehend the concept of calculating the monetary value of unpaid work, and many of the patients in this study were found as a result of the health check-ups required by their work units. Those suspected patients were initially referred to the CSC for diagnosis, and the first X-ray and sputum smear test were free for them. The delay of diagnosis and number of times visiting health-care facilities were decreased and less costs incurred. We find that those who accepted higher education and occupied in national civil servant/services or retired staff may incur more costs before diagnosis. Those patients may have a good economic condition and care more about self-health conditions.

Less than one-fifth of patients initially visited a public health-care facility at the onset of TB symptoms. This led to long delays in the identification of a treatment-seeking pathway before a proper diagnosis was confirmed. The reasons for this may be gender, education, and economic conditions in terms of patients who missed the perfect treatment period, resulting in an advanced disease [31]. Financial considerations are a major factor contributing to health-seeking behavior in the Chinese population [32]. Inefficiencies in public health facilities and control services at private facilities are the two main constraints responsible for prolonged delay and increased costs of TB diagnosis [33, 34]. In our analysis, we found visiting times as one of the factors affecting related pre-diagnosis costs, with a mean of 3 visits to different health-care facilities. Only 35.6% of patients reported that they were diagnosed with suspected tuberculosis at their first visit, and the rest were informed that they had pneumonia, pleurisy, a cold, and so on. Consistent with our study, other investigators pointed out that early diagnosis of tuberculosis reduced costs while achieving treatment success [35, 36]. Given that TB case detection under the current DOTS strategy in China is through passive case findings, it is imperative to increase patient and community health education and, further, to strengthen the consciousness and capacity of grass-roots public facilities to transfer suspected TB patients to better-equipped hospitals through improved work motivation.

Treatment interruption is defined as any interruption of treatment for at least one day but for < 8 consecutive weeks [37], which has been proven to be a risk factor in treatment failure, drug resistance, relapse, and death [38, 39]. Economic disadvantage is strongly associated with non-adherence to TB treatment [40]. Meanwhile, the link between medical costs and adherence in China has been proven [41]. In our study, we didn’t find that this related to high treatment costs statistically. But we find that above seventy percent of post-diagnosis costs occurred during consolidation period. This may be an economic factor influencing the adherence of treatment. Thirteen percent of patients reported that they had interrupted treatment for more than two months, mainly due to: first, side-effects from drugs, when patients experienced a heavy adverse reaction such as liver damage, especially at the intensive period, which always required doctors to stop treating to protect the liver; second, when patients returned to their home towns, where current strategies prevented them from taking anti-TB drugs beyond one month, so many chose to stop treatment; and finally, more than 90% of the patients who discontinued treatment did so during the continuation phase of treatment, reportedly because, during this phase, most symptoms disappeared and patients erroneously believed that they were cured, making them reluctant to bear the extra burden of the medical costs and income loss. High treatment costs may deter patients who have the most income loss

from continuing treatment [42]. The patients who stopped treatment usually developed advanced disease difficult to cure, resulting in a longer period of cure and, in turn, higher costs. This seems to be a vicious cycle whereby expenditures increase while interruption leads to more expenditure. Other factors, such as inconvenient daily drug intake and work obligations, were also taken into consideration by the patients.

Although gender was associated with higher costs in some studies [43], we found no difference in costs between male and female patients, consistent with results from another study [44]. Re-treated patients and sputum smear positive patients may have higher expenditures during different treatment episodes due to a longer treatment period and more complicated conditions.

This study may seem to be only partly representative of Shenzhen City, since only one district was included. However, the study is representative of most tuberculosis patients in Shenzhen City, because about 50% of all cases under DOTS came from the study site. The re-treatment rate in our study was 5.84%, which is similar to that reported in other studies, with a re-treatment rate of 6.7% in Shenzhen [45] and 6.4% in other provinces in China [46]. This indicates that our study was acceptably representative of the treatment conditions for TB patients in Shenzhen specifically and China generally. As other studies related to the household costs of tuberculosis, this study also relied on self-reported costs of pre-diagnosis and household income. Thus, recall and reporting bias could not be avoided. Hence, we conducted the first interviews of patients after one month of treatment to reduce recall time. Interviewers were also trained to reduce the bias by logic-checking the information from the registration system and recognizing unusually high costs for specific items. In addition, asking patients to calculate the monetary value of unpaid work proved to be difficult, even though our interviewers explained as much as they could.

Our study does have some limitations. First, our study only investigated the economic burden of patients brought by TB and those with severe co-morbidities such as AIDS, diabetes, and lung cancer and older than 59 were excluded. Therefore our study may reflect the economic burden faced by patients result from TB instead of all population and all kinds of diseases. Second, in our study, we did not survey the information regarding companion costs although it may not have much impact on the results since most TB patients in China did not need to be companion during the treatment. Third, we collected the range of the household's pre-TB annual income of every patient instead the exact number. As a result, we are not able to conduct the analysis regarding catastrophic cost.

5. Conclusions

In conclusion, patients and their households face heavy expenditures due to the costs of TB care, which accounted for 26% of patients' annual income. Costs were not equally spread over time, but in the post-diagnosis period, the costs are highest, exacerbating the problem of affordability. Free TB drugs are not the same as free treatment, and non-TB drugs, examinations, and loss of income are important considerations for patients. The income loss among all patients averaged 157USD (IQR 57-855). More visits to health-care facilities generated more out-of-pocket costs. Patients who had severe symptoms and re-treated patients bore a heavier burden. For high costs to be avoided, more attention should be paid to TB patients, even under the DOTS strategy. TB patients require testing methods with higher sensitivity, higher specificity, and lower costs to reduce their need for visits to health-care facilities, along with more instructions to improve their adherence to treatment, leading to lower re-treatment rates.

Acknowledgments: The authors thank the participants who responded to our questionnaires and the TB doctors who participated in the questionnaire survey. We also express our deep gratitude to the staffs of

the Bao'an Chronic Disease Prevention and Cure Hospital and the Center for Tuberculosis Control of Guangdong Province.

References

1. Global tuberculosis report 2018. World Health Organization: Geneva, Switzerland, 2018. Licence: CC BY-NC-SA 3.0 IGO.

2. China Tuberculosis Control Collaboration. The effect of tuberculosis control in China [J]. Lancet 2004, 364, 417–422.

3. Yu, X.; Song, H.F.; Feng, Y.J.; Ye, Z.J. Significance of Gene Xpert-MTB/RIF assay in diagnosis of tuberculosis. J. Practical Med. 2019, 35, 985–988.

4. Liu, Y.; Xu, C.H.; Wang, Z.Y.; et al. [A cross-sectional study on economic burden of pulmonary tuberculosis cases from designated tuberculosis hospital]. Zhonghua Liu Xing Bing Xue Za Zhi. 2019, 40, 559–564 [article in Chinese].

5. Chen, S.; Zhang, H.; Pan, Y.; et. al. Are free anti-tuberculosis drugs enough? An empirical study from three cities in China [J]. Infectious Diseases of Poverty. 2015, 4, 47.

6. Stracker, N.; Hanrahan, C.; Mmolawa, L.; et al. Risk factors for catastrophic costs associated with tuberculosis in rural South Africa. Int. J. Tuberc. Lung Dis. 2019, 23, 756–763.

7. Li, Y.; Ehiri, J.; Tang, S.; et al. Factors associated with patient, and diagnostic delays in Chinese TB patients: a systematic review and meta-analysis. BMC Med. 2013, 11, 156.

8. Qiu, S.; Pan, H.; Zhang, S.; et al. Is tuberculosis treatment really free in China? A study comparing two areas with different management models. PLoS One. 2015, 10, e0126770.

9. Lönnroth, K.; Raviglione, M. The WHO's new End TB Strategy in the post-2015 era of the Sustainable Development Goals. Trans. R. Soc. Trop. Med. Hyg. 2016, 110, 148–150.

10. Guan, H.Y.; Yang, Y.Z.; Tan, W.G.; et al. Effect evaluation of Shenzhen tuberculosis control and prevention program implementation from 2001 to 2010 [J]. Chin. J. Anti-tuberculosis. 2013, 9, 729–737.

11. Liu, J.H.; Zhang, L.; Lu, T.W.; et al. TB control strategy of the preliminary analysis in Bao'an District [J]. Prog. Mod. Biomed. 2010, 21, 4116–4117.

12. de Cuevas, R.M.; Lawson, L.; Al-Sonboli, N.; et al. Patients direct costs to undergo TB diagnosis. Infect. Dis Poverty. 2016, 5, 24.

13. P. Vatcheva, K. and M. Lee, Multicollinearity in Regression Analyses Conducted in Epidemiologic Studies. Epidemiology: Open Access, 2016. 06(02)

14. Feng, C., et al., Log-transformation and its implications for data analysis. Shanghai Arch Psychiatry, 2014. 26(2): p. 105-9.

15. ZHANG Juanjuan, HU Fangxiang, WANG Yunxia, et al. Epidemiological characteristics of tuberculosis in Bao'an District, Shenzhen, 2008-2015. China Tropical Medicine，February 2018，Vol.18，No.2:p128-130

16. Ukwaja, K.N.; Modebe, O.; Igwenyi, C.; et al. The economic burden of tuberculosis care for patients and households in Africa: a systematic review [J]. Int. J. Tuberc. Lung Dis. 2012, 16, 733–739.

17. Suen, S.C.; Bendavid, E.; Goldhaber-Fiebert, J.D. Cost-effectiveness of improvements in diagnosis and treatment accessibility for tuberculosis control in India. Int. J. Tuberc. Lung Dis. 2015, 19, 1115–1124.

18. Pan, H.Q.; Bele, S.; Feng, Y.; et al. Analysis of the economic burden of diagnosis and treatment of tuberculosis patients in rural China [J]. Int. J. Tuberc. Lung Dis. 2013, 17, 1575–1580.

19. Gospodarevskaya, E.; Tulloch, O.; Bunga, C.; et al. Patient costs during tuberculosis treatment in Bangladesh and Tanzania: the potential of shorter regimens [J]. Int. J. Tuberc. Lung Dis. 2014, 18, 810–817.

20. Hu, D.; Long, Q.; Chen, J.; Wang, X.; Huang, F.; Ji, J.S. Factors influencing hospitalization rates and inpatient cost of patients with tuberculosis in Jiangsu Province, China: an uncontrolled before and after study. Int. J. Environ. Res. Public Health. 2019, 16.

21 Onazi, O.; Gidado, M.; Onazi, M.; et al. Estimating the cost of TB and its social impact on TB patients and their households. Public Health Action. 2015, 5, 127–131.

22. Mauch, V.; Bonsu, F.; Gyapong, M.; et al. Free tuberculosis diagnosis and treatment are not enough: patient cost evidence from three continents [J]. Int. J. Tuberc. Lung Dis. 2013, 17, 381–387.

23. Tanvejsilp, P.; Loeb, M.; Dushoff, J.; Xie, F. Healthcare resource uses and out-of-pocket expenses associated with pulmonary TB treatment in Thailand. Pharmacoecon. Open. 2018, 2, 297–308.

24. Pedrazzoli, D.; Siroka, A.; Boccia, D.; et al. How affordable is TB care? Findings from a nationwide TB patient cost survey in Ghana. Trop. Med. Int. Health. 2018, 23, 870–878.

25. Asres, A.; Jerene, D.; Deressa, W. Pre- and post-diagnosis costs of tuberculosis to patients on Directly Observed Treatment Short course in districts of southwestern Ethiopia: a longitudinal study. J. Health Popul. Nutr. 2018, 37, 15.

26. Tang, S.; Wang, L.; Wang, H.; Chin, D.P. Access to and affordability of healthcare for TB patients in China: issues and challenges. Infect. Dis. Poverty. 2016, 5, 10.

27. Jia, X.; Chen, J.; Zhang, S.; Dai, B.; Long, Q.; Tang, S. Implementing a "free" tuberculosis (TB) care policy under the integrated model in Jiangsu, China: practices and costs in the real world. Infect. Dis. Poverty. 2016, 5, 2.

28. Fuady, A.; Houweling, T.A.J.; Mansyur, M.; Burhan, E.; Richardus, J.H. Effect of financial support on reducing the incidence of catastrophic costs among tuberculosis-affected households in Indonesia: eight simulated scenarios. Infect. Dis. Poverty. 2019, 8, 10.

29. Mullerpattan, J.B.; Udwadia, Z.Z.; Banka, R.A.; Ganatra, S.R.; Udwadia, Z.F. Catastrophic costs of treating drug resistant TB patients in a tertiary care hospital in India. Indian J. Tuberc. 2019, 66, 87–91.

30. Tanimura, T.; Jaramillo, E.; Weil, D.; Raviglione, M.; Lönnroth, K. Financial burden for tuberculosis patients in low- and middle-income countries: a systematic review. Eur. Respir. J. 2014, 43, 1763–1775.

31. Gebreegziabher, S.B.; Bjune, G.A.; Yimer, S.A. Patients' and health system's delays in the diagnosis and treatment of new pulmonary tuberculosis patients in West Gojjam Zone, Northwest Ethiopia: a cross-sectional study. BMC Infect. Dis. 2016, 16, 673.

32. Li, Y.; Ehiri, J.; Tang, S.; et al. Factors associated with patient, and diagnostic delays in Chinese TB patients: a systematic review and meta-analysis [J]. BMC Med. 2013, 11, 156.

33. Bogale, S.; Diro, E.; Shiferaw, A.M.; Yenit, M.K. Factors associated with the length of delay with tuberculosis diagnosis and treatment among adult tuberculosis patients attending at public health facilities in Gondar town, Northwest, Ethiopia. BMC Infect. Dis. 2017, 17, 145.

34. Osei, E.; Akweongo, P.; Binka, F. Factors associated with DELAY in diagnosis among tuberculosis patients in Hohoe Municipality, Ghana. BMC Public Health. 2015, 15, 721.

35. Pichenda, K.; Nakamura, K.; Morita, A.; et al. Non-hospital DOT and early diagnosis of tuberculosis reduce costs while achieving treatment success [J]. Int. J. Tuberc. Lung Dis. 2012, 16, 828–834.

36. Duan, W.; Zhang, W.; Wu, C.; et al. Extent and determinants of catastrophic health expenditure for tuberculosis care in Chongqing municipality, China: a cross-sectional study. BMJ Open. 2019, 9, e026638.

37. Jakubowiak, W.; Bogorodskaya, E.; Borisov, S.; et al. Treatment interruptions and duration associated with default among new patients with tuberculosis in six regions of Russia [J]. Int. J. Infect. Dis. 2009, 13, 362–368.

38. Mekonnen, H.S.; Azagew, A.W. Non-adherence to anti-tuberculosis treatment, reasons and associated factors among TB patients attending at Gondar town health centers, Northwest Ethiopia. BMC Res. Notes. 2018, 11, 691.

39. de Faria Gomes, N.M.; da Mota Bastos, M.C.; Marins, R.M.; et al. Differences between risk factors associated with tuberculosis treatment abandonment and mortality. Pulm. Med. 2015, 2015, 546106. doi:10.1155/2015/546106.

40. Wanyonyi, A.W.; Wanjala, P.M.; Githuku, J.; Oyugi, E.; Kutima, H. Factors associated with interruption of tuberculosis treatment among patients in Nandi County, Kenya 2015. Pan Afr. Med. J. 2017, 28(Suppl 1), 11.

41. Long, Q.; Smith, H.; Zhang, T.; et al. Patient medical costs for tuberculosis treatment and impact on adherence in China: a systematic review [J]. BMC Public Health. 2011, 11, 393.

42. Gorityala, S.B.; Mateti, U.V.; Konuru, V.; Martha, S. Assessment of treatment interruption among pulmonary tuberculosis patients: a cross-sectional study. J. Pharm. Bioallied Sci. 2015, 7, 226–229.

43. Ukwaja, K.N.; Alobu, I.; Abimbola, S.; et al. Household catastrophic payments for tuberculosis care in Nigeria: incidence, determinants, and policy implications for universal health coverage [J]. Infect. Dis. Poverty. 2013, 2, 21.

44. Zhou, C.; Long, Q.; Chen, J.; et al. Factors that determine catastrophic expenditure for tuberculosis care: a patient survey in China. Infect. Dis. Poverty. 2016, 5, 6.

45. Bao, H.; Liu, K.; Wu, Z.; et al. Tuberculosis among full-time teachers in Southeast China, 2005-2016. Int. J. Environ. Res. Public Health. 2018, 15, 2024. Published 2018 Sep 17. doi:10.3390/ijerph15092024.

46. Zhu, L.; Yang, Y.Z.; Guan, H.Y.; et al. Trends in drug-resistant tuberculosis after the implementation of the DOTS strategy in Shenzhen, China, 2000-2013. Int. J. Tuberc. Lung Dis. 2017, 21, 759–765. doi:10.5588/ijtld.16.0759.

Data file

Click here to access/download
Supporting Information
data.xls

Analysis of the Economic Burden of Diagnosis and Treatment on Patients with non-drug resistance Tuberculosis (TB) in the TB Control Demonstration Area of China

Yixiang Huang[1,#], Jianying Huang[2,#], Xiaoting Su[1], Liang Chen[3], Jianwei Guo[1], Weiqing Chen[4,*] and Lingling Zhang[5]

[1] Department of Health Policy and Management, School of Public Health, Sun Yat-sen University, Guangzhou, 510080, China
[2] Guangdong Women and Children Hospital, Guangzhou, 511442, China
[3] Centre for Tuberculosis Control of Guangdong Province, Guangzhou, 510630, China
[4] Department of Medical Statistics and Epidemiology, School of Public Health, Sun Yat-sen University, Guangzhou, 510080, China
[5] Department of Nursing, College of Nursing and Health Sciences, University of Massachusetts, Boston 02125-3393, USA
*Correspondence: chenwq @mail.sysu.edu.cn
# Yixiang Huang and Jianying Huang contributed equally to this work.

Abstract: Background: Illness-related costs ~~expenditures~~ experienced by patients produce a severe economic impact on households, especially those of poor families. Few studies have investigated the full costs, including direct and opportunity costs, at the patient and household levels in the south-east of China. Methods: A case follow-up study was conducted in the Bao'an district~~,~~ of Shenzhen City, China. Eligible new and re-treated individuals with pulmonary TB ~~in the Bao'an district~~ during ~~1~~ January 1st 2013 to 30th June 2013~~the study period~~ were enrolled. Medical and non-medical costs as well as income loss were calculated in pre-diagnosis and post-diagnosis periods, respectively. Factors ~~affecting~~ associated pre-diagnosis ~~high~~ costs and post-diagnosis costs were explored respectively with a linear regression model~~multiple regression model~~. Results: Of the total 514 TB patients enrolled, most were from the migrant population, and 65% were males (336), with a mean age of 32.25 (±10.11) years. The median pre-diagnosis direct costs, direct post-diagnosis costs, and post-diagnosis medical and non-medical costs were 79USD, 748USD, 542USD, and 150USD (6.2897CNY = 1USD, 2013) per patient, respectively. The median total cost of TB treatment per patient was 1218USD, corresponding to 26% of patients' annual income pre-illness. ~~Factors~~ Variables associated with ~~out-of-pocket~~ pre-diagnosis costs included number of times visiting health facilities, whether in hospital due to TB , education, History of TB. Variables associated with post-diagnosis costs ~~was~~were household registration, sputum smear status, ~~eported~~reported-household economic burden, ~~martials~~marital status~~history of TB, and the degree of symptoms at diagnosis.~~ Conclusions: Although a free TB control policy is in force, patients with TB are still facing a heavy economic burden. More available interventions to reduce the financial burden on tuberculosis patients are urgently needed.

Keywords: Tuberculosis; cost of illness; economic burden; China

---

1. Introduction

Tuberculosis (TB) is one of the top 10 causes of death worldwide as well as being the leading cause from a single infectious agent (more than HIV/AIDS). According to 2018 global estimates, there were almost 10 million new cases of TB and 1.3 million TB deaths in 2017 [1]. The high prevalence of TB is always a critical public health problem in China, accounting for the second highest number of cases in the world except in India, with 778,000 identified cases of TB in 2017 [1]. Since 1990, the WHO-recommended directly observed treatment + short course chemotherapy (DOTS) strategy to control this epidemic has been implemented by the Chinese government under the National Tuberculosis Control Program [2], whose guidelines include free anti-TB drugs for 6 months to new patients with TB and for 8 months to re-treatment patients in addition to multi-drug resistant/ extensively-drug resistant (MDR/EXDR) patients. Tests include one free X-ray test for individuals suspected of having TB at their first visit, one free sputum smear test for those whose X-ray tests are abnormal~~,~~ and free sputum smear tests for patients undergoing free anti-TB treatment. With the development of molecular biology, a new testing method called GeneXpert/RIF (abbreviated as Xpert) is recommended. With a shorter time of testing, higher sensitivity, and higher specificity, Xpert has been rapidly adopted for clinical diagnoses in China [3].

However, many investigators have pointed out that both the TB patients and their households bear a heavy economic burden. Even the free DOTS control strategy is far from sufficient [4, 5]. Tuberculosis is a well-known poverty-related disease, an association that has been well-established at both patient and household levels, usually related to the disadvantages of socio-economic status. In particular, among those who come from rural and

poor districts, the scarcity of health resources and their poor economic condition place them in a more serious situation [6]. The financial aspect is a major factor contributing to health-care-seeking behavior among Chinese patients. Delayed and repeated visits to clinics before diagnosis, the over-prescribing of drugs, and prolonged treatment are common [7]. Patients must bear ~~heavy~~ too much ~~out-of-pocket expenditures~~economic burden, such as, for example, medical and transportation costs, and income loss, precluding them from further adhering to treatment and resulting in treatment failure [5].

There have been many studies on the cost-effectiveness of different TB control strategies, mainly investigating the costs to the health system in the Eastern, Middle, and Western Regions of China. These studies focused mainly on the effectiveness of medical insurance for coping with the costs of illness as well as patient compliance with drug regimens [5, 8]. However, far fewer studies have measured the costs to patients and households or the full costs, including direct and opportunity costs, from the onset of symptoms to the end of treatment in the south-east of China. To achieve the health-related Sustainable Development Goal (SDG) proposed by the United Nations and the goal of ending the TB epidemic by 2035 proposed by WHO, whose last target reads “No TB patients and their families face Catastrophic costs (>20% of annual household income)” [9], all TB patients should receive accessible and affordable standard medical treatment. Therefore, the present study was performed to evaluate the out-of-pocket direct costs as well as productivity lost related to illness. Potential factors associated with these costs were also explored. We aimed to establish the evidence base for interventions that can contribute to TB-related cost reduction, increase patient adherence to treatment, and treatment success rates. There is a need to document the full economic burden of TB care borne by patients and their households.

2. Materials and Methods

2.1. Study Setting

Shenzhen City is one of the most prosperous regions in China, with a Gross Domestic Product (GDP) of nearly ~~225~~ 269 million CNY in ~~2017~~2019. Its estimated population is over ~~12~~ 13 million, with the migrant population constituting 65.3% while this rate is 82.4% in Bao’an district of the total regional population according to the Shenzhen statistical yearbook 2019. ~~(http://tjj.sz.gov.cn/)~~. The mean household income in the region was 6,656.79 USD of 2012 according to the Statistical yearbook 2013. The rapid economic growth in the city, leading to higher income levels, attracts numerous young migrant workers, mostly from remote poor areas exposed to substandard conditions such as lower societal status, poor standard of living, limited health knowledge, and less accessibility to health services, thus creating favorable conditions for infection with Mycobacterium tuberculosis and progression to disease. Such conditions delay individuals from seeking health care, resulting in advanced disease and continued transmission of TB in the community. Although analysis of recent data has shown a decreasing incidence of TB in the Shenzhen region, its prevalence in the Bao'an district, where a heavy concentration of migrant workers contributes to a high TB case load, is still high.

The Bao'an district implemented the 10-year World Bank TB project in 1992, brought the migrant population into the National TB control program (NTP), and adapted the DOTS strategies. After this ended in 2002, the Shenzhen TB control project in Guangdong province was launched. Then, as a representative of Guangdong province, the Bao'an district was selected as one of the seven counties to implement the fifth round of the globally funded TB control project. Up to 2010, the Bao'an district was selected by the China Ministry of Health as the site of a demonstration area for a tuberculosis control model. ~~Since then, the average diagnosis and cure rates have greatly improved.~~ The ~~hosptials~~hospitals were the main palce finding tuberculosis case and the Center for Prevention and Cure of chronic diseases (CPC) were the local designated TB facilities in the national TB program~~me~~ networks, providing treatment and managing the tuberculosis cases. The healthcare providers in general hospitals were obligate to refer the

suspected cases to CPC and the basic information of suspected cases were sent to CPC. If the cases did not consult~~ed~~ to CPC in time, the workers of CPC would contact the suspected case. Actively cooperating with medical institutions and TB prevention and control institutions, the discovery rate and report quality was increasing by years. In 2010, the prevalence rate of smear positive of native population was 19.44 per 100 000 population, and that of the migrant population was 59.96 per 100 000 population and the cure rates for both native and migrant patients were 94.79% and 82.91%, respectively [10]. The main health-care sources in China are district public health facilities organized into three tiers of referral: primary (Center for Prevention and Cure of chronic diseases), secondary (sub-district prevention and care dispensaries), and tertiary (Community health-care stations) [11]. The DOTS program means directly observed treatment +short course chemotherapy for non-inpatient tuberculosis patients, and is available only in public health facilities, where patients have free access to diagnostic and treatment services. The Center for Prevention and Cure of chronic diseases (CPC) is the institution authorized to provide TB diagnosis, treatment, and monitoring. The CPC provides patients with ~~laboratory services like sputum smear testsand~~ a~~n~~ radiologic imaging studies (X-ray) while diagnos~~is~~ed, and sputum smear tests during treatment,~~after diagnosis, as well as regular re-examinations~~ and anti-TB drugs for 6-8 months for free. Patients must regularly return to CPC every month after starting to take anti-TB drug until the treatment is over. Generally, the number of times of subsequent consultations are 5 and 7 to newly-patents and re-treated patients respectively. The costs of ervery month including auxiliary examinations and subsidiary drugs like hepatinica, gastric medicine, and immunopotentiator etc are not free.~~Other costs, however, including auxiliary examinations and subsidiary drugs~~ like ~~hepatinica, gastric medicine, and immunopotentiator etc,, are not free.~~ The sub-district prevention and care dispensaries and Community health-care stations coordinating with the CPC are obligated to perform routine monitoring, referral, and supervision. Adherence is directly monitored by the health workers of Community health-care stations for daily drug intake, adverse reactions, and other activities of admitted patients. If patients fail to take a specific drug for 2 days, the workers will contact them by telephone to ensure that the patients complete their treatment.

2.2. Study Design and population

A case follow-up study was conducted in the Bao'an district with an ongoing NTP-DOTS project. Newly diagnosed and re-treated TB patients aged 15-59 years old, who registered in the CPC during~~from~~ 1 January 2013 to June 2013, were included and were followed up until the completion of a 6- to 8-month treatment course. Those with severe co-morbidities ~~complications~~ such as AIDS, diabetes, and lung cancer and those who refused to accept investigation were excluded. The patients aged above 59 were excluded considering the elderly may have more ~~comorditites~~commodities and those with MDR-TB were excluded too.Transferred patients were also excluded from the study, since information on previous ~~out-of-pocket expenditures~~costs was not available. ~~Consecutive consenting eligible patients were interviewed until the required sample size was reached.~~ The operational definition of various costs and indicators were summarized in table 1.

2.3. Data Collection

All eligible cases were subjected to five (new patients) or seven (re-treatment patients) interviews at the Department of Tuberculosis Prevention and Control in Bao'an CPC. A structured questionnaire was used, covering general demographic and socio-economic characteristics, disease history, care-seeking process, treatment behaviors, and ~~out-of-pocket expenditures~~costs ~~both~~ of pre- and post-diagnosis. Self-reported economic status information was collected based on different localities with various levels of development. The first interview was at the first month of intensive phase of treatment, when patients' retrospective

care-seeking history and related expenditures, including pre-diagnosis, starting from the time point when the patients experienced the first symptoms, and the intensive phase related costs, were collected. The other four or six interviews occurred monthly when patients came for regular re-examinations. The patients' TB care experiences and transportation fee, supplementary food cost, and foregone income of patients and companions ~~expenditures after the intensive phase~~ were collected in follow-ups.

A graduate student with two locally recruited research assistants, health workers who had participated in a standardized training session, conducted those interviews. A logic check of all data collected was undertaken to determine if there were any contradictions or missing information. Those ~~contradicions~~contradictions or missing information ~~questions~~ would be asked of patients again in a subsequent interview. We also evaluated the quality of collected data by verifying the routine health information system.

2.4. Measurements and Definitions

We ~~analysis~~nanlyzed the economic burden from tuberculosis patients' perspective. The primary outcome variables were the mean (median) expense incurred during illness.~~.~~ ~~The primary outcome variables were the mean (median) number of patients and household costs incurred.~~ Information on costs was ascertained for different periods, summarized as pre- and post-diagnosis.

Direct costs included all ~~out-of-pocket expenditures~~costs of patients and companions attributable to their illness [12], which consisted of the medical costs related to TB diagnosis and treatment (clinical and hospitalization expenses), and non-medical costs incurred by patients or ~~households~~companions, such as transportation to health facilities and supplementary food. Opportunity costs referred to the income lost by patients and their ~~households~~ companions associated with time lost ~~from~~off work. Assuming 30 working days per month, we evaluated the value in terms of money for each day, and opportunity costs was calculated as the value per day multiplied by the length of time ~~patients were out~~off ~~of~~ work due to illness. The income lost before diagnosis was calculated based on each patient's monthly wage prior to the onset of TB. The lost income after diagnosis was calculated based on the monthly wage if patients were still on working during illness.~~To quantify income lost~~ ~~after~~After diagnosis, patients were asked about the actual ~~reduction of income~~ lost income that they and their companions had experienced due to absence from their usual income-generating activities at every interview. These monthly income reductions were then summed. The income lost by companions pre-diagnosis was not included in the analysis, since only a few such losses occurred. The time spent on daily drug intake was also not calculated, because most patients took drugs on their way to work or at the community health-care stations close to their homes.

The economic burden was then analyzed as a proportion of patients' total annual income. The free services provided during diagnosis and treatments were not included in the medical costs. Information about costs after diagnosis was extracted from the information system of the study facility. Information on costs covered by insurance could not be obtained, since the insurance had not been settled at the end of the interview.

Table 1. Operational definition of study participants and TB treatment cost in the study of Bao'an district, Shenzhen City, China, 2013

Terminology	Definition
Study participants	All newly diagnosed and re-treated TB patients accepted treatment in CPC during 1 January 2013 to June 2013
Study participants ineligible	Patients with severe co-morbidities such as AIDS, diabetes and lung cancer with multidrug-resistant TB, aged>59 were excluded.
Pre-diagnosis costs	Costs between symptom onset and diagnosed as TB
Post-diagnosis costs	Costs from treatment initiation up to treatment completion

Direct medical costs	Expenses of medical examinations and medicines linked to TB diagnosis and treatment
Direct non-medical costs	Costs for transport, food expenditures ,nutrition supplements due to TB
Direct cost	Direct medical costs + direct non-medical costs
Opportunity costs	Patients and companions' lost income due to TB-related time off work during the TP episode. The pre-diagnosis opportunity cost was estimated using the time off work multiplied by the reported individual income prior to the onset of TB
Total costs	Direct costs + opportunity costs
Sputum smear test	At the time of diagnosing conducted one sputum smear test, the outcome included negative and positive.
Household economic burden	Self-reported economic burden due to TB treatment
The degree of symptoms at diagnosis	The patients self-reported with different symptoms at diagnosis were divided into four categories. 1. None: no symptoms; 2.mild: with symptoms included cough, expectoration and dyspnea, night sweat, and debilitation; 3. Moderate: symptoms with fever and chest distress; 4. severe: symptoms like hemoptysis.
Interruption of treatment	Treatment interruption is defined as any interruption of treatment for at least one day but for < 8 consecutive weeks

2.5. Data Analysis

The data were double-entered with EpiData 3.1 software (EpiData Association, Odense, Denmark) for each patient, and cost summaries and analyses were performed by SPSS 20.0 software. Continuous variables like direct costs, opportunity costs were summarized as means (± standard deviation [SD]) or medians (IQR), while categorical variables were summarized as proportions. Group comparisons were made with the *rank correlation*~~t-test~~ for ~~means~~median, the Mann-Whitney test or Kruskal-Wallis test for medians in two or multiple groups~~.~~ ~~, and 2 tests for proportions.~~if their distribution were non-normal.

We also did a confounder adjusted analysis for the association between pre-diagnosis costs or post-diagnosis costs and predictor variables respectively using linear regression. The costs were log transformed as they were not normally distributed. To explore more variables as predictor variables, the possible variables were all considered in the linear regression model irrespective the value in univariate group comparisons. A forward stepwise approach was used to find the appropriate model. Association was summarized using Beta coefficient (95% CI), *P*-value less than 0.05 were significant. Considering the multicollinearity in predictor variables, we calculated variance inflation factor (VIF) at the same time [13], If VIF was less than 10, then the predictor variables were included in the linear model.

Considering in applying and interpreting the results of hypothesis testing in a log-transformed data on actual data (non-log transformed) [14]. Hence, we also conducted a confounder-adjusted association between costs and predictor variables using generalized linear model (logistic regression, the costs were divided as binomial variables based on median value. To explore more variables as predictor variables, the possible variables were all considered in the linear regression model irrespective the value in univariate group comparisons. Association was summarized using exp(β) coefficient (95% CI), P-value less than 0.05 were significant.

~~Since the costs did not follow a normal distribution, we converted them to a logarithm, and then used a multiple regression analysis to determine factors associated with the economic burden due to TB illness. First, bivariate analyses were done with the Chi-square test or Mann-Whitney analysis to examine the effect of each predictor variable on economic burden. Then, the multivariable model was constructed, including all variables with bivariate *p* < 0.1. A forward stepwise approach was used to find the appropriate model. *P*-values less than 0.05 were significant. All of this was performed with SPSS 20.0 software.~~

2.6. Ethics Approval

This study was approved by the research and ethics committee of the School of Public Health, Sun Yat-Sen University, China. Also, permission was obtained from the study site. A signed informed consent was obtained from all patients before being interviewed.

3. Results

3.1. Socio-demographic Characteristics and Care-seeking Behaviors

The present study surveyed 533 eligible pulmonary tuberculosis patients who with no co-morbidity, 19 of whom were lost to follow-up, one of whom died from another disease, and 18 of whom moved to other provinces. The rate of loss to follow-up was nearly 4%. Among the 514 patients receiving anti-TB treatment, 30 were re-treatment patients, and 95% were members of the migrant population from all over the country. The average age was 32.25 ± 10.11 years, 65% (336/514) of the patients were male, and 35% were female. The mean monthly income of patients before illness was 448USD, ~~A~~about 71.4% of patients had monthly household income between 317.98 to 635.96 USD.~~and the range of annual household income was between 795 and 15,899USD. About 71.4% of patients had monthly household income between 2000 to 4000.~~

One hundred seventy-six patients were identified when they accepted health check-ups required by their work units, and were then ~~immediately~~ transferred to the CPC~~,~~. They were diagnosed at CPC immediately without ~~consulating~~consulting to other facilities, so their pre-diagnosis costs were not calculated. ~~so no transportation fee or time loss occurred before diagnosis.~~ Three hundred thirty-eight patients were registered as a result of clinical consultation. Among the 338 patients, the median delay time of visiting doctors, defined as the period between onset of symptoms and first contact with a health-care provider, was 11 days (IQR 3-30), for reasons such as having no time, being unaware of the presence of tuberculosis, and lacking money. The median number of consultations prior to diagnosis ~~-~~was 3 (1, 20). The number of first visit in community healthcare center and general hospital was 71(21%) and 136(40.2) respectively. Only 48~~14%~~ (48~~14~~%)~~-of~~ patients initially visited a public facility, while the others reported that they first consulted either a drug shop or private ~~hospital~~clinic or did nothing. Then median delay time of diagnosis was about 7 (0,200) days.

~~Variable~~	~~Number (%)~~
~~Age (years)~~	
~~< 20~~	~~20 (3.89%)~~
~~20–30~~	~~238 (46.3%)~~
~~30–40~~	~~143 (27.82%)~~
~~40–50~~	~~70 (13.62%)~~
~~>50~~	~~43 (8.37%)~~
~~Gender~~	
~~Male~~	~~336 (65.40%)~~

Female	178 (34.60%)
**Type of TB**	
Newly diagnosed patients	484 (94.16%)
Re-treatment patients	30 (5.84%)
**Marital status**	
Single	180 (35.02%)
Married	308 (59.92%)
Other (divorced, cohabiting)	26 (5.06%)
**Education**	
Illiterate or primary school	63 (12.26%)
Junior high school	220 (42.80%)
Senior high school	169 (32.88%)
Junior college and above	62 (12.06%)
**Occupation**	
Factory worker	316 (61.48%)
Individual business	46 (8.95%)
Other (include company stuff/service personnel)	99 (19.26%)
Unemployed	53 (10.31%)
**Residence**	
Native	26 (5.06%)
Migrant	488 (94.94%)
**Self-reported economic status***	
High	30 (5.84%)
Medium	323 (62.84%)
Low	161 (31.32%)
**First visited a public facility**	
Yes	48 (9.34%)
No	466 (90.66%)
**Annual household income** (USD)	
<795	13 (2.53)
795-2385	44 (8.56)
2385-4770	114 (22.18)
4770-9539	133 (25.88)
9539-15,899	113 (21.98)
>15,899	97 (18.87)
Insurance	
Labor insurance	186 (36.19)
Self-paying	305 (59.34)
Unclear	23 (4.47)

* Self-reported economic status was based on the local condition of patients' domiciles.

3.2. Pre-diagnosis Direct Costs

Among the 338 patients who incurred costs due to ~~consulation~~consultation~~expenditures~~, the median outpatient medical cost incurred pre-diagnosis was 64USD (IQR 24-159) per patient. Twenty-two patients were admitted to the hospital before TB was diagnosed. Hospital patients incurred a substantial higher out-of-pocket expenditure. The median hospital expenditure was 477USD (IQR 318-823). The non-medical costs refer to transportation and accommodation fees. This study surveyed only the transportation fees for patients before diagnosis. The median transportation fee was 2USD (IQR 0-8). Costs due to the prescribing of non-TB drugs and diagnosis costs accounted for a high proportion of direct expenditures in the pre-diagnosis period (97.0%).

3.3. Post-diagnosis Direct Costs

A patient's average number of health-care visits after TB was diagnosed was 9.7, with a maximum of 15 visits. The direct costs after TB was diagnosed were 748USD (IQR 567-987); the average cost for one visit was 77USD. Median medical cost was 542USD (IQR 465-618), then we further analyzed what constituted medical costs. Median costs for examinations and non-TB drugs were 307USD (IQR 273-341) and 211USD (IQR 166-265), respectively, which were clearly the most robust components, accounting for 55% and 40% of medical costs, respectively. Meanwhile, the auxiliary drugs, such as liver/kidney protection drugs and immunopotentiators, constituted the main part of non-TB drugs, with a median cost of 174USD (IQR 135-219), accounting for 83% of the non-TB drug costs.

The non-medical costs in the post-diagnosis period included transportation generated by patients and companions and supplementary food for patients. The median transportation cost was 19USD (IQR 10-31); the average cost for a single event was 3USD. Among the surveyed patients, 196 were escorted during treatment by their family or friends, and the median transportation cost was 5USD (IQR 2-13). About 340 patients improved nutrition with supplementary food, and the median cost was 302USD (IQR 127-489). The non- medical costs totaled 150USD (IQR 29-410). Clearly, the non-medical costs were relatively low (Table 2).The direct costs of intensive phase (the first two month) and consolidation phase(the next 4 or 6 month) ~~was 1324.5(1037,1759) and 3248.5(2465,4424.1)~~ 210.58(164.87,279.66) USD and 516.48(391.91,703.39) USD respectively. The direct costs of consolidation phase accounted for 70.7% of total pre-~~diganosis~~diagnosis direct costs.

3.4. Opportunity Costs

Only 49 patients reported income lost while away from work due to seeking health care before diagnosis. Income lost by those patients, incurred during the pre-diagnosis period up to standard anti-TB treatment, was 32USD (IQR 16-83). Most of the opportunity costs were from lost income after TB was diagnosed (Table 2), because most patients were fired or could not physically continue working. For the pre-diagnosis period, the income loss was calculated based on each patient's daily income before illness (median, 32USD), multiplied by the total time the patient was out of work due to illness (median, 2 days, $n = 49$), mainly due to travel and consultation time at different health-care facilities. For the post-diagnosis period, the monthly income reduction for a complete treatment cycle was summed. The income lost at two stage of treatment was 31.80(0, 63.60) USD and 127.19(38.16, 683.66) USD~~200(0,400) and 800(240,4300)~~. Most income lost ~~comed form~~came from the consolation phase. The opportunity cost du~~i~~ring consolidation period accounted for 79% of total post-diagnosis ~~oportunity~~opportunity cost. The time lost due to daily drug intake was not calculated in the current study.

3.5. Total Patient and Household Costs

The sum of median direct and opportunity patient and household costs was 1218USD (IQR 826-1963), which corresponds to 26% of annual individual incomes before TB illness (4770USD). The direct costs accounted for 61% of the total costs, and the ratio of direct to opportunity costs was 1.58. About 88.2% (1074/1218) of costs (Fig 1) were encountered after the patients were diagnosed and accepting free DOTS treatment.

Figure 1. All patients' average cost during different episodes of tuberculosis treatment. *The median of opportunity costs before diagnosis was zero.

Figure 1 legends: direct cost opportunity cost

Table 2. Mean MeidanMedian direct and opportunity costs of TB patients during different episodes of ttreatment in the study of Bao'an district, Shenzhen City, China, 2013.

Timing and Types of Costs	Patient-reported costs during this Period			All Patients***
	Number*	%	Median** (IQR range)	(n = 514)
**Pre-diagnosis**				
**Direct costs**				
Outpatient medical costs	338	66	64 (24-159)	19 (0-79)
Hospital costs	22	4	477 (318-823)	0 (0-0)
Transportation costs	338	66	2 (0-8)	0 (0-3)
**Pre-diagnosis direct costs[d]costs^**				**79 (24-191)**
**Opportunity costs**				
Foregone income before diagnosis	49	10	32 (16-83)	0 (0-0)
**Median cost of pre-diagnosis[d]**				**79 (25-204)**
**Post-diagnosis**				
**Direct costs**				
**Medical costs**	**514**	**100**	**542 (465-618)**	**542 (465-618)**
Examination	514	100	307 (273-341)	307 (273-341)
Non-TB drugs	514	100	211 (166-265)	211 (166-265)
Auxiliary drugs	514	100	174 (135-219)	174 (135-219)
Others	514	100	21 (20-24)	21 (20-24)
**Transportation**				
For patients	514	100	19 (10-31)	19 (10-31)
For companions	196	38	5 (2-13)	0 (0-3)
**Supplementary food**	340	66	302 (127-489)	127 (0-376)
**Post-diagnosis direct costs**				**748 (567-987)**
**Opportunity costs**				
Foregone income of patients	402	78	241 (90-1,181)	114 (24-797)
Foregone income of companions	196	38	39 (16-84)	0 (0-24)
**Median cost of post-diagnosis**				**1074 (745-1820)**
**Total direct cost**				**833 (609-1132)**
**Total opportunity cost**				**157 (57-855)**

**Median cost of all periods**	**1218 (826-1963)**

[a]~~Total~~ *Total number of patients, 514.

[b]~~The~~ **The average cost among patients who generated this kind of ~~expenditure~~costs in row 2.

[c]~~The~~ **The average cost for all patients (514).

[d]~~Pre~~^Pre-diagnosis direct cost = direct costs + transportation costs. Median cost of pre-diagnosis = direct costs + opportunity costs.

3.6. ~~Factors Related to Economic Burden~~The ~~differentials~~association of pre-diagnosis costs among tuberculosis patients

The socio-demographic and care-s~~a~~eeking level characteristics of pre-diagnosis costs were summarized in table ~~2~~3. ~~We explored factors related tocosts and income loss separately. In the single-factor analysis, as shown in Table 3,~~ Those re-treated patients had more costs compared with newly patients (P=0.05) and patients who were in hospital due to tuberculosis generated more costs (P<0.0001). It seemed that the more number of times visiting to health-care facilities (P=0.05), the longer of delay of diagnosis(P<0.0001) and delay of first visiting health-care facilities when symptom onset.

The direction and significance of association between the pre-diagnosis costs and the number of times visiting to health-care facilities, whether in hospitals due to tuberculosis before diagnosis did not changed after a confounder adjusted analysis in linear regression analysis . However~~–~~, the increase in delay of diagnosis was not found to be statistically significant in adjusting analysis (Table ~~5~~4). But we found those who received higher education or occupied in national civil servant/services /retired staff might expense more before diagnosis in the linear regression analysis too. In the corresponding generalized linear regression, number of times visiting health-care facilities was found to be related pre-diagnosis costs statically significant (P<0.0001). (Table ~~6~~5)

3.7 The ~~differentials~~association of post-diagnosis costs among tuberculosis patients

The socio-demographic characteristics of post-diagnosis costs had been depicted in Table 6. Migrant population might have more costs during the tuberculosis treatment compared with native population (p=0.003~~-~~). There were difference of costs after diagnosis as TB among different education level patients (P=0.039). Those who perceived that the TB imposed economic burden to household might have more expenses on the treatment (P=0.021).The status of sputum smear test at the time of diagnosis was positive generate more costs compared to the negative (P<0.0001).

After a confounder adjusted analysis, the direction and significance of association of post-diagnosis costs and the household registration (migrant or native residents), reported economic burden of households, sputum smear status did not changed (Table 7). The divorced or widowed patients had more costs compared to the unmarried in the linear regression analysis (p=0.044). In the corresponding generalized linear regression, only sputum smear status adopted in the model. (P<0.0001). (Table ~~6~~8)

Table 3. Association of pre-diagnosis costs by background characteristics and care-seeking behaviors among tuberculosis patients enrolled in the study of Bao'an district, Shenzhen City, China, 2013

variables	Number	%	median(IQR range) of pre-diagnosis cost	*P*
**Gender**				
Female	116	34.32	68.37(18.28,166.14)	0.121
Male	222	65.68	81.48(31.8,240.39)	
**age**	338	100.00	79.5(25.2,204.43)	0.723
**Household registration**				
Native patients	21	6.21	34.98(23.85,158.99)	0.15
Migrant patients	317	93.79	79.5(25.44,205.63)	
**Marital status**				
unmarried	105	31.07	79.5(23.85,238.49)	0.158
married	217	64.20	79.5(25.44,190.79)	
widowed/divorced	7	2.07	79.5(58.03,203.67)	
others	9	2.66	50.88(19.71,317.98)	
**Education**				
Primary/illiterate	48	14.20	72.5(17.01,168.53)	0.171
Junior high school	139	41.12	66.78(23.85,164.55)	
Senior high school	110	32.54	79.5(31.8,182.84)	
college or above	41	12.13	174.89(47.7,386.88)	
**Occupation**				
Workers	203	60.06	78.7(23.85,222.59)	0.401
Individual business	37	10.95	66.78(23.85,164.55)	
Others (national civil servant/services /retired staff etc.)	64	18.93	123.22(57.63,328.31)	
Unemployed	34	10.06	174.89(47.7,386.88)	
**Number of times visiting health-care facilities**				
<=2	214	63.31	34.18(15.9,95.39)	<0.0001
3~6	101	29.88	158.99(81.08,289.36)	
>=7	23	6.80	724.52(328.79,724.52)	
**health-care facilities for the first time**				
community healthcare center	71	21.01	100.69(37.36,283)	0.05
public facility	48	14.20	95.39(63.6,249.61)	
general hospital	136	40.24	48.02(15.9,174.89)	
private clinics	38	11.24	57.55(23.85,127.19)	

others	45	13.31	87.44(31.8,174.89)	
**delay between symptom onset and first ~~consulation~~consultation**				
<=7	152	44.97	80.29(28.14,271.48)	0.375
7~14	38	11.24	73.93(31.8,174.89)	
14~21	42	12.43	64.39(17.49,119.24)	
>=21	106	31.36	79.5(19.71,174.89)	
delay of diagnosis				
<=7	186	55.03	32.91(15.9,96.67)	<0.0001
7~14	35	10.36	79.5(37.92,167.73)	
>14	117	34.62	166.94(81.08,479.83)	
**Whether in the hospital due to TB**				
yes	22	6.51	1203.28(675.71,1564.46)	<0.0001
no	316	93.49	71.55(23.85,1669.55)	
**The degree of symptoms at diagnosis**				
None	50	14.79	76.63(17.49,280.88)	0.412
Mild	112	33.14	69.96(23.85,172.9)	
Moderate	88	26.04	80.61(31.8,173.94)	
Severe	88	26.04	82.2(31.8,302.08)	
**History of TB**				
New patients	315	93.20	78.7(23.85,190.79)	0.05
Re-treated patients	23	6.80	100.69(48.49,297.98)	

Table 4 Confounder adjusted association between *pre-~~dagnosis~~diagnosis costs* and various predicator variables using linear regression models in the study of Bao'an district, Shenzhen City, China, 2013 (n=338)

Predictors in the model	Beta coefficient	(95% CI)	P value	VIF
**Number of times visiting health-care facilities**	0.12	(0.11,0.14)	< 0.001	1.05
**whether in hospital due to TB (ref: no)**				
yes	-1.02	(-1.2,-0.84)	< 0.001	1.03
**education（ref:Primary/illiterate)**				
college or above	0.26	(0.12,0.39)	< 0.001	1.03
**Occupation(ref:workers)**				

Others (national civil servant/services /retired staff etc.)	0.13	(0.02,0.24)	0.02	1.03
**History of TB (ref: new patients)**				
**Re-treated patients**	0.22	(0.04,0.40)	0.02	1.02

Table 5 Confounder adjusted association between *pre-diagnosis* costs* and various predicator variables using logistic regression models in the study of Bao'an district, Shenzhen City, China, 2013 (n=338)

predictor in the model	B	Exp(B)	(95% CI)	P value
**Number of times visiting health-care facilities**	**0.818**	**2.267**	（1.760,2.919）	**<0.001**
**History of TB (ref: new patients)**				
Re-treated patients	1.001	2.721	（1.037,7.140）	0.042
**Education(ref:Primary/illiterate)**				
Junior high school	-1.760	0.172	（0.062,0.475）	0.001
Senior high school	-1.168	0.311	（0.137,0.705）	0.005
college or above	-.901	0.406	（0.176,0.936）	0.034
**delay of diagnosis**	-.014	0.986	（0.971,1.001）	0.062
**constant**	-1.920	0.147		0.003

*Pre-diagnosis costs in generalised linear model (logistic regression) was categorized based on median value among all participants

Table 6 Association of post-diagnosis costs by background characteristics and care-seeking behaviors among tuberculosis patients enrolled in the study of Bao'an district, Shenzhen City, China, 2013

Variables	Number	%	median(IQR range) of pre-diagnosis cost	p
**Gender**				
Female	178	34.63%	1128.91(764.9,1717.51)	0.309
Male	336	65.37%	1128.91(764.9,1717.52)	

Age	514	100%	1074.38(745.35,1818.69)	0.177
**Household registration**				
Native patients	26	5.06%	760.93(489.53,1277.49)	0.003
Migrant patients	488	94.94%	1096.24(757.65,1852.85)	
**Marital status**				
unmarried	180	35.02%	1103.47(743.68,1864.68)	0.193
married	308	59.92%	1018.08(746.43,1719.76)	
widowed/divorced	10	1.95%	2208.69(844.63,3183.62)	
others	16	3.11%	879.98(691.18,1803.74)	
**Education**				
Primary/illiterate	63	12.26%	859.61(662.77,1398.92)	0.039
Junior high school	220	42.80%	1150.77(827.62,1921.22)	
Senior high school	169	32.88%	1021.42(722.61,1632.99)	
college or above	62	12.06%	1064.25(682.54,1924.26)	
**Occupation**				
Workers	203	60.06%	78.7(23.85,222.59)	0.772
Individual business	37	10.95%	66.78(23.85,164.55)	
Others (national civil servant/services /retired staff etc.)	64	18.93%	123.22(57.63,328.31)	
Unemployed	34	10.06%	174.89(47.7,386.88)	
**Sputum smear status**				
negative	287	55.84%	963.15(699.21,1601.36)	<0.0001
positive	227	44.16%	1222.47(803.39,1982.94)	
**Interruption of treatment**				
Yes	67	13.04%	1084.95(801.68,1412.53)	0.572
No	447	86.96%	1067.95(740,1864.68)	
**Household economic burden**				
Heavy	149	28.99%	1153.54(787.48,1976.25)	0.021
Moderate	236	45.91%	1096.28(740.46,1888.75)	
No burden	129	25.10%	961.76(668.92,1344.58)	
**Adverse drug reaction**				
Yes	268	52.14%	1112.96(743.44,1852.95)	0.447
No	246	47.86%	1009.81(745.35,1792.01)	
**The degree of symptoms at diagnosis**				
None	131	25.49%	1009.75(733.98,1810.5)	0.737

Mild	181	35.21%	1014.74(719.11,1654.26)	
Moderate	103	20.04%	1143.66(770.15,1728.62)	
Severe	99	19.26%	1140.12(757.65,1971.92)	
**History of TB**				
New patients	484	94.16%	1043.25(733.95,1817)	0.064
Re-treated patients	30	5.84%	1293.75(956.37,1911.22)	

Table 7 Confounder adjusted association between *post-diagnosis costs* and various predicator variables using linear regression models in the study of Bao'an district, Shenzhen City, China, 2013 (n=514)

Predictors in the model	Beta coefficient	(95% CI)	P value	VIF
**Sputum smear status（ref：negative）**				
positive	0.08	(0.036,0.128)	< 0.001	1.005
**household registration (ref: Native patients)**				
Heavy	0.15	(0.043-0.254)	0.006	1.032
**reported-household economic burden（ref:no burden)**				
Heavy	-0.06	(-0.109,-0.003	0.040	1.038
**~~martials~~marital status (ref:unmarried)**				
widowed/divorced	0.17	(0.04,0.333)	0.044	1.03

Table 8 Confounder adjusted association between *post-diagnosis* costs* and various predicator variables using logistic regression models in the study of Bao'an district, Shenzhen City, China, 2013 (n=514)

predictor in the model	B	Exp(B)	(95% CI)	P value
**Sputum smear status (ref: negative)**				
positive	0.653	1.921	（1.350,2.735）	<0.001
**constant**	-0.288	0.750		0.016

4. Discussion

This study evaluated the economic burden borne by TB patients in Shenzhen City during their illness. We included 533 eligible patients in the survey, 19 of which were lost to

follow-up. The average age of them was 29.6±10.2years. The male was 14 and female was 5. One patient was with a severe symptoms appeared hemoptysis. The others were with mild or moderate symptoms like cough, chest distress and fever etc. the lost to follow-up was about 4 percent, and the general Socio-demographic and clinical characteristics of those 19 participants lost to follow-up were identical with the 514 patients. So，we considered there no much influence on our conclusion.

As one of the most population of Shenzhen district and owing numbers of factories , the migrant population in Baoan always exceeds the average level of Shenzhen city. The total number of registered active tuberculosis cases were 1317, included 76(5.8%) native case and 1241(94.2%) migrant cases in 2013[15]. In our study, 95 percent were migrant cases, and about 80% of patients were factory workers or service staffs who general were in a poor economic status and poor living environment.~~ As shown above, even in a high-income-level district where the proportion of people with health insurance is relatively higher than in other regions of China, tuberculosis patients do experience high out-of-pocket expenditures. Furthermore,~~Under the DOTS program, the CPC provide tuberculosis patients with a radiologic imaging studies (X-ray) , sputum smear tests and anti-TB drug during treatment for free amounting to 102.07USD.~~ post-diagnosis costs accounted for 88.2% (1074/1218) of total costs.~~ We found that the migrant patients faced much costs during TB ~~treatment.this~~treatment. This may be owing to the different mode of payment and free strategy ~~.~~The current study confirmed that TB patients are from vulnerable groups, and that migrant groups working in factories face worse living conditions and lower average income than the general population.

The median household ~~expenditures~~ costs found in this study (1218USD) were far higher than those in Africa, India, and rural China [16-18]. These differences could partly be explained by the differences in purchasing power among these countries/districts and/or due to the omission of some cost elements in these studies. The more important reason may be the prospective follow-up design of this study, which reduced recall bias. ~~Further, the total costs presented here took into account the fact that patients incurred costs after the end of treatment, required by doctors to consolidate cure for two or three months, depending on the level of patient recovery. Anti-TB drugs and sputum smear tests at this stage were paid for by patients themselves.~~

We found that, one year prior to the study, 78.5% percent of patients had annual household incomes between 795 and 15,899USD, while 13 patients made less than 795USD a year. The direct and opportunity costs corresponded to 26% (1218/4685) of annual personal income. These costs constituted a considerable part of their disposable income after payment of fixed costs such as rent, electricity, and water, along with insurance. Moreover, patients must face these costs for more than 6 months; over a quarter of patients reported that the economic burden of cure was heavy. Twenty-three patients reported incurring debts due to illness. Factory workers and the unemployed had to rely on help from their families. Some authors have concluded that all costs to TB patients account for a higher percentage of their annual income [16, 19-21]. For example, a survey in three countries showed that treatment-related costs were equivalent to approximately one year of individual income [22]. The differences between these studies and ours may be explained by social background, ~~insurance systems,~~ and a higher income level in our study population.

Direct costs are still substantial, with non-TB drugs and auxiliary examinations as the most significant cost items [23-25]. Additional drugs, such as those for protecting the liver/kidney or symptomatic treatment, and examinations must be paid for, and it appears that this led to the high ~~out-of-pocket expenditures~~costs ~~-~~during the treatment period, indicating that there may be over-prescription and over-service, as reported in other studies [~~23~~26, ~~24~~27]. Although income loss and time loss have not affected every tuberculosis patient interviewed, a considerable number of patients reported being negatively influenced by the disease and concomitant income and time loss. About 37% (190/514) of the patients were

unemployed or suspended from work after contracting TB and had no income, while the others remained employed and experienced income loss due to monthly doctor visits. Only 36% of patients had insurance through the workplace. Patients reported that only a small fraction of medical costs could be reimbursed, and if they became unemployed, their insurance could lapse. Fifty-nine percent of patients had no insurance. Thus, departure from daily money-earning activities had a negative economic impact on patients with TB, resulting in impoverishment and worsening their living situations [~~20~~23]. However, the opportunity costs in these cases may be underestimated, since the value of time loss due to daily drug intake was not calculated, and some patients reported that they had no clear concept of their reduced income due to absences from the workplace.

Out-of-pocket costs and income loss pre-diagnosis were much lower compared with those post-diagnosis, accounting for less than 10% of total costs. This is consistent with reports from other studies [~~25~~28, ~~26~~29]. A review of the financial burden of tuberculosis indicated that half of total costs were incurred before treatment [~~27~~30]. The possible reason for this may be that individuals found it difficult to comprehend the concept of calculating the monetary value of unpaid work, and many of the patients in this study were found as a result of the health check-ups required by their work units. Those suspected patients were initially referred to the CSC for diagnosis, and the first X-ray and sputum smear test were free for them. The delay of diagnosis and number of times visiting health-care facilities were decreased and less costs incurred. We find that those who accepted higher education and occupied in national civil servant/services or retired staff may incur more costs before diagnosis. Those patients may have a good economic condition and care more about self-health ~~conditions~~conditions.

Less than one-fifth of patients initially visited a public health-care facility at the onset of TB symptoms. This led to long delays in the identification of a treatment-seeking pathway before a proper diagnosis was confirmed. The reasons for this may be gender, education, and economic conditions in terms of patients who missed the perfect treatment period, resulting in an advanced disease [~~28~~31]. Financial considerations are a major factor contributing to health-seeking behavior in the Chinese population [~~29~~32]. Inefficiencies in public health facilities and control services at private facilities are the two main constraints responsible for prolonged delay and increased costs of TB diagnosis [~~30~~33, ~~31~~34]. In our analysis, we found visiting times as one of the factors affecting related ~~direct~~ pre-~~dignosis~~diagnosis costs, with a mean of 3 visits to different health-care facilities. Only 35.6% of patients reported that they were diagnosed with suspected tuberculosis at their first visit, and the rest were informed that they had pneumonia, pleurisy, a cold, and so on. Consistent with our study, other investigators pointed out that early diagnosis of tuberculosis reduced costs while achieving treatment success [~~32~~35, ~~33~~36]. Given that TB case detection under the current DOTS strategy in China is through passive case findings, it is imperative to increase patient and community health education and, further, to strengthen the consciousness and capacity of grass-roots public facilities to transfer suspected TB patients to better-equipped hospitals through improved work motivation.

Treatment interruption is defined as any interruption of treatment for at least one day but for < 8 consecutive weeks [~~34~~37], which has been proven to be a risk factor in treatment failure, drug resistance, relapse, and death [~~35~~38, ~~36~~39]. Economic disadvantage is strongly associated with non-adherence to TB treatment [~~37~~40]. Meanwhile, the link between medical costs and adherence in China has been proven [~~38~~41]. In our ~~this~~ study, we didn't ~~found~~find ~~–~~that this ~~was also a risk factor~~ related to high treatment costs statistically. But we find that above seventy percent of post-diagnosis costs occurred during consolidation period. This may be a economic factor influencing the adherence of treatment. Thirteen percent of patients reported that they had interrupted treatment for more than two months, mainly due to: first, side-effects from drugs, when patients experienced a heavy adverse reaction such as liver damage, especially at the intensive period, which always required doctors to stop treating to

protect the liver; second, when patients returned to their home towns, where current strategies prevented them from taking anti-TB drugs beyond one month, so many chose to stop treatment; and finally, more than 90% of the patients who discontinued treatment did so during the continuation phase of treatment, reportedly because, during this phase, most symptoms disappeared and patients erroneously believed that they were cured, making them reluctant to bear the extra burden of the medical costs and income loss. High treatment costs may deter patients who have the most income loss from continuing treatment [~~39~~42]. The patients who stopped treatment usually developed advanced disease difficult to cure, resulting in a longer period of cure and, in turn, higher costs. This seems to be a vicious cycle whereby expenditure~~s~~ increase while interruption leads to more expenditures. Other factors, such as inconvenient daily drug intake and work obligations, were also taken into consideration by the patients.

Although gender was associated with higher costs in some studies [~~40~~43], we found no difference in costs between male and female patients, consistent with results from another study [~~41~~44]. Re-treated patients and ~~those with severe symptoms~~sputum smear positive patients may have higher expenditures during different treatment episodes due to a longer treatment period and more complicated conditions.

This study may seem to be only partly representative of Shenzhen City, since only one district was included. However, the study is representative of most tuberculosis patients in Shenzhen City, because about 50% of all cases under DOTS came from the study site. The re-treatment rate in our study was 5.84%, which is similar to that reported in other studies, with a re-treatment rate of 6.7% in Shenzhen [~~42~~45] and 6.4% in other provinces in China [~~43~~46]. This indicates that our study was acceptably representative of the treatment conditions for TB patients in Shenzhen specifically and China generally. As other studies related to the household costs of tuberculosis, this study also relied on self-reported costs of pre-diagnosis and household income. Thus, recall and reporting bias could not be avoided~~,~~. ~~even in this longitudinal design.~~ Hence, we conducted the first interviews of patients after one month of treatment ~~within two months of their starting treatment,~~ to reduce recall time. Interviewers were also trained to reduce the bias by logic-checking the information from the registration system and recognizing unusually high costs for specific items. In addition, asking patients to calculate the monetary value of unpaid work proved to be difficult, even though our interviewers explained as much as they could.

Our study does have some limitations. First, our study only investigated the economic burden of patients brought by TB and those with severe co-morbidities such as AIDS, diabetes, and lung cancer and older than 59 were excluded. Therefore our study may reflect the economic burden faced by patients result from TB instead of all population and all kinds of diseases. Second, in our study, we did not survey the information regarding companion costs although it may not have much impact on the results since most TB patients in China did not need to be companion during the treatment. Third, we collected the range of the household's pre-TB annual income of every patient instead the exact number. As a result, we are not able to conduct the analysis regarding catastrophic cost.

5. Conclusions

In conclusion, patients and their households face heavy expenditures due to the costs of TB care, which accounted for 26% of patients' annual income. Costs were not equally spread over time, but in the post-diagnosis period, the costs are highest, exacerbating the problem of affordability. Free TB drugs are not the same as free treatment, and non-TB drugs, examinations, and loss of income are important considerations for patients. The income loss among all patients averaged 157USD (IQR 57-855). More visits to health-care facilities generated more out-of-pocket costs. Patients who had severe symptoms and re-treated patients bore a heavier burden. For high costs to be avoided, more attention should be paid to

TB patients, even under the DOTS strategy. TB patients require testing methods with higher sensitivity, higher specificity, and lower costs to reduce their need for visits to health-care facilities, along with more instructions to improve their adherence to treatment, leading to lower re-treatment rates.

Acknowledgments: The authors thank the participants who responded to our questionnaires and the TB doctors who participated in the questionnaire survey. We also express our deep gratitude to the staffs of the Bao'an Chronic Disease Prevention and Cure Hospital and the Center for Tuberculosis Control of Guangdong Province.

References

1. Global tuberculosis report 2018. World Health Organization: Geneva, Switzerland, 2018. Licence: CC BY-NC-SA 3.0 IGO.

2. China Tuberculosis Control Collaboration. The effect of tuberculosis control in China [J]. Lancet 2004, 364, 417–422.

3. Yu, X.; Song, H.F.; Feng, Y.J.; Ye, Z.J. Significance of Gene Xpert-MTB/RIF assay in diagnosis of tuberculosis. J. Practical Med. 2019, 35, 985–988.

4. Liu, Y.; Xu, C.H.; Wang, Z.Y.; et al. [A cross-sectional study on economic burden of pulmonary tuberculosis cases from designated tuberculosis hospital]. Zhonghua Liu Xing Bing Xue Za Zhi. 2019, 40, 559–564 [article in Chinese].

5. Chen, S.; Zhang, H.; Pan, Y.; et. al. Are free anti-tuberculosis drugs enough? An empirical study from three cities in China [J]. Infectious Diseases of Poverty. 2015, 4, 47.

6. Stracker, N.; Hanrahan, C.; Mmolawa, L.; et al. Risk factors for catastrophic costs associated with tuberculosis in rural South Africa. Int. J. Tuberc. Lung Dis. 2019, 23, 756–763.

7. Li, Y.; Ehiri, J.; Tang, S.; et al. Factors associated with patient, and diagnostic delays in Chinese TB patients: a systematic review and meta-analysis. BMC Med. 2013, 11, 156.

8. Qiu, S.; Pan, H.; Zhang, S.; et al. Is tuberculosis treatment really free in China? A study comparing two areas with different management models. PLoS One. 2015, 10, e0126770.

9. Lönnroth, K.; Raviglione, M. The WHO's new End TB Strategy in the post-2015 era of the Sustainable Development Goals. Trans. R. Soc. Trop. Med. Hyg. 2016, 110, 148–150.

10. Guan, H.Y.; Yang, Y.Z.; Tan, W.G.; et al. Effect evaluation of Shenzhen tuberculosis control and prevention program implementation from 2001 to 2010 [J]. Chin. J. Anti-tuberculosis. 2013, 9, 729–737.

11. Liu, J.H.; Zhang, L.; Lu, T.W.; et al. TB control strategy of the preliminary analysis in Bao'an District [J]. Prog. Mod. Biomed. 2010, 21, 4116–4117.

12. de Cuevas, R.M.; Lawson, L.; Al-Sonboli, N.; et al. Patients direct costs to undergo TB diagnosis. Infect. Dis Poverty. 2016, 5, 24.

13. P. Vatcheva, K. and M. Lee, Multicollinearity in Regression Analyses Conducted in Epidemiologic Studies. Epidemiology: Open Access, 2016. 06(02)

14. Feng, C., et al., Log-transformation and its implications for data analysis. Shanghai Arch Psychiatry, 2014. 26(2): p. 105-9.

15. ZHANG Juanjuan, HU Fangxiang, WANG Yunxia, et al. Epidemiological characteristics of tuberculosis in Bao'an District, Shenzhen, 2008-2015. China Tropical Medicine，February 2018，Vol.18，No.2:p128-130

16. Ukwaja, K.N.; Modebe, O.; Igwenyi, C.; et al. The economic burden of tuberculosis care for patients and households in Africa: a systematic review [J]. Int. J. Tuberc. Lung Dis. 2012, 16, 733–739.

17. Suen, S.C.; Bendavid, E.; Goldhaber-Fiebert, J.D. Cost-effectiveness of improvements in diagnosis and treatment accessibility for tuberculosis control in India. Int. J. Tuberc. Lung Dis. 2015, 19, 1115–1124.

18. Pan, H.Q.; Bele, S.; Feng, Y.; et al. Analysis of the economic burden of diagnosis and treatment of tuberculosis patients in rural China [J]. Int. J. Tuberc. Lung Dis. 2013, 17, 1575–1580.

19. Gospodarevskaya, E.; Tulloch, O.; Bunga, C.; et al. Patient costs during tuberculosis treatment in Bangladesh and Tanzania: the potential of shorter regimens [J]. Int. J. Tuberc. Lung Dis. 2014, 18, 810–817.

20. Hu, D.; Long, Q.; Chen, J.; Wang, X.; Huang, F.; Ji, J.S. Factors influencing hospitalization rates and inpatient cost of patients with tuberculosis in Jiangsu Province, China: an uncontrolled before and after study. Int. J. Environ. Res. Public Health. 2019, 16.

21 Onazi, O.; Gidado, M.; Onazi, M.; et al. Estimating the cost of TB and its social impact on TB patients and their households. Public Health Action. 2015, 5, 127–131.

22. Mauch, V.; Bonsu, F.; Gyapong, M.; et al. Free tuberculosis diagnosis and treatment are not enough: patient cost evidence from three continents [J]. Int. J. Tuberc. Lung Dis. 2013, 17, 381–387.

23. Tanvejsilp, P.; Loeb, M.; Dushoff, J.; Xie, F. Healthcare resource uses and out-of-pocket expenses associated with pulmonary TB treatment in Thailand. Pharmacoecon. Open. 2018, 2, 297–308.

24. Pedrazzoli, D.; Siroka, A.; Boccia, D.; et al. How affordable is TB care? Findings from a nationwide TB patient cost survey in Ghana. Trop. Med. Int. Health. 2018, 23, 870–878.

25. Asres, A.; Jerene, D.; Deressa, W. Pre- and post-diagnosis costs of tuberculosis to patients on Directly Observed Treatment Short course in districts of southwestern Ethiopia: a longitudinal study. J. Health Popul. Nutr. 2018, 37, 15.

~~23~~26. Tang, S.; Wang, L.; Wang, H.; Chin, D.P. Access to and affordability of healthcare for TB patients in China: issues and challenges. Infect. Dis. Poverty. 2016, 5, 10.

~~24~~27. Jia, X.; Chen, J.; Zhang, S.; Dai, B.; Long, Q.; Tang, S. Implementing a "free" tuberculosis (TB) care policy under the integrated model in Jiangsu, China: practices and costs in the real world. Infect. Dis. Poverty. 2016, 5, 2.

~~25~~28. Fuady, A.; Houweling, T.A.J.; Mansyur, M.; Burhan, E.; Richardus, J.H. Effect of financial support on reducing the incidence of catastrophic costs among tuberculosis-affected households in Indonesia: eight simulated scenarios. Infect. Dis. Poverty. 2019, 8, 10.

~~26~~29. Mullerpattan, J.B.; Udwadia, Z.Z.; Banka, R.A.; Ganatra, S.R.; Udwadia, Z.F. Catastrophic costs of treating drug resistant TB patients in a tertiary care hospital in India. Indian J. Tuberc. 2019, 66, 87–91.

~~27~~30. Tanimura, T.; Jaramillo, E.; Weil, D.; Raviglione, M.; Lönnroth, K. Financial burden for tuberculosis patients in low- and middle-income countries: a systematic review. Eur. Respir. J. 2014, 43, 1763–1775.

~~28~~31. Gebreegziabher, S.B.; Bjune, G.A.; Yimer, S.A. Patients' and health system's delays in the diagnosis and treatment of new pulmonary tuberculosis patients in West Gojjam Zone, Northwest Ethiopia: a cross-sectional study. BMC Infect. Dis. 2016, 16, 673.

~~29~~32. Li, Y.; Ehiri, J.; Tang, S.; et al. Factors associated with patient, and diagnostic delays in Chinese TB patients: a systematic review and meta-analysis [J]. BMC Med. 2013, 11, 156.

~~30~~33. Bogale, S.; Diro, E.; Shiferaw, A.M.; Yenit, M.K. Factors associated with the length of delay with tuberculosis diagnosis and treatment among adult tuberculosis patients attending at public health facilities in Gondar town, Northwest, Ethiopia. BMC Infect. Dis. 2017, 17, 145.

~~31~~34. Osei, E.; Akweongo, P.; Binka, F. Factors associated with DELAY in diagnosis among tuberculosis patients in Hohoe Municipality, Ghana. BMC Public Health. 2015, 15, 721.

~~32~~35. Pichenda, K.; Nakamura, K.; Morita, A.; et al. Non-hospital DOT and early diagnosis of tuberculosis reduce costs while achieving treatment success [J]. Int. J. Tuberc. Lung Dis. 2012, 16, 828–834.

~~33~~36. Duan, W.; Zhang, W.; Wu, C.; et al. Extent and determinants of catastrophic health expenditure for tuberculosis care in Chongqing municipality, China: a cross-sectional study. BMJ Open. 2019, 9, e026638.

~~34~~37. Jakubowiak, W.; Bogorodskaya, E.; Borisov, S.; et al. Treatment interruptions and duration associated with default among new patients with tuberculosis in six regions of Russia [J]. Int. J. Infect. Dis. 2009, 13, 362–368.

~~35~~38. Mekonnen, H.S.; Azagew, A.W. Non-adherence to anti-tuberculosis treatment, reasons and associated factors among TB patients attending at Gondar town health centers, Northwest Ethiopia. BMC Res. Notes. 2018, 11, 691.

~~36~~39. de Faria Gomes, N.M.; da Mota Bastos, M.C.; Marins, R.M.; et al. Differences between risk factors associated with tuberculosis treatment abandonment and mortality. Pulm. Med. 2015, 2015, 546106. doi:10.1155/2015/546106.

~~37~~340. Wanyonyi, A.W.; Wanjala, P.M.; Githuku, J.; Oyugi, E.; Kutima, H. Factors associated with interruption of tuberculosis treatment among patients in Nandi County, Kenya 2015. Pan Afr. Med. J. 2017, 28(Suppl 1), 11.

~~38~~41. Long, Q.; Smith, H.; Zhang, T.; et al. Patient medical costs for tuberculosis treatment and impact on adherence in China: a systematic review [J]. BMC Public Health. 2011, 11, 393.

~~39~~42. Gorityala, S.B.; Mateti, U.V.; Konuru, V.; Martha, S. Assessment of treatment interruption among pulmonary tuberculosis patients: a cross-sectional study. J. Pharm. Bioallied Sci. 2015, 7, 226–229.

4043. Ukwaja, K.N.; Alobu, I.; Abimbola, S.; et al. Household catastrophic payments for tuberculosis care in Nigeria: incidence, determinants, and policy implications for universal health coverage [J]. Infect. Dis. Poverty. 2013, 2, 21.

~~41~~44. Zhou, C.; Long, Q.; Chen, J.; et al. Factors that determine catastrophic expenditure for tuberculosis care: a patient survey in China. Infect. Dis. Poverty. 2016, 5, 6.

~~42~~45. Bao, H.; Liu, K.; Wu, Z.; et al. Tuberculosis among full-time teachers in Southeast China, 2005‑2016. Int. J. Environ. Res. Public Health. 2018, 15, 2024. Published 2018 Sep 17. doi:10.3390/ijerph15092024.

~~43~~46. Zhu, L.; Yang, Y.Z.; Guan, H.Y.; et al. Trends in drug-resistant tuberculosis after the implementation of the DOTS strategy in Shenzhen, China, 2000-2013. Int. J. Tuberc. Lung Dis. 2017, 21, 759–765. doi:10.5588/ijtld.16.0759.

Additional Editor Comments (if provided):

Dear Authors,

In addition to the valuable points raised by the two reviewers, i have some comments from my end (some of these may be made by the reviewers as well)

1. Abstract - most were from the migrant population - why use ' most' here while elsewhere the number % have been given?

Thanks for the editor's suggestion. We have replaced 'most' with the exact number '95%' in the abstract, which was mentioned in the line 255.

2. out of pockets expenditure? Please be careful with use of phrases (point made by the reviewer as well). Are you looking at total costs due to TB care or out of pocket expenditure. Overall in your paper, no reference was made to the WHO TB patient cost survey handbook 2016. I see that the study was among patients registered in 2013 and the WHO ptient cost survey booklet was not out then. Hence, it will be nice ot have a table in methods with clear operational definitions used.

We are grateful for your suggestion. In our study, we did not include the cost covered by the medical insurance, hence it is inappropriate to use the term 'out of pockets expenditure'. So we replaced it with 'cost' to make it more accurate. There are many kinds of medical insurance in China, and the patients may not be aware of whether they have certain kind of medical insurance or not, so they did not use the medical insurance to reduce their expenditure. However, in our study, the cost is measurable and comparable, so we revised it throughout this article accordingly.

*According to your advice, we have added the operational definitions in **table 1.***

3. 2.2 Study design title should be renamed as 2.2 Study design and population

Revised, thank you.

4. Anonymized data HAS to be shared and this is ESSENTIAL

Thanks for this suggestion. We have provided the anonymous data as attachment in an .xls document.

5. In the ethics statement, please mention the ethics number and the date

We are grateful for the Reviewer's suggestion. The ethical approval number is 2012-SPH021, date 9/28/ 2012.

6. Line 175-176 (regarding primary outcome) - this line is not clear

Thanks for this suggestion. We revised the definition to make it clear in line 192-193 as follows: The primary outcome variables were the mean (median) expense incurred during illness.

7. Line 191 - shouldn't it be the household's pre-TB annual income?

We appreciate your suggestion. Our study does have the limitation that we collected the range of the household's pre-TB annual income of every patient instead the exact number. However, we collected the exact number of the total annual income of every patient and as a result, we used the indicator to measure the patients' economic burden.

8. Line 204-06. Similar issues were faced by us in another study. Another analysis may be to classify the costs as catastrophic or not and then do a log binomial regression. We can present

both the results. See the following paper for example, as to how the analysis was done: https://www.ncbi.nlm.nih.gov/pubmed/30865730

We appreciate this suggestion. As mentioned above, we did not consider the catastrophic cost as an indicator in the design phase of our study, so we are not able to conduct the analysis regarding catastrophic cost. In our revision, based on the paper given above, we improved our method of statistics by adopting 2 methods in our revised manuscript.

9. Line 206 - Bivariate means two outcomes. All the analyses presented in this paper are univariate which can be further classified into single variable (curde or unadjusted) and multivariable (adjsuted) analysis.

Thanks for this suggestion. We have adopted a new method in the data analysis which is described carefully in line 221-244.

10. What about catastrophic costs, what about inequity in distribution of catastrophic costs. See this paper for example. https://www.ncbi.nlm.nih.gov/pubmed/30173603

We appreciate your suggestion. However, we did not adopt this professional term at the start of the study, and the annual patient income is expressed as a range instead of an exact number. As a result, it would be impossible to calculate the catastrophic costs to patients.

11. All the table and figure title have to be standalone with details about time place and person. Check for footnotes for abbreviations. All tables should be formatted again to make them presentable. For ex n and % in separate columns. n column shuold be right indented, (%) column should be left indented and vertical line between n and % should then be removed. CHeck other tables as well.

We appreciate your suggestion. We have reformatted the tables throughout the article according to your suggestions.

12. Why don't the authors look separately at factors associated with pre-dx costs and post-diagnosis costs.

Thank you for this suggestion. We have analyzed the factors associated with different period of the treatment according to your suggestion and revised the statement in the data analysis, results and discussion section on the basis of the new analysis results.

13. If the authors have outcome data, why not compare unfav out and catastrophic costs, after adjusting for potential confounders.

We appreciate your suggestion. However, we did not adopt this professional term at the start of the study, and the annual patient income is expressed as a range instead of an exact number. As a result, it would be impossible to calculate the catastrophic costs to patients.

Reviewer #1: This work is on an important area namely the economic burden due to Tuberculosis related to the EndTB strategy goal of Zero Catastrophic cost for households due to Tuberculosis. The author have drafted the manuscript well and included several important points needed for a cost analysis study.

The study utilised a follow-up methodology to capture costs during the treatment phase. The authors have also studied some factors related to costs.

While the study justification rightly draws support from the EndTB Goal of Zero Catastrophic Cost, the authors have not included Catastrophic Cost estimation in the paper, despite having all the necessary data for the calculation of the same. It is highly recommended that the authors may include catastrophic cost and accordingly the manuscript may be redrafted. If there are data available on the coping costs or mechanism, they may also be included to understand the complete picture of the economic burden faced by the patients with Tuberculosis.

We appreciate your suggestion. However, we did not adopt this professional term at the start of the study, and the annual patient income is expressed as a range instead of an exact number. As a result, it would be impossible to calculate the catastrophic costs to patients. In our study, most of the study population were factory workers. Only 186 workers knew that they had medical insurance and 305 workers knew that they did not have any medical insurance. Other 23 workers were unaware of whether they had medical insurance or not, and at the end of the study, they did not bring about any medical care reimbursement cost. Only 34 patients provided information about medical care reimbursement cost. As a result, the proportion of cost paid by medical insurance was not included in the analysis.

Abstract

Though the study includes both direct and indirect costs, the abstract starts with the terminology ‘expenditures’ which may apparently mislead a casual reader

Follow-up method utilised may be included in the methods under the abstract

Thank you for this suggestion. We have replaced the word 'expenditures' with 'costs' to avoid misunderstanding and added the follow-up method in the abstract.

Methodology

In the study settings,

the following may be included, which have implications while interpreting of the results:

1.Proportion of Treatment and Retreatment Cases in the District;

Thanks for this suggestion. In the discussion section, we mentioned the proportion of treatment and retreatment cases in line 508-509 as follows: The re-treatment rate in our study was 5.84%, which is similar to that reported in other studies, with a re-treatment rate of 6.7% in Shenzhen and 6.4% in other provinces in China.

2.Actual figures of Incidence and Prevalence of Tuberculosis in Shenzhen Region/Bao’an District (lines 119-120), which needs to be specified seperately further between natives and migrants;

We appreciate your suggestion. We have added the data in line 139-141 as follows: In 2010, the prevalence rate of smear positive of native population was 19.44 per 100 000 population, and that of the migrant population was 59.96 per 100 000 population.

3. No, of TB Cases in the District during the study year among natives and migrants;

Thanks for this suggestion. We have listed the number in line 400-402 as follows: The total number of registered active tuberculosis cases were 1317, included 76(5.8) native case and 1241(94.2%) migrant cases in 2013.

4. No. of (mandatory)visits as per the existing TB program during treatment;

Thank you for this suggestion. We have added the data in line 157.

5.Proportion of patients with TB having co-morbidities namely diabetes, HIV, lung cancer etc

We appreciate your suggestion. Because in our study, the patients with co-morbidities were excluded from the study, it is impossible to calculate the proportion of patients with TB having co-morbidities.

6.Whether Active Case finding strategy is implemented or not;

We appreciate your suggestion. In the study setting section, we have added the statements in line 132-139 to clarify the way of TB patients being found in China as follows: The hospitals were the main place finding tuberculosis case and the Center for Prevention and Cure of chronic diseases (CPC) were the local designated TB facilities in the national TB programme networks, providing treatment and managing the tuberculosis cases. The healthcare providers in general hospitals were obligate to refer the suspected cases to CPC and the basic information of suspected cases were sent to CPC. If the cases did not consulted to CPC in time, the workers of CPC would contact the suspected case. Actively cooperating with medical institutions and TB prevention and control institutions, the discovery rate and report quality was increasing by years.

7.Mean/Median household (and/or Individual) income in the region/district;

Thank you for this suggestion. We have added the median household income in Bao'an district in line 155-157 as follows: The mean household income in the region was 6,656.79 USD of 2012 according to the Statistical yearbook 2013.

8. Proportion of people in different economic categories;

We appreciate your suggestion. However, we did not have access to the detailed information about the income of different population.

9. a brief description of occupational health care provisions/model in the district relevant to TB care;

Thanks for this suggestion. In China, there is no occupational health care provisions/model focus on TB prevention only, the workers in our study were provided with the general occupational health service. For example, the workers may participant in the regular medical examination paid by the factory owner.

10. brief description of the existing intervention in the district to address economic burden faced by patients/households due to TB

We appreciate your suggestion. In line 148-151, we described the existing free policies to relief the economic burden on TB patients: The Center for Prevention and Cure of chronic diseases (CPC) is the institution authorized to provide TB diagnosis, treatment, and monitoring. The CPC provides patients with a radiologic imaging studies (X-ray) while diagnosed, and sputum smear tests during treatment, and anti-TB drugs for 6-8 months for free.

11. whether hospitalisation costs due to TB for persons with TB are covered under the existing program?

Thank you for this suggestion. The hospitalization costs due to TB was not covered under the existing program and born by the patients themselves. We have added the statements in line 148-151 to state the range of free TB policy. Since the cost regarding hospitalization is not included in the policy, it have to be borne by patients themselves.

Study Design:

Why were those aged above 59 years not included for the study? Reasons may be stated and also

included/addressed in the discussion.

We appreciate your suggestion. People ages above 59 are more likely to have comorbidities which may influence the analysis of economic burden, so we excluded them. We have added the reason in line 166-167 as follows: The patients aged above 59 were excluded considering the elderly may have more commodities and those with MDR-TB were excluded too.

In the discussion, need to consider, how non-inclusion of those with morbidity namely Diabetes, HIV affected the representativeness of the study. HIV and Diabetes have programmatic implications for Tuberculosis control as well as have been shown by studies in the literature to drive the costs due to tuberculosis higher.

Thanks for this suggestion. We have discussed the influence of non-inclusion on the representativeness of our study in the limitation section in line 392-398 as follows: We included 533 eligible patients in the survey, 19 of which were lost to follow-up. The average age of them was 29.6±10.2years. The male was 14 and female was 5. One patient was with a severe symptoms appeared hemoptysis. The others were with mild or moderate symptoms like cough, chest distress and fever etc. the lost to follow-up was about 4 percent, and the general Socio-demographic and clinical characteristics of those 19 participants lost to follow-up were identical with the 514 patients. So， we considered there no much influence on our conclusion.

However, the exclusion can inevitably injure the representativeness, so we added this point as a limitation of our study in line 519-523 as follows: Our study only investigated the economic burden of patients brought by TB and those with severe co-morbidities such as AIDS, diabetes, and lung cancer were excluded. Therefore our study may reflect the economic burden faced by TB patients instead of all population.

Where those with MDR-TB excluded? Or is it that there were no drug resistant cases during the study period, which is quite unlikely. The authors have not stated whether the study is about costs due to drug sensitive tuberculosis. These needs to be clarified in title, abstract, justification (objectives), methods, and discussion.

Thank you for this suggestion. We have added the statement in the Study Design and population part in line165-167. To illustrate the study population of our survey more accurate, we have added the particular explanation in every section accordingly.

In our study, there were only 4 patients with MDR-TB among total 514 patients. One patient failed to achieve TB suppression in the third month of treatment, and the other three patients developed drug resistance during the treatment. Doctors adjusted the medicine according to their condition and part of second-line medicine is not free. Because there were few patients with MDR-TB, we did not eliminated them from the analysis. However, they may not have much impact on the stability of the analysis.

Sample Size has not been estimated by the authors. Studies in the literature on Cost of illness have adopted different methods to calculate sample size. They may be referred to.

We appreciate your suggestion. Ahead of our study, we did not calculate the sample size we needed. We included every patient who met the inclusion criteria and agreed to take part in our survey during the study period. Analysis results showed that the sample size of our study is enough to achieve the power required.

The cost data was based on self-reporting. The authors have mentioned both in the methodology and strengths in discussion, that that a logic-check was done. Brief description of this may be

provided to help readers understand how this enabled better data quality.

We appreciate your suggestion. In the Data collection section, we have explained the process of a logic-check in line 187-189 as follows: Those contradictions or missing information would be asked of patients again in a subsequent interview. We also evaluated the quality of collected data by verifying the routine health information system.

Timing of the first interview in the intensive phase could have affected the recall leading to differential recall among different participants. The data on delay between start of treatment and interview may be provided as mean and range. Similarly, it would be important to state whether the timing of multiple interviews were standardised for all patients.

Thank you for this suggestion. First interview is in the first month of intensive phase treatment. We have added the median delay time of diagnosis in line 270-271. In the limitation section, we have mentioned that recall bias could not be avoided in our study as it relied on the information self-reported by patients. Patients went to see the doctor in chronic disease station nearby on time and were followed-up during their visit.

Methodology can include explicitly the perspective from which cost was calculated, though it is understandable from the objective.

Thanks for this suggestion. We have added the illustration in the line 192 as follows: We analysis the economic burden from tuberculosis patients' perspective.

The reason for non-inclusion of guardian (companion) cost is not acceptable as the need for guardian are often higher during pre-diagnosis phase as the patients have more illness and may often make multiple visits to one or more provider before the diagnosis and start of treatment. Authors have also reported hospitalisations during pre-diagnosis period. Guardian (companion) costs needs to be included in the analysis.

We appreciate your suggestion. We regret mentioning that the companion costs was not surveyed in the study. In China, TB is a common disease and most patients do not need companions during the treatment. Another reason is that patients in our study were surveyed in the community or chronic disease station near their home within 15 minutes' walk. So the companion costs during pre-diagnosis period may not have much impact on the outcome. We admit that it is a limitation of our study and have added the explanation in line 523-525 as follows: Second, in our study, we did not survey the information regarding companion costs although it may not have much impact on the results since most TB patients in China did not need to be companion during the treatment.

The source of questionnaire may be stated and provided.

Thanks for your suggestion. In this survey, we combined the aim of our study with standardized questionnaire used to investigate the demographic characteristic and designed the questionnaire. We have provided the questionnaire we used as attachment.

Though the study reported insurance coverage for participants, authors have not stated how reimbursements were adjusted against the costs in the analysis.

Thanks for your suggestion. In our study, most of the study population were factory workers. Only 186 workers knew that they had medical insurance and 305 workers knew that they did not have any medical insurance. Other 23 workers were unaware of whether they had medical insurance or not, and at the end of the study, they did not bring about any medical care reimbursement cost. Only 34 patients provided information about medical care reimbursement cost. As a result, the proportion of cost paid by medical insurance was not

included in the analysis.

Results

Clinical characteristics including co-morbidities, pulmonary/extrapulmonary, etc to be included.

Care-seeking characteristics should include number of consultations/visits prior to diagnosis, number of providers visited and type of providers / facility visited (especially private providers /facility as it can vary from single doctor provider facility to corporate hospital)

We appreciate your suggestion. In the Socio-demographic Characteristics and Care-seeking Behaviors section, we have added the number of consultations prior diagnosis and type of facility visited in line 267-270. However, we did not collect information about the number of providers visited.

Data on whether the participant was a beneficiary of any of the existing interventions in the district to reduce the economic burden to patients due to TB, needs to be reported, including the quantum of benefit

Thanks for your suggestion. We have mentioned the coverage of the existing free TB policy in the Study setting section in line 149-151. In the second paragraph, we have also added the description of the extent of free treatment and the cost covered by the policy in line 404-406 as follows: Under the DOTS program, the CPC provide tuberculosis patients with a radiologic imaging studies (X-ray) , sputum smear tests and anti-TB drug during treatment for free amounting to 102.07 USD.

The cost during different phases of treatment could also be made available especially since authors have conducted multiple interviews through follow-up to obtain costs through the treatment period. They are important while planning interventions

Where there no hospitalisation episodes among participants during the treatment phase?

We appreciate your suggestion. Our study only investigated the cost reported by the patients occurred in the chronic disease station. There was no inpatient ward in the chronic disease station, so we did not surveyed the information about hospitalization.

In Table 2, what are these Non-Tb drugs?

Thank you for this suggestion. We have added the subsidiary drugs in line 152-153.

In table 3, what timeline does sputum smear test pertain to? What is the definition of household economic burden? What is the definition for degree of symptoms at diagnosis? Is it self-reported or based on clinical assessment? The Operational definition of these variables need to be included in the methodology.

Thanks for this suggestion. We have illustrated these definitions clearly in ***table 1****.*

Why insurance coverage was not included as a factor for economic burden?

Thanks for your suggestion. In our study, most of the study population were factory workers. Only 186 workers knew that they had medical insurance and 305 workers knew that they did not have any medical insurance. Other 23 workers were unaware of whether they had medical insurance or not, and at the end of the study, they did not bring about any medical care reimbursement cost. Only 34 patients provided information about medical care reimbursement cost. As a result, the proportion of cost paid by medical insurance was not included in the analysis.

Among the factors considered for economic burden, some of them pertain to pre-diagnosis costs and other may pertain to treatment phase costs and few many be for total costs. It is recommended that these be split accordingly instead of considering everything together, as there are implications for suggesting possible interventions to reduce cost.

We appreciate this suggestion. Considering everything together is truly not legible and easy to guide practice. So we decided to analyze the factors associated with pre-diagnosis and post-diagnosis separately according to your suggestion.

Discussion

While the population of the district has 65% migrants, in the study, 95% of the study participants are migrants. This can have implications for representativeness of the study. This needs to be discussed in detail; this again importance further since it is one of the factors found to be significantly associated with economic burden as in table 3.

Thanks for this suggestion. We have discussed it carefully in line 402-405 as follows: As one of the most population of Shenzhen City and owing numbers of factories , the migrant population in Bao'an district always exceeds the average level of Shenzhen City. The total number of registered active tuberculosis cases were 1317, included 76(5.8%) native case and 1241(94.2%) migrant cases in 2013. In our study, 95 percent were migrant cases, so our study can reflect the TB condition of Bao'an district truly.

Socio-demographic and clinical characteristics of those 19 participants lost to follow-up need to be reported and the implications of the study results need to be discussed

We appreciate this suggestion. In line 395-401, we have added the statement of the influence of lost follow-ups on our outcome.

Of the 533 participants, 176 patients were reported to have been shifted to CPC with no costs for patients during pre-diagnosis period. Can description of these transfers provided and included in study setting. How this lead to no delays and no costs are important for understanding the drivers of costs/economic burden.

Thank you for this suggestion. We have added the detailed illustration in the study setting section in line 132-139.

Line 413, what are the author establishing by stating ‘recall and reporting bias could not be avoided, even in this longitudinal design’ The statement is self-contradictory. May need to be revised

Revised, thank you.

Figure may not be needed as this information is represented in the table 2 and is clearly evident too.

We appreciate this suggestion. We deleted figure 1 in the article.

Some Typographical Errors

Alignment of 70% in Age category 40-50 in Table 1

Subtitle Insurance to be made bold in Table 1

Weblink in line 112 may be shifted to reference and reference number provided

Title of Table 3, CNY needs to be removed

Revised, thank you.

Reviewer #2: 1. The author needs to add some more details on opportunity cost i.e, income loss by patients due to illness. Some patients were hospitalized but they have earnings from job

without wage loss then how to quantify the opportunity cost? Also, variation in salary is very heterogenetic. The author needs to describe about opportunity cost.

*We appreciate this suggestion. We have describe the opportunity cost in **table 1** carefully. If TB patients still worked during the hospitalization, then there was no wage loss and the wage loss was 0.*

2. Under background characteristics, author mentioned about insurance company, but for Out of Pocket Expenditure (OOPE), we should not mention reimbursements given by insurance company. If the patients are getting reimbursements from any type of insurance, it should be deducted from the total out of pocket expenditure.

Net out of pocket expenditure = Total expenditure - Reimbursement.

Reimbursement amount should be highlighted if possible.

Thank you for this suggestion. We realized that it was inappropriate to use the term 'out of pocket expenditure' to describe the economic burden for in our study, the cost covered by medical insurance is not included in the analysis. In our study, most of the study population were factory workers. Only 186 workers knew that they had medical insurance and 305 workers knew that they did not have any medical insurance. Other 23 workers were unaware of whether they had medical insurance or not, and at the end of the study, they did not bring about any medical care reimbursement cost. Only 34 patients provided information about medical care reimbursement cost. As a result, the proportion of cost paid by medical insurance was not included in the analysis.

3. In methodology section, the author has mentioned the Chi-square test and Mann-Whitney analysis to examine the effect of each predictor variable on economic burden. But Chi-square test is used to know the association between predictor variables and regression analysis is used to understand the effects of predictor variables.

We appreciate your suggestion. We have revised the analysis method and Chi-square test is not used this time. So we deleted it.

4. Table 3 analysis indicated that factors related to direct and opportunity costs (CNY) and in relation with the background variables. The heading should be association or differentials by background characteristics of patients and table 4 is showing the factor with regression analysis.

We have revised the table titles according to your advice and thanks for your advice.

5. In table 4 multivariate analysis, only few predictor variables have been added. However, the author described in methodology section A forward stepwise approach was used to find the appropriate model. If P-value less than 0.05 is significant. It is good and recognised methods for adding the variables in regression analysis. Try also Variance inflation factor (VIF) to know the multicollinearity in predictor variables. If VIF less than 10, than the author can add the predictor variables and explore more variable as predictor variables.

Thank you for this suggestion. We have adjusted the data analysis method according to your suggestion.

Based on the above observations and looking into overall merit of the paper, few modifications in line of above points may be considered before sending it for publication.
