# Supplementary material for: Analysis of the economic burden of diagnosis and treatment on patients with tuberculosis in Bao’an district of Shenzhen City, China
Source: PLoS One. 2020 Aug 31;15(8):e0237865. doi: 10.1371/journal.pone.0237865 (PMC7458315; doi:10.1371/journal.pone.0237865)
Supplement: Attachment — Submitted filename: Response to editors and reviewers.docx [file pone.0237865.s006.docx]

Additional Editor Comments (if provided):

Dear Authors,

In addition to the valuable points raised by the two reviewers, i have some comments from my end (some of these may be made by the reviewers as well)

1. Abstract - most were from the migrant population - why use ' most' here while elsewhere the number % have been given?

*Thanks for the editor’s suggestion. We have replaced ‘most’ with the exact number ‘95%’ in the abstract, which was mentioned in the line 255.*

2. out of pockets expenditure? Please be careful with use of phrases (point made by the reviewer as well). Are you looking at total costs due to TB care or out of pocket expenditure. Overall in your paper, no reference was made to the WHO TB patient cost survey handbook 2016. I see that the study was among patients registered in 2013 and the WHO ptient cost survey booklet was not out then. Hence, it will be nice ot have a table in methods with clear operational definitions used.

*We are grateful for your suggestion. In our study, we did not include the cost covered by the medical insurance, hence it is inappropriate to use the term ‘out of pockets expenditure’. So we replaced it with ‘cost’ to make it more accurate. There are many kinds of medical insurance in China, and the patients may not be aware of whether they have certain kind of medical insurance or not, so they did not use the medical insurance to reduce their expenditure. However, in our study, the cost is measurable and comparable, so we revised it throughout this article accordingly.*

*According to your advice, we have added the operational definitions in* ***table 1.***

3. 2.2 Study design title should be renamed as 2.2 Study design and population

*Revised, thank you.*

4. Anonymized data HAS to be shared and this is ESSENTIAL

*Thanks for this suggestion. We have provided the anonymous data as attachment in an .xls document.*

5. In the ethics statement, please mention the ethics number and the date

*We are grateful for the Reviewer’s suggestion.* *The ethical approval number is 2012-SPH021, date 9/28/ 2012.*

6. Line 175-176 (regarding primary outcome) - this line is not clear

*Thanks for this suggestion. We revised the definition to make it clear in line 192-193 as follows: The primary outcome variables were the mean (median) expense incurred during illness.*

7. Line 191 - shouldn't it be the household's pre-TB annual income?

*We appreciate your suggestion. Our study does have the limitation that we collected the range of the household's pre-TB annual income of every patient instead the exact number. However, we collected the exact number of the total annual income of every patient and as a result, we used the indicator to measure the patients’ economic burden.*

8. Line 204-06. Similar issues were faced by us in another study. Another analysis may be to classify the costs as catastrophic or not and then do a log binomial regression. We can present both the results. See the following paper for example, as to how the analysis was done: <https://www.ncbi.nlm.nih.gov/pubmed/30865730>

*We appreciate this suggestion. As mentioned above, we did not consider the catastrophic cost as an indicator in the design phase of our study, so we are not able to conduct the analysis regarding catastrophic cost. In our revision, based on the paper given above, we improved our method of statistics by adopting 2 methods in our revised manuscript.*

9. Line 206 - Bivariate means two outcomes. All the analyses presented in this paper are univariate which can be further classified into single variable (curde or unadjusted) and multivariable (adjsuted) analysis.

*Thanks for this suggestion. We have adopted a new method in the data analysis which is described carefully in line 221-244.*

10. What about catastrophic costs, what about inequity in distribution of catastrophic costs. See this paper for example. <https://www.ncbi.nlm.nih.gov/pubmed/30173603>

*We appreciate your suggestion. However, we did not adopt this professional term at the start of the study, and the annual patient income is expressed as a range instead of an exact number. As a result, it would be impossible to calculate the catastrophic costs to patients.*

11. All the table and figure title have to be standalone with details about time place and person. Check for footnotes for abbreviations. All tables should be formatted again to make them presentable. For ex n and % in separate columns. n column shuold be right indented, (%) column should be left indented and vertical line between n and % should then be removed. CHeck other tables as well.

*We appreciate your suggestion. We have reformatted the tables throughout the article according to your suggestions.*

12. Why don't the authors look separately at factors associated with pre-dx costs and post-diagnosis costs.

*Thank you for this suggestion. We have analyzed the factors associated with different period of the treatment according to your suggestion and revised the statement in the data analysis, results and discussion section on the basis of the new analysis results.*

13. If the authors have outcome data, why not compare unfav out and catastrophic costs, after adjusting for potential confounders.

*We appreciate your suggestion. However, we did not adopt this professional term at the start of the study, and the annual patient income is expressed as a range instead of an exact number. As a result, it would be impossible to calculate the catastrophic costs to patients.*

Reviewer #1: This work is on an important area namely the economic burden due to Tuberculosis related to the EndTB strategy goal of Zero Catastrophic cost for households due to Tuberculosis. The author have drafted the manuscript well and included several important points needed for a cost analysis study.

The study utilised a follow-up methodology to capture costs during the treatment phase. The authors have also studied some factors related to costs.

While the study justification rightly draws support from the EndTB Goal of Zero Catastrophic Cost, the authors have not included Catastrophic Cost estimation in the paper, despite having all the necessary data for the calculation of the same. It is highly recommended that the authors may include catastrophic cost and accordingly the manuscript may be redrafted. If there are data available on the coping costs or mechanism, they may also be included to understand the complete picture of the economic burden faced by the patients with Tuberculosis.

*We appreciate your suggestion. However, we did not adopt this professional term at the start of the study, and the annual patient income is expressed as a range instead of an exact number. As a result, it would be impossible to calculate the catastrophic costs to patients.*

*In our study, most of the study population were factory workers. Only 186 workers knew that they had medical insurance and 305 workers knew that they did not have any medical insurance. Other 23 workers were unaware of whether they had medical insurance or not, and at the end of the study, they did not bring about any medical care reimbursement cost. Only 34 patients provided information about medical care reimbursement cost. As a result, the proportion of cost paid by medical insurance was not included in the analysis.*

Abstract

Though the study includes both direct and indirect costs, the abstract starts with the terminology ‘expenditures’ which may apparently mislead a casual reader

Follow-up method utilised may be included in the methods under the abstract

*Thank you for this suggestion. We have replaced the word ‘expenditures’ with ‘costs’ to avoid misunderstanding and added the follow-up method in the abstract.*

Methodology

In the study settings,

the following may be included, which have implications while interpreting of the results:

1.Proportion of Treatment and Retreatment Cases in the District;

*Thanks for this suggestion. In the discussion section, we mentioned the proportion of treatment and retreatment cases in line 508-509 as follows: The re-treatment rate in our study was 5.84%, which is similar to that reported in other studies, with a re-treatment rate of 6.7% in Shenzhen and 6.4% in other provinces in China.*

2.Actual figures of Incidence and Prevalence of Tuberculosis in Shenzhen Region/Bao’an District (lines 119-120), which needs to be specified seperately further between natives and migrants;

*We appreciate your suggestion. We have added the data in line 139-141 as follows: In 2010, the prevalence rate of smear positive of native population was 19.44 per 100 000 population, and that of the migrant population was 59.96 per 100 000 population.*

3. No, of TB Cases in the District during the study year among natives and migrants;

*Thanks for this suggestion. We have listed the number in line 400-402 as follows: The total number of registered active tuberculosis cases were 1317, included 76(5.8) native case and 1241(94.2%) migrant cases in 2013.*

4. No. of (mandatory)visits as per the existing TB program during treatment;

*Thank you for this suggestion. We have added the data in line 157.*

5.Proportion of patients with TB having co-morbidities namely diabetes, HIV, lung cancer etc

*We appreciate your suggestion. Because in our study, the patients with co-morbidities were excluded from the study, it is impossible to calculate the proportion of patients with TB having co-morbidities.*

6.Whether Active Case finding strategy is implemented or not;

*We appreciate your suggestion. In the study setting section, we have added the statements in line 132-139 to clarify the way of TB patients being found in China as follows: The hospitals were the main place finding tuberculosis case and the Center for Prevention and Cure of chronic diseases (CPC) were the local designated TB facilities in the national TB programme networks, providing treatment and managing the tuberculosis cases. The healthcare providers in general hospitals were obligate to refer the suspected cases to CPC and the basic information of suspected cases were sent to CPC. If the cases did not consulted to CPC in time, the workers of CPC would contact the suspected case. Actively cooperating with medical institutions and TB prevention and control institutions, the discovery rate and report quality was increasing by years.*

7.Mean/Median household (and/or Individual) income in the region/district;

*Thank you for this suggestion. We have added the median household income in Bao’an district in line 155-157 as follows: The mean household income in the region was 6,656.79 USD of 2012 according to the Statistical yearbook 2013.*

8. Proportion of people in different economic categories;

*We appreciate your suggestion. However, we did not have access to the detailed information about the income of different population.*

9. a brief description of occupational health care provisions/model in the district relevant to TB care;

*Thanks for this suggestion. In China, there is no occupational health care provisions/model focus on TB prevention only, the workers in our study were provided with the general occupational health service. For example, the workers may participant in the regular medical examination paid by the factory owner.*

10. brief description of the existing intervention in the district to address economic burden faced by patients/households due to TB

*We appreciate your suggestion. In line 148-151, we described the existing free policies to relief the economic burden on TB patients: The Center for Prevention and Cure of chronic diseases (CPC) is the institution authorized to provide TB diagnosis, treatment, and monitoring. The CPC provides patients with a radiologic imaging studies (X-ray) while diagnosed, and sputum smear tests during treatment, and anti-TB drugs for 6-8 months for free.*

11. whether hospitalisation costs due to TB for persons with TB are covered under the existing program?

*Thank you for this suggestion. The hospitalization costs due to TB was not covered under the existing program and born by the patients themselves. We have added the statements in line 148-151 to state the range of free TB policy. Since the cost regarding hospitalization is not included in the policy, it have to be borne by patients themselves.*

Study Design:

Why were those aged above 59 years not included for the study? Reasons may be stated and also included/addressed in the discussion.

*We appreciate your suggestion. People ages above 59 are more likely to have comorbidities which may influence the analysis of economic burden, so we excluded them. We have added the reason in line 166-167 as follows: The patients aged above 59 were excluded considering the elderly may have more commodities and those with MDR-TB were excluded too.*

In the discussion, need to consider, how non-inclusion of those with morbidity namely Diabetes, HIV affected the representativeness of the study. HIV and Diabetes have programmatic implications for Tuberculosis control as well as have been shown by studies in the literature to drive the costs due to tuberculosis higher.

*Thanks for this suggestion. We have discussed the influence of non-inclusion on the representativeness of our study in the limitation section in line 392-398 as follows: We included 533 eligible patients in the survey, 19 of which were lost to follow-up. The average age of them was 29.6±10.2years. The male was 14 and female was 5. One patient was with a severe symptoms appeared hemoptysis. The others were with mild or moderate symptoms like cough, chest distress and fever etc. the lost to follow-up was about 4 percent, and the general Socio-demographic and clinical characteristics of those 19 participants lost to follow-up were identical with the 514 patients. So，we considered there no much influence on our conclusion.*

*However, the exclusion can inevitably injure the representativeness, so we added this point as a limitation of our study in line 519-523 as follows: Our study only investigated the economic burden of patients brought by TB and those with severe co-morbidities such as AIDS, diabetes, and lung cancer were excluded. Therefore our study may reflect the economic burden faced by TB patients instead of all population.*

Where those with MDR-TB excluded? Or is it that there were no drug resistant cases during the study period, which is quite unlikely. The authors have not stated whether the study is about costs due to drug sensitive tuberculosis. These needs to be clarified in title, abstract, justification (objectives), methods, and discussion.

*Thank you for this suggestion. We have added the statement in the Study Design and population part in line165-167. To illustrate the study population of our survey more accurate, we have added the particular explanation in every section accordingly.*

*In our study, there were only 4 patients with MDR-TB among total 514 patients. One patient failed to achieve TB suppression in the third month of treatment, and the other three patients developed drug resistance during the treatment. Doctors adjusted the medicine according to their condition and part of second-line medicine is not free. Because there were few patients with MDR-TB, we did not eliminated them from the analysis. However, they may not have much impact on the stability of the analysis.*

Sample Size has not been estimated by the authors. Studies in the literature on Cost of illness have adopted different methods to calculate sample size. They may be referred to.

*We appreciate your suggestion. Ahead of our study, we did not calculate the sample size we needed. We included every patient who met the inclusion criteria and agreed to take part in our survey during the study period. Analysis results showed that the sample size of our study is enough to achieve the power required.*

The cost data was based on self-reporting. The authors have mentioned both in the methodology and strengths in discussion, that that a logic-check was done. Brief description of this may be provided to help readers understand how this enabled better data quality.

*We appreciate your suggestion. In the Data collection section, we have explained the process of a logic-check in line 187-189 as follows: Those contradictions or missing information would be asked of patients again in a subsequent interview. We also evaluated the quality of collected data by verifying the routine health information system.*

Timing of the first interview in the intensive phase could have affected the recall leading to differential recall among different participants. The data on delay between start of treatment and interview may be provided as mean and range. Similarly, it would be important to state whether the timing of multiple interviews were standardised for all patients.

*Thank you for this suggestion. First interview is in the first month of intensive phase treatment. We have added the median delay time of diagnosis in line 270-271. In the limitation section, we have mentioned that recall bias could not be avoided in our study as it relied on the information self-reported by patients. Patients went to see the doctor in chronic disease station nearby on time and were followed-up during their visit.*

Methodology can include explicitly the perspective from which cost was calculated, though it is understandable from the objective.

*Thanks for this suggestion. We have added the illustration in the line 192 as follows: We analysis the economic burden from tuberculosis patients’ perspective.*

The reason for non-inclusion of guardian (companion) cost is not acceptable as the need for guardian are often higher during pre-diagnosis phase as the patients have more illness and may often make multiple visits to one or more provider before the diagnosis and start of treatment. Authors have also reported hospitalisations during pre-diagnosis period. Guardian (companion) costs needs to be included in the analysis.

*We appreciate your suggestion. We regret mentioning that the companion costs was not surveyed in the study. In China, TB is a common disease and most patients do not need companions during the treatment. Another reason is that patients in our study were surveyed in the community or chronic disease station near their home within 15 minutes’ walk. So the companion costs during pre-diagnosis period may not have much impact on the outcome. We admit that it is a limitation of our study and have added the explanation in line 523-525 as follows: Second, in our study, we did not survey the information regarding companion costs although it may not have much impact on the results since most TB patients in China did not need to be companion during the treatment.*

The source of questionnaire may be stated and provided.

*Thanks for your suggestion. In this survey, we combined the aim of our study with standardized questionnaire used to investigate the demographic characteristic and designed the questionnaire. We have provided the questionnaire we used as attachment.*

Though the study reported insurance coverage for participants, authors have not stated how reimbursements were adjusted against the costs in the analysis.

*Thanks for your suggestion. In our study, most of the study population were factory workers. Only 186 workers knew that they had medical insurance and 305 workers knew that they did not have any medical insurance. Other 23 workers were unaware of whether they had medical insurance or not, and at the end of the study, they did not bring about any medical care reimbursement cost. Only 34 patients provided information about medical care reimbursement cost. As a result, the proportion of cost paid by medical insurance was not included in the analysis.*

Results

Clinical characteristics including co-morbidities, pulmonary/extrapulmonary, etc to be included.

Care-seeking characteristics should include number of consultations/visits prior to diagnosis, number of providers visited and type of providers / facility visited (especially private providers /facility as it can vary from single doctor provider facility to corporate hospital)

*We appreciate your suggestion. In the Socio-demographic Characteristics and Care-seeking Behaviors section, we have added the number of consultations prior diagnosis and type of facility visited in line 267-270. However, we did not collect information about the number of providers visited.*

Data on whether the participant was a beneficiary of any of the existing interventions in the district to reduce the economic burden to patients due to TB, needs to be reported, including the quantum of benefit

*Thanks for your suggestion. We have mentioned the coverage of the existing free TB policy in the Study setting section in line 149-151. In the second paragraph, we have also added the description of the extent of free treatment and the cost covered by the policy in line 404-406 as follows: Under the DOTS program, the CPC provide tuberculosis patients with a radiologic imaging studies (X-ray) , sputum smear tests and anti-TB drug during treatment for free amounting to 102.07 USD.*

The cost during different phases of treatment could also be made available especially since authors have conducted multiple interviews through follow-up to obtain costs through the treatment period. They are important while planning interventions

Where there no hospitalisation episodes among participants during the treatment phase?

*We appreciate your suggestion. Our study only investigated the cost reported by the patients occurred in the chronic disease station. There was no inpatient ward in the chronic disease station, so we did not surveyed the information about hospitalization.*

In Table 2, what are these Non-Tb drugs?

*Thank you for this suggestion. We have added the subsidiary drugs in line 152-153.*

In table 3, what timeline does sputum smear test pertain to? What is the definition of household economic burden? What is the definition for degree of symptoms at diagnosis? Is it self-reported or based on clinical assessment? The Operational definition of these variables need to be included in the methodology.

*Thanks for this suggestion. We have illustrated these definitions clearly in* ***table 1****.*

Why insurance coverage was not included as a factor for economic burden?

*Thanks for your suggestion. In our study, most of the study population were factory workers. Only 186 workers knew that they had medical insurance and 305 workers knew that they did not have any medical insurance. Other 23 workers were unaware of whether they had medical insurance or not, and at the end of the study, they did not bring about any medical care reimbursement cost. Only 34 patients provided information about medical care reimbursement cost. As a result, the proportion of cost paid by medical insurance was not included in the analysis.*

Among the factors considered for economic burden, some of them pertain to pre-diagnosis costs and other may pertain to treatment phase costs and few many be for total costs. It is recommended that these be split accordingly instead of considering everything together, as there are implications for suggesting possible interventions to reduce cost.

*We appreciate this suggestion. Considering everything together is truly not legible and easy to guide practice. So we decided to analyze the factors associated with pre-diagnosis and post-diagnosis separately according to your suggestion.*

Discussion

While the population of the district has 65% migrants, in the study, 95% of the study participants are migrants. This can have implications for representativeness of the study. This needs to be discussed in detail; this again importance further since it is one of the factors found to be significantly associated with economic burden as in table 3.

*Thanks for this suggestion. We have discussed it carefully in line 402-405 as follows: As one of the most population of Shenzhen City and owing numbers of factories , the migrant population in Bao’an district always exceeds the average level of Shenzhen City. The total number of registered active tuberculosis cases were 1317, included 76(5.8%) native case and 1241(94.2%) migrant cases in 2013. In our study, 95 percent were migrant cases, so our study can reflect the TB condition of Bao’an district truly.*

Socio-demographic and clinical characteristics of those 19 participants lost to follow-up need to be reported and the implications of the study results need to be discussed

*We appreciate this suggestion. In line 395-401, we have added the statement of the influence of lost follow-ups on our outcome.*

Of the 533 participants, 176 patients were reported to have been shifted to CPC with no costs for patients during pre-diagnosis period. Can description of these transfers provided and included in study setting. How this lead to no delays and no costs are important for understanding the drivers of costs/economic burden.

*Thank you for this suggestion. We have added the detailed illustration in the study setting section in line 132-139.*

Line 413, what are the author establishing by stating ‘recall and reporting bias could not be avoided, even in this longitudinal design’ The statement is self-contradictory. May need to be revised

*Revised, thank you.*

Figure may not be needed as this information is represented in the table 2 and is clearly evident too.

*We appreciate this suggestion. We deleted figure 1 in the article.*

Some Typographical Errors

Alignment of 70% in Age category 40-50 in Table 1

Subtitle Insurance to be made bold in Table 1

Weblink in line 112 may be shifted to reference and reference number provided

Title of Table 3, CNY needs to be removed

*Revised, thank you.*

Reviewer #2: 1. The author needs to add some more details on opportunity cost i.e, income loss by patients due to illness. Some patients were hospitalized but they have earnings from job without wage loss then how to quantify the opportunity cost? Also, variation in salary is very heterogenetic. The author needs to describe about opportunity cost.

*We appreciate this suggestion. We have describe the opportunity cost in* ***table 1*** *carefully. If TB patients still worked during the hospitalization, then there was no wage loss and the wage loss was 0.*

2. Under background characteristics, author mentioned about insurance company, but for Out of Pocket Expenditure (OOPE), we should not mention reimbursements given by insurance company. If the patients are getting reimbursements from any type of insurance, it should be deducted from the total out of pocket expenditure.

Net out of pocket expenditure = Total expenditure - Reimbursement.

Reimbursement amount should be highlighted if possible.

*Thank you for this suggestion. We realized that it was inappropriate to use the term ’out of pocket expenditure’ to describe the economic burden for in our study, the cost covered by medical insurance is not included in the analysis. In our study, most of the study population were factory workers. Only 186 workers knew that they had medical insurance and 305 workers knew that they did not have any medical insurance. Other 23 workers were unaware of whether they had medical insurance or not, and at the end of the study, they did not bring about any medical care reimbursement cost. Only 34 patients provided information about medical care reimbursement cost. As a result, the proportion of cost paid by medical insurance was not included in the analysis.*

3. In methodology section, the author has mentioned the Chi-square test and Mann-Whitney analysis to examine the effect of each predictor variable on economic burden. But Chi-square test is used to know the association between predictor variables and regression analysis is used to understand the effects of predictor variables.

*We appreciate your suggestion. We have revised the analysis method and Chi-square test is not used this time. So we deleted it.*

4. Table 3 analysis indicated that factors related to direct and opportunity costs (CNY) and in relation with the background variables. The heading should be association or differentials by background characteristics of patients and table 4 is showing the factor with regression analysis.

*We have revised the table titles according to your advice and thanks for your advice.*

5. In table 4 multivariate analysis, only few predictor variables have been added. However, the author described in methodology section A forward stepwise approach was used to find the appropriate model. If P-value less than 0.05 is significant. It is good and recognised methods for adding the variables in regression analysis. Try also Variance inflation factor (VIF) to know the multicollinearity in predictor variables. If VIF less than 10, than the author can add the predictor variables and explore more variable as predictor variables.

*Thank you for this suggestion. We have adjusted the data analysis method according to your suggestion.*

Based on the above observations and looking into overall merit of the paper, few modifications in line of above points may be considered before sending it for publication.
