# Supplementary material for: Analysis of the economic burden of diagnosis and treatment on patients with tuberculosis in Bao’an district of Shenzhen City, China
Source: PLoS One. 2020 Aug 31;15(8):e0237865. doi: 10.1371/journal.pone.0237865 (PMC7458315; doi:10.1371/journal.pone.0237865)
Supplement: Attachment — Submitted filename: Response to Reviewers 0619.docx [file pone.0237865.s008.docx]

**Title**

1. May remove 'non-drug resistance' from the title. In the study population may explain that you included people with TB (not known to be drug-resistant)

*Thank you for this suggestion. We have removed 'non-drug resistance' from the title and explain it clearly in the study population section.*

2. remove (TB) form title

*Revised, thank you.*

3. may remove 'non-demonstration area' from the title and mention the exact province or district in the title

*Revised, thank you.*

4,Analysis of the economic burden of diagnosis and treatment on patients with tuberculosis in xxxx, China

*We appreciate this suggestion. We have revised the title according to your suggestion.*

**Abstract**

1. Still in the abstract i do not get the folllowing information.What proportion of housholds incurred catastrophic costs due to

- TB diagnosis (pre-diagnosis)

- TB treatment (post-diagnosis)

- TB care (pre plus post)

You have not mentioned the factors associated with catastrophic costs.

*We appreciate this suggestion. Our study does have the limitation that we collected the range of the household's pre-TB annual income of every patient instead the exact number. We did not consider the catastrophic cost as an indicator in the design phase of our study, so we are not able to conduct the analysis regarding catastrophic cost. According to the reviewers’ suggestion, we adopted two new analysis methods to make our results more convincing.*

2. Expand abbreviations at first use. Once an abbreviation is used, continue using it instead of the full form.Please check this throughout the paper

*Revised, thank you.*

3. Factors associated with

*Revised, thank you.*

4. Give exact percentage and frequency in parenthesis

*Revised, thank you.*

5. Please main consistency pre-diagnosis direct post-diagnosis medical and

non-medical. BUT direct post-diagnosis? Shouldn't it be post-diagnosis direct costs?

post-diagnosis direct costs? I am suggesting the following structure to your results narrative in abstract in line with my suggestions for results narrative in main text

To avoid confusion, i suggest the following three important costs and presenting their median values here

costs due to TB diagnosis

costs due to TB treatment

costs due to TB care (diagnosis and treatment)

Then mention the % contribution of direct medical, direct non-medical, indirect costs to the above three costs. Then mention the key factors for high costs (due to diagnosis, due to treatment, due to care (diag+treat)). While mentioning the factors, instead of mentioning the variable, mention the exact sub category that was associated with high costs.

*Thanks for this suggestion. To make the description more accurate, we have revised the narrative regarding the cost occurred before and after diagnosis as follows:*

*We changed the ‘pre-diagnosis costs’ into ‘costs due to TB diagnosis’, ‘post-diagnosis costs’ into ‘costs due to TB treatment’ and ‘total costs’ into ‘costs due to TB care (diagnosis and treatment)’.*

6. Does this mean post-diagnosis or pre+post-diagnosis?

*We appreciate this suggestion. It means pre+post-diagnosis and in this revision we have changed it into ‘costs due to TB care (diagnosis and treatment)’.*

7. Not clear

Education being associated with pre-diagnosis costs. I am not clear whether those who are educated have more pre-diagnosis costs or those with not educated have more pre-diagnosis costs. In other words the diagnosis of association is not clear.

Please may modify as follows.

Factors associated with more pre-diagnosis costs are: xxx, yyy, zzz. (just do not mention education, mention whether it is less education or more education)

Similarly mention: Factors association with more post-diagnosis costs are: xxx, yyy, zzzz Like education, clarify for household registration (ye or no), sputum smear status (high grade or low grade, marital status (married or unmarried)

*We appreciate this suggestion. We have modified the illustration as follows (line 55-60):*

*Those who visited more times to health facilities, hospitalized, received higher education or occupied in national civil servant/services /retired staff might expense more before diagnosis. Factors Costs due to TB treatment among migrant patients, Sputum smear positive patients, widowed/divorced population, was significantly higher. Factors associated with less total costs were native patients, fewer times of visiting to health-care facilities and those with no hospitalization history due to TB.*

**Background**

1.What is the word count? from introduction to conclusion (excluding the tables and figures?).

I see this article to be very long. Though plos one does not have a word count limit, the article should be made more crisp and limited to 3000 words (max)

*Thanks for this suggestion. We revised the title ‘Introduction’ as ‘Background’. In order to state our results simpler and clear, we have deleted a lot of statement which is not pretty related to our research and put some tables as the supplement materials.*

2.These have to be updated as per WHO Global TB report 2019 (data for year 2018)

*Thanks for your suggestion and we have updated the data according to the Global TB report 2019.*

3.Not clear

*Thanks for this suggestion. To avoid confusion, we have deleted these words.*

**Materials and Methods**

1.What about assessment of catastrophic costs and factors associated with catastrophic costs?

*We appreciate your suggestion. As mentioned above, we did not consider the catastrophic cost as an indicator in the design phase of our study, so we are not able to conduct the analysis regarding catastrophic cost. We evaluated the economic burden as the* *proportion of patients’ total annual income instead.*

2.Expand abbreviations on first use.

*Revised, thank you.*

3.How is this related to the current study?

*We appreciate your suggestion. This part is not directly associated with our study and we have removed it from the article.*

4.Is this relevant to the current study?

*Removed, thank you.*

5.Use 'previously treated' throughout the manuscript - narrative text, tables and figures

*Revised, thank you.*

6.TB patients (not known to be drug resistant)

*Revised, thank you.*

7.Transferred-in patients

*Revised, thank you.*

8.Mention this line in measurements and definition section. May be at the beginning of second para in measurements and definiton section. Remove it from here.

*Thanks for your suggestion and we have removed it from here according to your advice.*

9.What did you do when there was unfavourable outcomes, say loss to follow up? did you use monthly average of previous months and added it to the reminder of the treatment. What was done when a patient was transferred out? Transfer out is common among migrants.

*We appreciate your suggestion. The present study surveyed 533 eligible patients, 19 of whom were lost to follow-up, one of whom died from another disease, and 18 of whom moved to other provinces, the lost rate nearly 4%. Considering the lost rate was low, those patients were eliminated directly in the analysis.*

10.May start this para by refering to Table 1. May put the details of costs calculation in Table 1. May mention the salient features here to avoid duplication. What about calculation of catastrophic costs. It has not been included in narrative or in the table.

The details of costs calculation were showed in the table 1.

*Revised, thank you.*

11.May remove this.

*Revised, thank you.*

12.May mention (indirect costs) in brackets.

*Revised, thank you.*

13.What is TP?

*Thanks for your suggestion. We made a typo and have revised it as ‘TB’.*

14.I guess this may be removed as well.

*Revised, thank you.*

15.I think you used median, may state so.

*Revised, thank you.*

16.Statement not complete. Please check.

*Revised, thank you.*

17.Please mention the approval number / letter number and date in parenthesis.

*We are grateful for your suggestion. We have added the ethical approval number in the article.*

**Results**

1.As mentioned before, the article is too long. One example is results narrative text.

The results narrative text should focus on the key results which should be in line with the aim and objectives. For details, the authors may always refer to the tables.

I suggest the authors to go through this paper for presentation of results. https://pubmed.ncbi.nlm.nih.gov/30173603/

Table 2 - baseline characteristics table

(see table 2 of above paper)

Table 3 - Table summarising the break of costs, three columns, costs due to diagnosis, treatment, diagnosis+treatment (see table 3 of above paper)

Table 4 - Contribution of direct medical, direct non-medical and indirect costs to the costs (in %), three columns as in Table 3 ((see table 3 of above paper)

Table 5,6,7: Factors assoc with all three costs - total costs due to diagnosis,treatment, diagnosis and treatment. (one table for each). See this paper to understand how these tables should be formatted (see table 3-8) https://bmchealthservres.biomedcentral.com/articles/10.1186/s12913-018-3583-y

Then in Table 8, my summarise the factors. See table S1 (Additional file 1) in this paper.

https://bmchealthservres.biomedcentral.com/articles/10.1186/s12913-018-3583-y

Table five, six, seven may also be put as supplementary table as one cannot interpret the B coefficient much as the data is log transformed. I do not see the factors associated with total costs more than the median value (logistic regression). ?

This was one of my suggestions in previous round. In the revised version, the authors have mention this in data analysis section but have not incuded the same in results narrative. May compare the risk factors in linear regression and logistic regresion. Your results narrative may tell variables that are significanlty associated in both the models (and esp across diagnosis, treatment and diag+treat costs). Focus your discussion section on these variables.

*Thanks for your suggestion. We have read the reference carefully and rearranged all the tables in this article. We summarize the factors in the supplement material in Table S4.*

*We also added the analysis regarding the factors associated with total cost according to your suggestion.*

2.While writing the results, main the following consistency either % (number) or number (%) or only %. Please do not mix, it becomes difficult to read.

*Thank you for this suggestion. We have revised the description of results with %(number) throughout the article.*

3.As shared before it is not clear what the final sample size of the study population

is? The same sample size should be the denominator for all your analysis.

Previous table sample size was around, 500, here it is 338. This is not the correct way to present. If people did not incur direct costs, mention their costs as zero and include them in the analysis.

*We appreciate your suggestion. In the new tables, we have included all the patients into the analysis.*

4.Those who did not incur pre-diagnosis costs should also be included. In other words, the denominator for calculation of costs has to be the same.

*Revised, thank you.*

5.Denominator has to be consistent as 514. If someone did not have costs under a

heading then consider it as zero and include it in the calculation of mean/median. See table 3 in https://pubmed.ncbi.nlm.nih.gov/30173603/

Instead of these details i would like to know in the form of a table or a 100% component bar diagram, what was the percentage contribution of direct medical, direct non-medical and indirect costs to total costs in the pre-diagnosis period, post diagnosis period and pre-post combined. See table 4 in <https://pubmed.ncbi.nlm.nih.gov/30173603/>

*Thanks for your suggestion. We have added the table 4 to present the percentage contribution of direct medical, direct non-medical and indirect costs to total costs in the pre-diagnosis period, post diagnosis period and pre-post combined.*

6.Not clear.when in hospital (ever? anytime during diagnosis?)

*Thanks for your suggestion. We have revised the category as ‘Whether in hospital due to TB diagnosis’.*

7.Only significant variables presented? if yes, clarify in footnotes. For B coefficient, mention in footnote that linear regression was done after conversion to log scale. Hence, cannot be directly interpreted. but significance can be assessed.

*We are grateful for your suggestion. We present the results of logistics*

*regression in the supplement table 1-3 and clarified it in the footnotes.*

8.Predictor?

*Revised, thank you.*

9.Can exclude from table.

*Revised, thank you.*

10.E should be capital

*Revised, thank you.*

11.Similar comments as in Table 4. This table is well formated though

*Revised, thank you.*
