# Supplementary material for: Analysis of the economic burden of diagnosis and treatment on patients with tuberculosis in Bao’an district of Shenzhen City, China
Source: PLoS One. 2020 Aug 31;15(8):e0237865. doi: 10.1371/journal.pone.0237865 (PMC7458315; doi:10.1371/journal.pone.0237865)
Supplement: Attachment — Submitted filename: Response to reviewers 0725.docx [file pone.0237865.s009.docx]

EDITOR COMMENTS

1.Table 2. Mention N=524 in title
*Thanks for your suggestion and we have added the number in the title of table 2.*

2.Table 3 and 4 - format the column titles
*We are grateful for this suggestion. In the last round of revision, the editor provided us with an article to refer to and we have rearranged the format of table 3 and 4 as the tables in the article (The table in this picture is one example). We have also revised the first column title of these two tables according to your suggestion.*

3.Do not repeat the results data in discussion. Results section is "what we found". Discussion section is "what it means"
Discussion section - please have a relook considering the principles below
First para - do not repeat the results. First para should include summary of results narrative using simple language (Without numbers) in 3-4 lines. When in doubt about the key findngs, revert back to the objectives
Then each finding should be discussed in one para. If three key findings, then three para.
Then in next para, what are the policy/practice implications and future research。
Strenght / Limitation
Please feel free to have these subheadings in discussion section: Summary of key findings, Discussion of key findings, Policy and practice implications, Strengths and limitations.
*We are grateful for your suggestion and have reorganized the structure and content of the discussion section. We deleted the redundant description of results and added more summaries of the key findings.*

REVIEWER 3 COMMENTS (they are in the form of an attachement, i am copying them here as well)
OVERALL COMMENTS
The study provide useful information about the economic burden of TB cases in Shenzhen city in China, which reflect the key indicator of the End- TB strategy. In spit the data was not so fresh ,the method and the definition was not in line with the latest TB patient cost handbook issued by WHO, the results still have some reference value for the evaluate of the progress in the TB control in south of China.

SPECIFIC COMMENT
1． Line 41：to replace the sentence“Illness-related costs experienced by patients” with “Illness-related costs experienced by tuberculosis patients” may be more related to the title

*Thanks for this suggestion. We have revised the sentence according to your suggestion.*

2． Line 43: “ full costs, including direct and opportunity costs” and Line 87 “opportunity costs ”is not clear, we recommend to use indirect cost directly and you use it in Table1.

*Thank you for this suggestion and we have replaced the ‘opportunity costs’ with ‘indirect costs’ throughout this article.*

3. Line 96-97 the reference of the migrant population proportion and income was inconsistent, considering the study was carried out in 2013, the statistical yearbook 2013 will be better.

*We are grateful for this suggestion. We have revised the year and added the data of 2013 in the article.*

4. Line 104 “The hospitals”is not accurate, here the hospital means general hospital?

*Thank you for this suggestion. Under the current DOTS strategy in China, TB case were detected through passive case finding method. Most suspected cases were discovered during their treatment in general hospitals and then sent to Center for Prevention and Cure of chronic diseases (CPC) by the health-care providers. So, the general hospitals were the main place to find TB cases. In our article, we have revised it as general hospitals.*

5. Line 114-115 the discription was not accurate, both the X-ray and sputum smear was carried out during diagnosis and treatment, please redescribe.

*We are grateful for this suggestion. To make the description more accurate, we have revised the statement as follows(line 113-116): The CPC is the institution authorized to provide TB diagnosis, treatment, and monitoring, with a radiologic imaging studies (X-ray) and sputum smear tests while diagnosed for free ,and 5 sputum smear tests and anti-TB drugs for 6-8 months during treatment for free.*

6. Line 119-120 The description of “The drugs prescribed every month including auxiliary examinations and subsidiary drugs like……. etc. are not free.” was obscure, may misleading reader that all the drugs including TB drugs was not free. Please clarify.

*We are grateful for your suggestion. We have revised this section and listed the drugs and service which are not free during the treatment clearly.*

8. Line 125 “1 January 2013 to June 2013” should be modified to “1 January 2013 to 30 June 2013”

*Revised. Thank you.*

9. Line128: the reason your study excluded the cases older than 59 was considering they might have more comorbidities, yet in the exclude criterions already mentioned the severe comobidities, so if it was necessary to exclude the elderly? Please reconsider.

*We are grateful for your suggestion. Besides the reason mentioned above, we also considered that the patients aged above 59 may have more have more respiratory system diseases and higher compliance which may affect the result of our research. To avoid this confounding factor, we excluded this age group. We have added this reason in the article.*

10. Line 161-163 the analysis did not included the income lost of companions for the few proportion and did not included the loss time for daily drug intake for the reason of most patients done this on their way to wort….., it would be better to clearly list the exact number replace “a few” or “most”, which will provide more exactly information to the reader. In my opinion , it was more rational to include this part in the analysis.

*We have used the exact number in this sentence and thank you for this suggestion.*

11. Line 199 : “19 of whom were lost to follow-up” and Line 201 “The rate of loss to follow-up was nearly 4% (19)” was repeat information, Please simplify after integration

*Thank you for this suggestion. We have revised the description as follows (line 205-207): The present study surveyed 533 eligible TB patients with no co-morbidity, 19 (4%) of whom were lost to follow-up, one of whom died from another disease, and 18 of whom moved to other provinces.*

12. Line 204-205 :“They were diagnosed at CPC immediately without consulting to other facilities, so their costs due to TB diagnosis were not calculated”. This information should be described in the method part instead of the results. Another question, for these cases the diagnosis fee should not be ignored considering they also should be confirmed diagnosis in the CPC.

*In our study, 176 patients were discovered during health check-ups required by their work units and the suspected cases were sent to the CPC. The CPC provided them with a radiologic imaging studies (X-ray) and sputum smear tests while diagnosed for free. As a result, these patients may not generate costs before diagnosis and we did not survey the costs occurred while diagnosed of this part of patients.*

13. Line 209: Under the title of Table 2, should give the N

*Revised. Thank you.*

14. Table 2 : Please recheck of the number and percentage of Age( years), the total number is only 401, please describe the age of other 103 cases, if the information was missing ,please clarify also.

*Thank you for this suggestion. We feel sorry that we made a mistake in this part and we have corrected it with the right number.*

15. Table 2: “self-reported economic status*” what was the meaning of *, please describe in the footnote.

*We are grateful for your suggestion. Under the table 2, we have added the footnote to describe the meaning of the symbol.*

16. Line 211: TB care( diagnosis +treatment), as you have defined the TB care in the method part, so it was not necessary to take the bracket here

*Revised. Thank you.*

17. Line 224 : Please use “indirect costs” to replace “opportunity costs” that will ensure the consistent with the describe in the Table3

*Thank you for this suggestion and we have replaced the ‘opportunity costs’ with ‘indirect costs’ throughout this article.*

18. The first line of Table3 and Table 4, Please use “TB care” to replace “TB diagnosis+treatment” and “TB care( diagnosis+treatment) ”

*We are grateful for your suggestion. We have revised the first line of these two tables according to your suggestion.*

19. Please recheck the ß value in Line 241 Line 245

*Revised. Thank you.*

20. Line 246 Table6??

*Thank you for this suggestion. The results here is presented in table S1, so we deleted the table 6 here and stated the correct table S1 at the end of this paragraph.*

21. For the discussion part, there were some repeat information of the results, such as Line 290 …. please go through this part and focus on the “real discussion”

*We are grateful for your suggestion. We have reorganized the structure and content of the discussion section according to your suggestion.*
